# Supplementary material for: Evaluation of the New Individual Fatty Acid Dataset for UK Biobank: Analysis of Intakes and Sources in 207,997 Participants
Source: Nutrients. 2022 Aug 31;14(17):3603. doi: 10.3390/nu14173603 (PMC9460581; doi:10.3390/nu14173603)
Supplement: Supplementary file 1 [file nutrients-14-03603-s001.zip › nutrients-1884724-supplementary/nutrients-1884724-rev1-suppl-210822.pdf]

Table S2. Description of the MCW food codes (n=8) with implausible individual FA values that were identified and corrected by tracing back to the original data source (n=2) or replaced with other MCW/USDA food codes (n=6).

Food code	Food code description	Issue	Resolution
12-344	Cheese, Brie, with outer rind removed	Total PUFAs were reported as 0.60 g/100g food; however, the sum of individual **PUFAs** was 0.97 g/100g food (1.62 times higher).	Replaced with Port Salut St Paulin type cheese (food code: 12-492) because this is a semi-soft cheese made with cow's milk and had similar fat subtotals to Brie with outer rind removed, and no other soft cheeses made with cow's milk in MCW had individual FA values.
11-822	Crispbread, rye	Palmitic acid (16:0) was reported as 15.27g/100g food; however, total SFAs were reported as only 0.16g/100g food.	Referred to original data from the 'DH, Nutrient analysis of biscuits, buns, cakes and pastries, 2011', which reported palmitic acid (16:0) was 0.15 g/100g food. This value was re-entered for palmitic acid (16:0) in the individual FA dataset.
17-704	Salad cream, reduced fat	Total PUFAs were reported as 5.63 g/100g food; however, the sum of individual PUFAs was 9.14 g/100g food (1.62 times higher).	Replaced with a recipe for low-fat salad cream based on supermarket products using the following ingredient foods and fractions: 9% rapeseed oil (food code: 17-041); 22% vinegar (17-339); 48% water (17-377); 16% white sugar (17-063); and 5% chicken egg yolk (12-939).
16-357	Salmon, farmed, flesh only, grilled	Total MUFAs were reported as 6.18 g/100g food; however, the sum of individual MUFAs was 43.58 g/100g food (7.05 times higher).	Replaced with steamed farm salmon with flesh only (food code: 16-358) because this was a similar food had similar fat subtotals.
11-993	Scones, fruit, retail	Total PUFAs were reported as 0.80 g/100g food; however, the sum of individual PUFAs was 1.65 g/100g food (2.1 times higher).	Replaced with plain retail scones (food code: 11-827) because this was a similar food and had similar fat subtotals.
16-361	Sushi, salmon nigiri	Total SFAs were reported as 0.48 g/100g food; however, the sum of individual SFAs was 1.09 g/100g food (2.27 times higher). Total MUFAs were reported as 1.00 g/100g food; however, the sum of individual MUFAs was 2.23 g/100g food (2.23 times higher). Total PUFAs were reported as 0.68 g/100g food; however, the sum of individual PUFAs was 1.22 g/100g food (1.79 times higher).	Replaced with a recipe for salmon nigiri sushi based on online recipes using the following ingredient foods and fractions: 70% boiled brown rice (food code: 11-869); and 30% raw wild salmon with flesh only (16-360).
11-925	Tortilla, wheat, soft	Total MUFAs were reported as 2.00 g/100g food; however, the sum of individual MUFAs was 4.03 g/100g food (2.02 times higher). Total PUFAs were reported as 0.80 g/100g food; however, the sum of individual PUFAs was 2.19 g/100g food (2.74 times higher).	Replaced with white English style muffins (food code: 11-541) because this better matched how the Oxford WebQ question was asked and there were no similar foods that had individual FA values in MCW.
12-184	Yogurt, whole milk, plain	Individual SFA values were reported as negative values per 100g food, which is not possible (e.g. butyric acid [4:0] was reported as -0.11g/100g food).	The sum of all individual SFAs if the values were assumed to be positive rather than negative was 1.92g/100g food, which is almost exactly the reported value for total SFAs (1.91g/100g food). Negative individual SFA values were re-entered as positive values in the individual FA dataset.

DH, department of health; FA, fatty acid; MCW, McCance and Widdowson; MUFA, monounsaturated fatty acid; PUFA, polyunsaturated fatty acid; SFA, saturated fatty acid; USDA, US Department of Agriculture.

Table S3. Description of USDA food codes (n=31) included in the individual FA dataset (MCW + USDA).

Food code	Food code description	Reason for using USDA food code	Included in the following food groups[1]
63105010	Avocado, raw	Guacamole, homemade (17-797) missing individual FAs. No similar food codes available in MCW. Assigned 85% to USDA food code in recipe replacement.	Vegetables and vegetable dishes (vegetable dips, vegetable dishes and meat substitutes)
		Avocado, average, flesh only (14-037) missing individual FAs. No similar food codes available in MCW. Replacement USDA food code has similar description and fat total and subtotals.	Vegetables and vegetable dishes (vegetables including legumes/pulses)
42104200	Cashew nuts, dry roasted	Cashew nuts, kernel only, roasted and salted (14-812) missing individual FAs. No similar food codes available in MCW. Replacement USDA food code has similar description and fat total and subtotals.	Nuts and seeds
		Nut roast, homemade (15-643) missing individual FAs. No similar food codes available in MCW. Assigned 15% to USDA food code in recipe replacement.	Vegetables and vegetable dishes (vegetable dips, vegetable dishes and meat substitutes)
75214010	Cauliflower, cooked, NS as to form, fat not added in cooking	Cauliflower, boiled in unsalted water (13-513) missing individual FAs. No similar food codes available in MCW. Replacement USDA food code has similar description and fat total and subtotals.	Vegetables and vegetable dishes (vegetables including legumes/pulses)
14410330	Cheese spread, American or Cheddar cheese base, reduced fat	Cheese, processed, slices, reduced fat (12-549) missing individual FAs. No similar food codes available in MCW. Replacement USDA food code has similar description and average of fat and fat subtotals for all cheese spreads being replaced.	Milk and milk products (cheese)
		Cheese, spreadable, medium fat, soft, white (12-538) missing individual FAs. No similar food codes available in MCW. Replacement USDA food code has similar description and average of fat and fat subtotals for all cheese spreads being replaced.	Milk and milk products (cheese)
		Cheese, spreadable, soft white, low fat (12-541) missing individual FAs. No similar food codes available in MCW. Replacement USDA food code has similar description and average of fat and fat subtotals for all cheese spreads being replaced.	Milk and milk products (cheese)
14410120	Cheese, American, reduced fat	Cheese, Cheddar type, half fat (12-348) missing individual FAs. No similar food codes available in MCW. Replacement USDA food code has similar description and fat total and subtotals.	Milk and milk products (cheese)
14201010	Cheese, cottage, creamed, large or small curd	Cheese, cottage, plain (12-539) missing individual FAs. No similar food codes available in MCW. Replacement USDA food code has similar description and fat total and subtotals.	Milk and milk products (cheese)
14204010	Cheese, cottage, low fat (1-2% fat)	Cheese, cottage, plain, reduced fat (12-550) missing individual FAs. No similar food codes available in MCW. Replacement USDA food code has similar description and fat total and subtotals.	Milk and milk products (cheese)
41302120	Chickpeas, canned, low sodium, fat not added in cooking	Pakora, vegetable, takeaway and restaurant (15-620) missing individual FAs. No similar food codes available in MCW. Assigned 40% to USDA food code in recipe replacement.	Miscellaneous (samosa, pakora)
		Beanburger, soya, fried in rapeseed oil (15-668) missing individual FAs. No similar food codes available in MCW. Assigned 47.5% to USDA food code in recipe replacement.	Vegetables and vegetable dishes (vegetable dips, vegetable dishes and meat substitutes)
		Soup, minestrone, homemade (17-810) missing individual FAs. No similar food codes available in MCW. Assigned 10% to USDA food code in recipe replacement.	Miscellaneous (soups)
		Falafel, fried in rapeseed oil, homemade (15-795) missing individual FAs. No similar food codes available in MCW. Assigned 90% to USDA food code in recipe replacement.	Vegetables and vegetable dishes (vegetable dips, vegetable dishes and meat substitutes)
		Houmous (13-556) missing individual FAs and fat subtotals. No similar food codes available in MCW. Assigned 58% to USDA food code in recipe replacement.	Vegetables and vegetable dishes (vegetable dips, vegetable dishes and meat substitutes)

		Beans, chickpeas, canned, re-heated, drained (13-560) missing individual FAs. No similar food codes available in MCW. Replacement USDA food code has similar description and fat total and subtotals.	Vegetables and vegetable dishes (vegetables including legumes/pulses)
		Beans, red kidney, canned in water, re-heated, drained (13-561) missing individual FAs. No similar food codes available in MCW. Replacement USDA food code has similar description and fat total and subtotals.	Vegetables and vegetable dishes (vegetables including legumes/pulses)
91705400	Chocolate, white	Chocolate, white (17-091) missing individual FAs. No similar food codes available in MCW. Replacement USDA food code has similar description and fat total and subtotals.	Sugars, preserves and sweets spreads (chocolate confectionery)
12210400	Cream substitute, powdered	Coffee mate, coffee whitener (12-546) missing individual FAs. No similar food codes available in MCW. Replacement USDA food code has similar description and fat total and subtotals.	Milk and milk products (semi skimmed milk)
41102220	Fava beans, cooked, fat not added in cooking	Beans, broad, whole, boiled in unsalted water (13-648) missing individual FAs. No similar food codes available in MCW. Replacement USDA food code has similar description and fat total and subtotals.	Vegetables and vegetable dishes (vegetables including legumes/pulses)
75111500	Garlic, raw	Garlic, raw (13-244) missing individual FAs. No similar food codes available in MCW. Replacement USDA food code has similar description and fat total and subtotals.	Vegetables and vegetable dishes (vegetables including legumes/pulses)
		Houmous (13-556) missing individual FAs and fat subtotals. No similar food codes available in MCW. Assigned 1% to USDA food code in recipe replacement.	Vegetables and vegetable dishes (vegetable dips, vegetable dishes and meat substitutes)
81204000	Ghee, clarified butter	Ghee, butter (17-640) missing individual FAs. No similar food codes available in MCW. Replacement USDA food code has similar description and fat total and subtotals.	Fat spreads (dairy fat spread)
28501010	Gravy, beef or meat, fat free	Gravy instant granules, made up with water (17-725) missing individual FAs. No similar food codes available in MCW. Assigned 95% to USDA food code in recipe replacement.	Miscellaneous (savoury sauces)
72119210	Kale, cooked, NS as to form, fat not added in cooking	Curly kale, boiled in unsalted water (13-649) missing individual FAs. No similar food codes available in MCW. Replacement USDA food code has similar description and fat total and subtotals.	Vegetables and vegetable dishes (vegetables including legumes/pulses)
41305000	Lentils, dry, cooked, fat not added in cooking	Beanburger, soya, fried in rapeseed oil (15-668) missing individual FAs. No similar food codes available in MCW. Assigned 47.5% to USDA food code in recipe replacement.	Vegetables and vegetable dishes (vegetable dips, vegetable dishes and meat substitutes)
		Nut roast, homemade (15-643) missing individual FAs. No similar food codes available in MCW. Assigned 30% to USDA food code in recipe replacement.	Vegetables and vegetable dishes (vegetable dips, vegetable dishes and meat substitutes)
		Lentils, red, split, dried, boiled in unsalted water (13-434) missing individual FAs. No similar food codes available in MCW. Replacement USDA food code has similar description and fat total and subtotals.	Vegetables and vegetable dishes (vegetables including legumes/pulses)
93301450	Liqueur with cream	Liqueurs, cream (17-768) missing individual FAs. No similar food codes available in MCW. Replacement USDA food code has similar description and fat total and subtotals.	Alcoholic beverages (spirits)
63129010	Mango, raw	Mangoes, ripe, flesh only, raw (14-378) missing individual FAs. No similar food codes available in MCW. Replacement USDA food code has similar description and fat total and subtotals.	Fruit
83204000	Mayonnaise, light	Tartare sauce (17-722) missing individual FAs. No similar food codes available in MCW. Replacement USDA food code has similar description and fat total and subtotals.	Miscellaneous (savoury sauces)
		Mayonnaise, reduced fat (17-679) missing individual FAs. No similar food codes available in MCW. Replacement USDA food code has similar description and fat total and subtotals.	Miscellaneous (savoury sauces)
11350000	Milk, almond, ready-to-drink	Added to all food items containing *milk_riceoatveg and *milk_other to better match how the question was asked in the Oxford WebQ. Food code for almond milk not available in MCW. Food codes for oat milk and rice milk are not available in MCW or USDA.	Milk and milk products (semi skimmed milk)

11114330	Milk, cow's, fluid, lactose reduced, 2% fat	Added to all food items containing *milk_other to better match how the question was asked in the Oxford WebQ. Food codes for lactose reduced or lactose free milks not available in MCW.	Milk and milk products (other milk and cream; semi skimmed milk)
11810000	Milk, dry, not reconstituted, NS as to whole, low fat, or non-fat	Dried skimmed milk (12-521) missing individual FAs. No similar food codes available in MCW. Replacement USDA food code has similar description and fat total and subtotals.	Milk and milk products (semi skimmed milk); cereals and cereal products (high fibre breakfast cereals)
11830260	Milk, malted, dry mix, fortified, not reconstituted, chocolate	Drinking chocolate, powder, reduced fat (17-499) missing individual FAs. No similar food codes available in MCW. Replacement USDA food code has similar description and fat total and subtotals.	Milk and milk products (other milk and cream)
		Horlicks, powder (17-731) missing individual FAs. No similar food codes available in MCW. Replacement USDA food code has similar description and fat total and subtotals.	Milk and milk products (other milk and cream)
		Ovaltine, powder (17-734) missing individual FAs. No similar food codes available in MCW. Replacement USDA food code has similar description and fat total and subtotals.	Milk and milk products (other milk and cream)
75510000	Olives, NFS	Olives, green, in brine, drained, flesh and skin (14-340) missing individual FAs. No similar food codes available in MCW. Replacement USDA food code has similar description and fat total and subtotals.	Miscellaneous (savoury sauces)
63133010	Papaya, raw	Papaya, flesh only, raw (14-390) missing individual FAs. No similar food codes available in MCW. Replacement USDA food code has similar description and fat total and subtotals.	Fruit
75222010	Parsnips, cooked, fat not added in cooking	Parsnip, boiled in unsalted water (13-631) missing individual FAs. No similar food codes available in MCW. Replacement USDA food code has similar description and fat total and subtotals.	Vegetables and vegetable dishes (vegetables including legumes/pulses)
42114130	Pistachio nuts	Pistachio nuts, kernel only, roasted and salted (14-840) missing individual FAs. No similar food codes available in MCW. Replacement USDA food code has similar description and fat total and subtotals.	Nuts and seeds
41420010	Soybean curd	Sushi, vegetable (15-617) missing individual FAs. No similar food codes available in MCW. Assigned 14% to USDA food code in recipe replacement.	Vegetables and vegetable dishes (vegetable dips, vegetable dishes and meat substitutes)
		Tofu, soya bean, steamed (13-570) missing individual FAs. No similar food codes available in MCW. Replacement USDA food code has similar description and fat total and subtotals.	Vegetables and vegetable dishes (vegetable dips, vegetable dishes and meat substitutes)
		Tofu, soya bean, steamed, fried (13-571) missing individual FAs. Assigned 87.5% to USDA food code in recipe replacement.	Vegetables and vegetable dishes (vegetable dips, vegetable dishes and meat substitutes)
72125212	Spinach, cooked, from frozen, fat not added in cooking	Spinach, mature, boiled in unsalted water (13-573) missing individual FAs. No similar food codes available in MCW. Replacement USDA food code has similar description and fat total and subtotals.	Vegetables and vegetable dishes (vegetables including legumes/pulses)
		Spinach, mature, raw (13-572) missing individual FAs. No similar food codes available in MCW. Replacement USDA food code has similar description and fat total and subtotals.	Vegetables and vegetable dishes (vegetables including legumes/pulses)
		Spinach, baby, raw (13-521) missing individual FAs. No similar food codes available in MCW. Replacement USDA food code has similar description and fat total and subtotals.	Vegetables and vegetable dishes (vegetables including legumes/pulses)
72125100	Spinach, raw	Watercress, raw (13-669) missing individual FAs. No similar food codes available in MCW. Replacement USDA food code has similar description and fat total and subtotals.	Vegetables and vegetable dishes (vegetables including legumes/pulses)
71201015	White potato chips, regular cut	Potato crisps, fried in sunflower oil (17-671) missing individual FAs. No similar food codes available in MCW. USDA code has similar description.	Savoury snacks (crisps and savoury snacks)

FA, fatty acid; MCW, McCance and Widdowson; NFS, not further specified; NS, not specified; USDA, US Department of Agriculture. [1] Food groups defined in Supplementary Table 5.

Table S4. Description of the individual FAs included in the new individual FA dataset (MCW + USDA).

Individual FAs	Systematic name	Common name	Values obtained from MCW[1]	Values obtained from USDA[2]
SFAs				
4:0	butanoic acid	butyric acid	4:0	4:0
6:0	hexanoic acid	caproic acid	6:0	6:0
8:0	octanoic acid	caprylic acid	8:0	8:0
10:0	decanoic acid	capric acid	10:0	10:0
12:0	dodecanoic acid	lauric acid	12:0	12:0
14:0	tetradecanoic acid	myristic acid	14:0	14:0
15:0	pentadecanoic acid	[systematic name used]	15:0	15:0
16:0	hexadecanoic acid	palmitic acid	16:0	16:0
17:0	heptadecanoic acid	[systematic name used]	17:0	17:0
18:0	octadecanoic acid	stearic acid	18:0	18:0
MUFAs				
16:1	hexadecenoic acid	palmitoleic acid	16:1 or 16:1c	16:0
18:1	octadecenoic acid	oleic acid	18:1 or 18:1c	18:0
20:1	eicosenoic acid	[systematic name used]	20:1 or 20:1c	20:1
22:1	docosenoic acid	[systematic name used]	22:1 or 22:1c	22:1
PUFAs				
n-3 PUFAs				
18:3[3]	octadecatrienoic acid	alpha-linolenic acid (ALA)	18:3 or 18:3-cis (n-3)	18:3
18:4[3]	octadecatetraenoic acid	stearidonic acid	18:4 or 18:4-cis (n-3)	18:4
20:5[3]	eicosapentaenoic acid (EPA)	[systematic name used]	20:5 or 20:5-cis (n-3)	20:5-cis (n-3)
22:5[3]	docosapentaenoic acid (DPA)	[systematic name used]	22:5 or 22:5-cis (n-3)	22:5-cis (n-3)
22:6[3]	docosahexaenoic acid (DHA)	[systematic name used]	22:6 or 22:6-cis (n-3)	22:6-cis (n-3)
n-6 PUFAs				
18:2[4]	octadecadienoic acid	linoleic acid (LA)	18:2 or 18:2-cis (n-6)	18:2
20:4[4]	eicosatetraenoic acid	arachidonic acid	20:4 or 20:4-cis (n-6)	20:4

FA, fatty acid; FNDDS, Food and Nutrient Database for Dietary Studies; MCW, McCance and Widdowson; MUFA, monounsaturated fatty acid; NNDSR, National Nutrient Database for Standard Reference; PUFA, polyunsaturated fatty acid; SFA, saturated fatty acid; USDA, US Department of Agriculture. [1] FAs obtained from McCance and Widdowson The Composition of Foods Integrated Dataset (2021). For individual MUFAs and PUFAs the FAs obtained are listed in order of preference (i.e. if the undifferentiated FA was missing then the isomer specific FA was selected). [2] FAs obtained from the USDA NNDSR Release 26 (2013). FAs from Release 26 correspond to the FAs included in the FNDDS (2011-2012) except for pentadecanoic acid (15:0) and heptadecanoic acid (17:0) because these FAs had incomplete values for some foods in the USDA NNDSR Release 26. [3] FA included in the calculation for n-3 PUFAs because the most typical FA isomer was cis (n-3). [4] FA included in the calculation for n-6 PUFAs because the most typical isomer was cis (n-6).

Table S5. Description of food groups used to examine dietary sources of individual FA intakes.

**Main food groups**[1]	**Includes the following subsidiary food groups**[2]
Cereals and cereal products	
Pasta and rice	White pasta and rice; wholemeal pasta, brown rice and other wholegrains
Pizza and other miscellaneous grains	Pizza; grain dishes (added fat)
White bread	White bread
Wholemeal bread	Wholemeal bread
Other bread	Mixed bread, brown and seeded; other bread
High fibre breakfast cereals	Bran cereal; biscuit cereal; oat cereal (non-sugar); oat cereal (sugar); muesli
Other breakfast cereals	Other cereal (sugar)
Biscuits	Biscuits
Savoury crackers	Savoury crackers
Desserts, cakes and pastries	Desserts, cakes and pastries
Milk and milk products	
Whole milk	Whole milk
Semi skimmed milk	Semi-skimmed milk
Skimmed milk	Skimmed milk
Other milk and cream	Rice/oat drink; soy drink; milk-based and powdered drinks; cream
Cheese	High fat cheese; medium and low fat cheese
Yoghurt	Full fat yogurt; low fat yogurt
Dairy desserts	Dairy desserts
Soya-based desserts and yogurt	Soya-based desserts and yogurt
Eggs and egg dishes	
Eggs and egg dishes	Eggs and egg dishes
Fat spreads[3]	
Olive oil	Olive oil (drizzling/dunking)
Dairy fat spread	Dairy fat spread lower fat; dairy fat spread
Vegetable spread	Vegetable-spread lower fat; vegetable spread
Meat and meat products	
Poultry	Poultry
Pork	Pork
Beef	Beef
Lamb	Lamb
Other meat	Other meat
Processed meat	Processed meat
Breaded/battered chicken	Breaded/battered chicken
Fish and fish dishes	
Breaded/battered fish	Breaded/battered fish
Other white fish, tinned tuna and shellfish	Other white fish and tinned tuna; shellfish
Oily fish	Oily fish
Vegetables and vegetable dishes	
Vegetables including legumes/pulses	Raw salad; green leafy/cabbages; root vegetables; tomatoes; allium vegetables; other vegetables (mushrooms, fruiting, mixed); peas/sweetcorn; legumes/pulses
Vegetable dips, vegetable dishes and meat substitutes	Vegetarian meals; soy-based meals; vegetable side dishes; vegetable dips
Fried/roast potatoes	Fried/roast potatoes
Baked/boiled/mashed potatoes	Potatoes/sweet potatoes (baked/boiled); mashed potatoes
Savoury snacks	
Crisps and savoury snacks	Savoury snacks
Nuts and seeds	
Nuts and seeds	Salted nuts and seeds; unsalted nuts and seeds; nut-based spreads
Fruit	
Fruit	Citrus; berries; apples and pears; bananas and other fruit; dried fruit; stewed fruit
Sugars, preserves and sweet spreads	
Sugars, preserves and sweet spreads	Added sugars and preserves

Sugar confectionery	Other sweets
Chocolate confectionery	Chocolate confectionery
Non-alcoholic beverages	
Fruit juice	Fruit juice
Soft drinks, not diet	SSBs and other sugary drinks
Soft drinks, diet	Low/non-sugar SSBs
Tea, coffee and water	Coffee, caffeinated; coffee, decaffeinated; tea; tea, decaffeinated; water/sparkling water
Alcoholic beverages	
Spirits and liqueurs	Spirits
Wine	White wine; red wine; fortified wine
Beer and cider	Beer and cider
Miscellaneous	
Soups	Soups
Savoury sauces	Sauces (higher fat); sauces (lower fat)
Samosa, pakora	Samosa, pakora
Sushi	Sushi

FA, fatty acid; NDNS, National Diet and Nutrition Survey; SSB, sugar-sweetened beverage. [1] Main food groups (14 primary food groups and 53 secondary food groups) were aggregated from subsidiary food groups to approximate the food groups in the NDNS years 1-4 combined (2011-2012), which were used to examine the dietary sources of individual FAs in this study. [2] Subsidiary food groups (n=93) that were included in the each of the main food groups. [3] Some oils which are used as a condiment on bread or salads are included in this food group; however, this food group does not include oils or fats used in cooking.

Table S6. Characteristics of 207,997 UK Biobank participants included in this study.

**Characteristics**	**Total**<br>**n = 207,997**	**Women**<br>**n = 114,406**	**Men**<br>**n = 93,591**
Age at recruitment (years)	56.1 (7.9)	55.7 (7.8)	56.6 (8.0)
White ethnicity, n (%)	198,700 (95.9%)	109,260 (95.6%)	89,440 (95.6%)
College or university degree, n (%)	145,025 (76.6%)	76,340 (73.2%)	68,685 (80.8%)
Alcohol (g/d)[1]	16.3 (17.2)	10.9 (10.6)	22.5 (20.9)
Current smoker, n (%)	16,147 (7.8%)	7,560 (6.6%)	8,587 (8.1%)
High physical activity, n (%)	30,460 (15.0%)	15,320 (13.7%)	15,157 (16.5%)
BMI (kg/m^2)	26.9 (4.6)	26.5 (5.0)	27.4 (4.1)

BMI, body mass index; g/d, grams per day; kg/m^2, kilogram per square metre; SD, standard deviation. Numbers are means (SD) unless otherwise specified as numbers (%), with % representing the column percentage excluding participants with missing values. [1] Excluding non-drinkers.

Table S7. Percentage contribution of food groups to intake of individual SFAs in the individual FA dataset (MCW + USDA) in 207,997 UK Biobank participants.

**Food groups**[1]	**Total SFAs**	**Percentage contribution of food groups to daily intake of**									
		**4:0**	**6:0**	**8:0**	**10:0**	**12:0**	**14:0**	**15:0**	**16:0**	**17:0**	**18:0**
Cereals and cereal products	23.72	16.71	16.34	22.18	20.82	29.62	18.79	18.28	27.72	16.88	18.25
Of which:											
Pizza and other miscellaneous grains	5.08	5.40	5.06	4.32	4.84	3.10	4.75	5.52	6.57	4.34	3.18
Other bread	0.57	0.32	0.46	1.12	0.69	1.45	0.57	0.57	0.56	0.52	0.39
High fibre breakfast cereals	1.51	1.21	1.32	2.95	2.17	4.16	1.71	0.93	1.53	1.48	0.89
Biscuits	5.80	3.74	3.34	3.50	3.27	4.79	3.81	4.81	6.86	4.65	5.72
Savoury crackers	0.83	0.00	0.12	1.59	0.63	2.44	0.54	0.09	1.05	0.12	0.33
Desserts, cakes and pastries	7.76	6.04	6.03	7.43	8.66	11.43	6.82	6.37	8.74	5.74	6.03
Milk and milk products	27.74	54.19	55.72	48.40	50.82	41.50	43.78	40.41	22.11	22.59	23.88
Of which:											
Whole milk	1.21	2.52	2.61	2.18	2.72	1.59	2.11	2.03	0.97	0.97	0.98
Semi skimmed milk	5.80	12.49	13.35	8.31	11.85	8.08	10.38	10.15	4.76	4.95	4.83
Other milk and cream	1.70	3.39	3.28	2.48	2.94	1.97	2.61	2.27	1.43	1.16	1.57
Cheese	11.65	26.67	28.48	21.82	22.07	11.50	18.76	18.21	9.08	10.49	9.22
Yoghurt	1.77	3.66	3.33	2.18	2.82	1.89	2.95	2.53	1.51	1.02	1.35
Dairy desserts	5.27	4.78	4.66	11.08	7.46	15.41	6.45	5.23	4.06	3.98	5.61
Eggs and egg dishes	3.14	1.67	1.59	1.14	1.39	0.81	1.50	1.95	3.83	7.01	3.51
Fat spreads	11.00	17.89	17.16	14.88	16.56	15.05	14.79	15.27	10.31	11.15	8.42
Of which:											
Dairy fat spread	8.66	17.89	17.10	13.09	15.72	10.41	13.78	15.02	7.37	10.52	6.71
Vegetable spread	2.19	<0.01	0.06	1.79	0.84	4.61	1.00	0.25	2.72	0.53	1.54
Meat and meat products	13.46	0.03	0.03	0.13	0.81	1.09	7.12	13.11	14.62	26.90	21.34
Of which:											
Poultry	1.25	<0.01	<0.01	<0.01	<0.01	0.04	0.26	0.10	1.58	0.75	1.45
Pork	0.90	<0.01	<0.01	<0.01	0.08	0.04	0.31	0.04	1.03	0.82	1.45
Beef	3.83	<0.01	<0.01	<0.01	0.06	0.07	2.29	8.36	3.80	14.02	6.55
Lamb	1.94	<0.01	<0.01	<0.01	0.33	0.59	2.17	3.77	1.60	6.12	3.52
Other meat	0.56	<0.01	<0.01	<0.01	<0.01	<0.01	0.17	<0.01	0.78	0.41	0.61
Processed meat	4.57	0.03	0.03	0.02	0.34	0.30	1.85	0.72	5.24	4.76	7.36
Fish and fish dishes	1.82	<0.01	<0.01	0.18	0.01	0.11	3.65	2.78	2.08	4.28	1.16
Of which:											
Oily fish	1.22	<0.01	<0.01	<0.01	<0.01	0.03	3.24	2.63	1.34	3.25	0.65
Vegetables and vegetable dishes	6.31	3.96	3.80	5.98	3.43	3.09	3.74	3.13	7.02	4.17	5.28

Of which:											
Vegetable dips, vegetable dishes and meat substitutes	0.62	0.05	0.04	0.08	0.07	0.10	0.16	0.07	0.69	0.56	0.76
Fried/roast potatoes	2.08	<0.01	<0.01	2.92	<0.01	0.20	0.48	0.45	2.88	1.52	1.90
Baked/boiled/mashed potatoes	1.97	3.09	2.93	2.33	2.65	2.22	2.38	2.01	1.74	1.46	1.54
Savoury snacks	1.33	<0.01	0.22	0.78	0.38	0.83	0.34	0.27	1.58	0.49	1.60
Nuts and seeds	2.13	<0.01	<0.01	0.43	0.25	0.79	0.59	<0.01	2.43	0.68	2.21
Sugars, preserves and sweet spreads	5.42	1.50	1.61	2.60	1.99	3.93	2.11	1.90	4.88	2.28	10.71
Of which:											
Chocolate confectionery	5.14	1.43	1.50	2.36	1.82	3.62	1.94	1.74	4.48	2.13	10.57
Miscellaneous	3.44	3.92	3.41	3.21	3.44	3.12	3.47	2.90	3.31	3.57	3.59
Of which:											
Soups	1.62	3.18	2.67	2.20	2.62	2.40	2.42	2.18	1.37	1.56	1.31
Savoury sauces	1.72	0.75	0.74	1.01	0.81	0.70	1.01	0.67	1.85	1.93	2.17

4:0, butyric acid; 6:0, caproic acid; 8:0, caprylic acid; 10:0, capric acid; 12:0, lauric acid; 14:0, myristic acid; 15:0, pentadecanoic acid; 16:0, palmitic acid; 17:0, heptadecanoic acid; 18:0, stearic acid; FA, fatty acid; MCW, McCance and Widdowson; SFA, saturated fatty acid; USDA, US Department of Agriculture. [1] Main food groups as described in Table S5. Food groups that contribute <0.5% to intake of total SFAs are excluded from the table. All other food groups are included.

Table S8. Percentage contribution of food groups to intake of individual MUFAs in the individual FA dataset (MCW + USDA) in 207,997 UK Biobank participants.

Food Groups[1]	Percentage contribution of food groups to daily intake of				
	Total MUFAs	16:1	18:1	20:1	22:1
Cereals and cereal products	21.33	10.20	21.95	13.83	6.08
Of which:					
Pizza and other miscellaneous grains	4.31	2.47	3.79	2.64	1.13
White bread	0.61	0.25	0.65	0.64	<0.01
Wholemeal bread	0.55	0.31	0.58	0.50	<0.01
Other bread	0.66	0.37	0.70	0.65	0.09
High fibre breakfast cereals	1.75	0.48	1.85	1.24	1.36
Biscuits	3.71	1.97	4.08	1.13	0.08
Savoury crackers	0.73	0.10	0.80	0.41	0.06
Desserts, cakes and pastries	7.09	3.73	7.48	5.52	2.80
Milk and milk products	10.75	18.06	9.52	16.60	0.08
Of which:					
Semi skimmed milk	2.10	3.46	1.98	0.01	<0.01
Other milk and cream	0.70	1.13	0.64	0.13	<0.01
Cheese	4.66	9.02	4.04	2.11	0.02
Yoghurt	0.66	1.19	0.54	0.23	<0.01
Dairy desserts	2.01	2.12	1.75	13.62	<0.01
Eggs and egg dishes	4.18	6.43	4.30	2.14	0.87
Fat spreads	9.90	7.37	10.07	7.30	4.34
Of which:					
Olive oil	0.78	0.25	0.85	0.17	<0.01
Dairy fat spread	5.48	6.46	5.30	4.24	2.29
Vegetable spread	3.64	0.66	3.92	2.88	2.06
Meat and meat products	15.81	33.85	15.65	9.97	1.17
Of which:					
Poultry	2.65	4.77	2.68	1.49	0.30
Pork	1.06	2.46	1.07	0.97	<0.01
Beef	3.70	10.00	3.55	0.11	0.17
Lamb	1.53	2.59	1.42	0.71	0.00
Other meat	0.88	2.06	0.88	0.70	0.38
Processed meat	5.10	9.99	5.14	5.36	0.08
Breaded/battered chicken	0.89	1.97	0.90	0.64	0.24
Fish and fish dishes	3.79	10.94	2.45	30.68	78.65
Of which:					

Breaded/battered fish	1.30	0.36	1.37	1.61	1.22
Oily fish	2.14	9.17	0.77	27.41	76.86
Vegetables and vegetable dishes	12.21	6.16	12.70	7.11	3.90
Of which:					
Vegetables including legumes/pulses	2.91	2.91	3.08	1.10	0.33
Vegetable dips, vegetable dishes and meat substitutes	2.22	0.63	2.33	1.90	1.61
Fried/roast potatoes	3.82	0.91	4.12	2.34	1.16
Baked/boiled/mashed potatoes	2.17	1.31	2.18	1.44	0.68
Savoury snacks	5.66	0.51	6.20	1.82	0.79
Nuts and seeds	5.92	1.72	6.43	2.98	0.20
Sugars, preserves and sweet spreads	3.40	1.05	3.60	1.11	0.17
Of which:					
Chocolate confectionery	3.24	0.98	3.42	1.06	0.17
Miscellaneous	6.76	3.45	7.03	6.45	3.75
Of which:					
Soups	1.17	1.38	1.14	0.88	0.32
Savoury sauces	5.43	1.95	5.74	5.20	2.79

16:1, palmitoleic acid; 18:1, oleic acid; 20:1, eicosenoic acid; 22:1 docosenoic acid; FA, fatty acid; MCW, McCance and Widdowson; MUFA, monounsaturated fatty acid; USDA, US Department of Agriculture. [1] Main food groups as described in Table S5. Food groups that contribute <0.5% to intake of total MUFAs are excluded from the table. All other food groups are included

Table S9. Percentage contribution of food groups to intake of individual PUFAs in the individual FA dataset (MCW + USDA) in 207,997 UK Biobank participants.

Food Groups[1]	Percentage contribution of food groups to daily intake of							
	Total PUFAs	18:2	18:3	18:4	20:4	20:5	22:5	22:6
Cereals and cereal products	23.50	25.33	22.36	1.32	9.95	4.08	2.55	0.36
Of which:								
Pasta and rice	1.06	1.29	0.36	<0.01	<0.01	<0.01	<0.01	<0.01
Pizza and other miscellaneous grains	3.48	2.92	3.33	<0.01	6.96	<0.01	<0.01	<0.01
White bread	1.55	1.80	1.36	<0.01	<0.01	<0.01	<0.01	<0.01
Wholemeal bread	1.65	1.91	1.58	<0.01	<0.01	<0.01	<0.01	<0.01
Other bread	1.29	1.31	2.12	<0.01	0.06	0.26	0.11	<0.01
High fibre breakfast cereals	2.71	3.22	1.28	<0.01	0.42	0.00	<0.01	<0.01
Biscuits	1.87	2.20	0.95	<0.01	0.69	1.74	0.72	0.12
Savoury crackers	0.73	0.87	0.31	<0.01	<0.01	<0.01	<0.01	<0.01
Desserts, cakes and pastries	6.32	6.52	9.21	1.06	1.72	0.80	0.93	0.18
Milk and milk products	3.62	3.40	4.66	0.45	5.04	19.24	7.27	0.01
Of which:								
Other milk and cream	0.87	0.81	0.80	0.45	0.24	0.31	0.16	<0.01
Cheese	1.29	1.14	2.17	<0.01	4.32	18.93	6.32	0.01
Dairy desserts	0.64	0.70	0.54	<0.01	0.48	<0.01	0.79	<0.01
Eggs and egg dishes	4.93	5.33	2.58	4.63	25.35	0.78	12.95	6.77
Fat spreads	9.95	10.23	13.28	1.13	4.03	10.31	4.74	<0.01
Of which:								
Dairy fat spread	3.43	3.07	6.03	1.13	3.28	10.31	4.74	<0.01
Vegetable spread	6.33	6.93	7.12	<0.01	0.75	<0.01	<0.01	<0.01
Meat and meat products	10.33	10.60	7.86	2.24	29.10	31.24	20.14	3.48
Of which:								
Poultry	3.30	3.68	2.03	0.02	5.32	0.14	4.43	1.47
Pork	0.68	0.69	0.31	<0.01	4.97	<0.01	1.72	0.06
Beef	0.98	0.88	1.08	0.41	3.30	5.19	2.74	<0.01
Other meat	0.54	0.60	0.41	<0.01	0.42	<0.01	<0.01	<0.01
Processed meat	3.64	3.73	2.03	<0.01	12.43	16.67	6.87	1.70
Breaded/battered chicken	0.74	0.76	0.89	0.75	1.27	5.11	1.06	0.18
Fish and fish dishes	5.56	2.66	3.27	89.01	23.09	30.05	49.57	88.25
Of which:								
Breaded/battered fish	1.33	1.32	1.97	0.79	0.27	0.77	0.80	1.55

Other white fish, tinned tuna and shellfish	1.21	0.92	0.18	6.50	4.36	8.83	9.02	14.87
Oily fish	3.01	0.42	1.12	81.73	18.46	20.45	39.75	71.83
Vegetables and vegetable dishes	16.13	15.15	21.02	0.66	1.13	1.66	0.97	0.01
Of which:								
Vegetables including legumes/pulses	2.34	2.20	2.04	0.02	0.08	0.16	0.08	<0.01
Vegetable dips, vegetable dishes and meat substitutes	3.85	3.34	9.36	0.36	0.33	<0.01	0.02	0.01
Fried/roast potatoes	4.97	5.72	3.71	<0.01	0.00	<0.01	0.00	<0.01
Baked/boiled/mashed potatoes	2.23	2.20	2.63	0.27	0.55	1.45	0.79	<0.01
Savoury snacks	6.51	8.04	1.79	<0.01	0.05	<0.01	<0.01	<0.01
Nuts and seeds	10.00	11.10	10.27	<0.01	0.04	<0.01	<0.01	<0.01
Sugars, preserves and sweet spreads	1.12	1.35	0.57	<0.01	0.11	<0.01	<0.01	<0.01
Of which:								
Chocolate confectionery	1.05	1.26	0.55	<0.01	0.11	<0.01	<0.01	<0.01
Miscellaneous	6.88	6.68	11.51	0.57	1.95	1.95	1.81	1.12
Of which:								
Soups	0.83	0.71	1.43	0.24	0.78	1.18	0.79	0.19
Savoury sauces	5.63	5.53	9.87	0.12	0.96	0.33	0.39	0.16

18:2, linoleic acid; 18:3, alpha-linolenic acid; 18:4, stearidonic acid; 20:4, arachidonic acid; 20:5, eicosapentaenoic acid; 22:5, docosapentaenoic acid; 22:6, docosahexaenoic acid; FA, fatty acid; MCW, McCance and Widdowson; PUFA, polyunsaturated fatty acid; USDA, US Department of Agriculture. [1] Main food groups as described in Table S5. Food groups that contribute <0.5% to intake of total PUFAs are excluded from the table. All other food groups are included

Table S10. Dietary intakes of total energy and macronutrients by fifths, shaded cells depict participants categorised into the same (dark shading) or adjacent (light shading) quintile using the main dataset (UKNDB) and the individual FA dataset (MCW + USDA).

Main dataset (UKNDB)		Individual FA dataset (MCW + USDA)				
		Q1	Q2	Q3	Q4	Q5
**Total energy intake, kJ/d**						
	Q1	39686	1914	0	0	0
	Q2	1914	37029	2656	0	0
	Q3	0	2656	36305	2639	0
	Q4	0	0	2639	37061	1899
	Q5	0	0	0	1899	39700
**Fat, g/d**						
	Q1	38496	3083	20	1	0
	Q2	3073	34150	4342	33	1
	Q3	31	4289	32777	4489	14
	Q4	0	77	4433	33713	3376
	Q5	0	0	28	3363	38208
**SFAs, g/d**						
	Q1	37924	3658	18	0	0
	Q2	3633	33145	4792	29	0
	Q3	42	4744	32077	4724	13
	Q4	1	51	4686	33430	3431
	Q5	0	1	27	3416	38155
**MUFAs, g/d**						
	Q1	37533	4024	41	2	0
	Q2	4017	32049	5464	68	1
	Q3	48	5419	30538	5567	28
	Q4	2	105	5516	31845	4132
	Q5	0	2	41	4117	37438
**PUFAs[1], g/d**						
	Q1	35827	5441	287	31	14
	Q2	5464	27870	7627	585	53
	Q3	276	7555	25340	8097	333
	Q4	33	656	7791	26545	6573
	Q5	0	77	555	6341	34626
**n-3 PUFAs[2], g/d**						
	Q1	31724	7144	1657	688	387
	Q2	8514	21389	8212	2445	1039
	Q3	1207	11158	18732	8183	2320
	Q4	144	1801	11871	19920	7863
	Q5	11	107	1128	10363	29990
**n-6 PUFAs[3], g/d**						
	Q1	34547	6624	400	23	6
	Q2	6251	25087	9277	940	44
	Q3	704	8406	22327	9586	577
	Q4	90	1303	8533	23772	7901
	Q5	8	179	1063	7278	33071
**TFAs, g/d**						
	Q1	31374	8765	1160	232	69

	**Q2**	7465	22018	10398	1501	217
	**Q3**	2012	8195	20508	10022	863
	**Q4**	631	2231	8301	23156	7280
	**Q5**	118	390	1233	6688	33170
**Carbohydrates, g/d**						
	**Q1**	39213	2387	0	0	0
	**Q2**	2385	35729	3481	4	0
	**Q3**	2	3469	34641	3487	1
	**Q4**	0	14	3476	35548	2561
	**Q5**	0	0	2	2560	39037
**Protein, g/d**						
	**Q1**	38167	3387	43	3	0
	**Q2**	3421	33148	4943	80	7
	**Q3**	11	5008	31501	5028	52
	**Q4**	1	54	5092	32729	3723
	**Q5**	0	2	21	3759	37817

ALA, alpha-linolenic acid; DHA, docosahexaenoic acid; DPA, docosapentaenoic acid; EPA, eicosapentaenoic acid; FA, fatty acid; g/d, grams per day; kJ/d, kilojoules per day; LA, linoleic acid; MCW, McCance and Widdowson; MUFA, monounsaturated fatty acid; PUFA, polyunsaturated fatty acid; Q, quintile; SFA, saturated fatty acid; TFA, trans fatty acid; UKNDB, UK nutrient databank; USDA, US Department of Agriculture. [1] For the main dataset this is the sum of n-3 and n-6 PUFAs. [2] For the individual FA dataset this is the sum of 18:3 (ALA), 18:4 (stearidonic acid), 20:5 (EPA), 22:5 (DPA) and 22:6 (DHA). [3] For the individual FA dataset this is the sum of 18:2 (LA) and 20:4 (arachidonic)

Table S11. Range of total energy and major nutrient intakes within each fifth in the main dataset (UKNDB) and the individual FA dataset (MCW + USDA) in 207,997 UK Biobank participants.

Nutrients	Main dataset (UKNDB)					Individual FA dataset (MCW + USDA)				
	Q1	Q2	Q3	Q4	Q5	Q1	Q2	Q3	Q4	Q5
Total energy intake, kJ/d	≤6711	6712 - 7850	7851 - 8916	8917 - 10319	>10319.81	≤6568	6570 - 7691	7692 - 8749	8750 - 10129	>10130
Fat, g/d	≤50.22	50.23 - 63.31	63.32 - 75.99	76.00 - 92.62	>92.64	≤48.79	48.80 - 61.75	61.76 - 74.25	74.26 - 90.63	>90.65
SFAs, g/d	≤17.30	17.31 - 22.71	22.72 - 28.06	28.07 - 35.33	>35.34	≤16.49	16.50 - 21.76	21.77 - 26.95	26.96 - 33.97	>33.98
MUFAs, g/d	≤17.72	17.73 - 22.66	22.67 - 27.48	27.49 - 33.90	>33.91	≤17.95	17.96 - 23.13	23.14 - 28.17	28.18 - 34.84	>34.86
PUFAs[1], g/d	≤8.32	8.33 - 10.80	10.81 - 13.26	13.27 - 16.69	>16.70	≤8.19	8.20 - 10.57	10.58 - 12.91	12.92 - 16.10	>16.11
n-3 PUFAs[2], g/d	≤1.20	1.21 - 1.59	1.60 - 2.01	2.02 - 2.61	>2.62	≤1.06	1.07 - 1.50	1.51 - 1.96	1.97 - 2.61	>2.62
n-6 PUFAs[3], g/d	≤6.87	6.88 - 9.03	9.04 - 11.19	11.20 - 14.23	>14.24	≤6.23	6.24 - 8.23	8.24 - 10.18	10.19 - 12.89	>12.90
TFAs, g/d	≤0.65	0.66 - 0.94	0.95 - 1.22	1.23 - 1.62	>1.63	≤0.53	0.54 - 0.79	0.80 - 1.07	1.08 - 1.46	>1.47
Carbohydrates, g/d	≤192.5	192.6 - 229.9	230.0 - 264.2	264.3 - 309.0	>309.1	≤188.6	188.7 - 225.6	225.7 - 259.4	259.5 - 303.3	>303.4
Protein, g/d	≤62.07	62.08 - 73.50	73.51 - 83.70	83.71 - 96.97	>96.98	≤61.49	61.50 - 72.81	72.82 - 82.97	82.98 - 96.09	>96.10

ALA, alpha-linolenic acid; DHA, docosahexaenoic acid; DPA, docosapentaenoic acid; EPA, eicosapentaenoic acid; FA, fatty acid; g/d, grams per day; kJ/d, kilojoules per day; LA, linoleic acid; MCW, McCance and Widdowson; MUFA, monounsaturated fatty acid; PUFA, polyunsaturated fatty acid; Q, quintile; SFA, saturated fatty acid; TFA, trans fatty acid; UKNDB, UK nutrient databank; USDA, US Department of Agriculture. [1] For the main dataset this is the sum of n-3 and n-6 PUFAs. [2] For the individual FA dataset this is the sum of 18:3 (ALA), 18:4 (stearidonic acid), 20:5 (EPA), 22:5 (DPA) and 22:6 (DHA). [3] For the individual FA dataset this is the sum of 18:2 (LA) and 20:4 (arachidonic).

Table S12. Percentage contribution of food groups to intake of total SFAs, MUFAs, PUFAs and TFAs between the main dataset (UKNDB) and the individual FA dataset (MCW + USDA) in 207,997 UK Biobank participants.

Food Groups[1]	Main dataset (UKNDB)						Individual FA dataset (MCW + USDA)					
	Percentage contribution of food groups to daily intake of						Percentage contribution of food groups to daily intake of					
	SFAs	MUFAs	PUFAs[2]	n-3 PUFAs[3]	n-6 PUFAs[4]	TFAs	SFAs	MUFAs	PUFAs[2]	n-3 PUFAs[3]	n-6 PUFAs[4]	TFAs
Cereals and cereal products	23.52	21.12	23.12	18.67	23.93	14.37	23.72	21.33	23.50	18.86	25.16	12.66
Milk and milk products	28.73	10.53	3.49	3.77	3.44	36.05	27.74	10.75	3.62	4.39	3.42	34.55
Eggs & egg dishes	3.13	4.75	4.01	3.67	4.07	1.60	3.14	4.18	4.93	3.34	5.55	1.25
Fat spreads	10.67	9.89	9.42	10.35	9.26	9.43	11.00	9.90	9.95	11.39	10.16	10.49
Meat and meat products	13.85	15.88	9.91	9.60	9.96	22.88	13.46	15.81	10.33	8.01	10.80	19.19
Fish and fish dishes	2.08	3.61	5.42	18.80	2.98	2.40	1.82	3.79	5.56	15.33	2.88	0.19
Vegetables and vegetable dishes	6.09	12.36	19.76	18.43	20.01	6.20	6.31	12.21	16.13	17.61	14.99	3.46
Savoury snacks	1.40	5.69	3.63	1.21	4.07	0.35	1.33	5.66	6.51	1.50	7.95	0.44
Nuts & seeds	1.03	3.50	7.32	2.89	8.13	0.70	2.13	5.92	10.00	8.57	10.98	0.00
Fruit	0.01	0.01	0.08	0.14	0.06	0.00	0.42	0.27	1.46	0.67	0.13	0.00
Sugars, preserves & sweet spreads	4.97	3.31	0.89	0.33	1.00	1.10	5.42	3.40	1.12	0.48	1.34	0.98
Non-alcoholic beverages	0.02	0.01	0.10	0.21	0.08	0.00	0.00	0.00	0.00	0.00	0.00	0.00
Alcoholic beverages	0.06	0.02	0.00	0.01	0.00	0.07	0.06	0.03	0.01	0.02	0.01	0.00
Miscellaneous	3.03	6.06	8.32	8.35	8.32	4.84	3.44	6.76	6.88	9.81	6.62	4.18

ALA, alpha-linolenic acid; DHA, docosahexaenoic acid; DPA, docosapentaenoic acid; EPA, eicosapentaenoic acid; FA, fatty acid; g/d, grams per day; LA, linoleic acid; MCW, McCance and Widdowson; MUFA, monounsaturated fatty acid; PUFA, polyunsaturated fatty acid; SFA, saturated fatty acid; TFA, trans fatty acid; UKNDB, UK nutrient databank; USDA US Department of Agriculture. [1] Primary food groups as described in Supplementary Table 5. [2] For the main dataset this is the sum of n-3 and n-6 PUFAs. [3] For the individual FA dataset this is the sum of 18:3 (ALA), 18:4 (stearidonic acid), 20:5 (EPA), 22:5 (DPA) and 22:6 (DHA). [4] For the individual FA dataset this is the sum of 18:2 (LA) and 20:4 (arachidonic).

Supplementary Figures

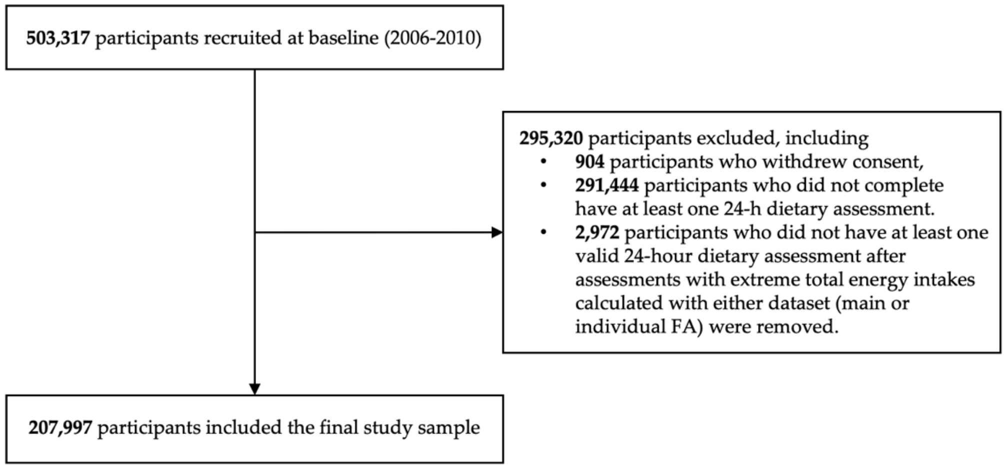

Figure S1. Flow chart of UK Biobank participants included in this study to assess nutrient intakes estimated from the individual FA dataset (n=207,997). FA fatty acid.
