# Supplementary material for: Evaluation of the New Individual Fatty Acid Dataset for UK Biobank: Analysis of Intakes and Sources in 207,997 Participants
Source: Nutrients. 2022 Aug 31;14(17):3603. doi: 10.3390/nu14173603 (PMC9460581; doi:10.3390/nu14173603)
Supplement: Supplementary file 1 [file nutrients-14-03603-s001.zip › nutrients-1884724-supplementary/nutrients-1884724-rev1-TableS1-210822.pdf]

**Table S1.** Individual FA and major nutrient calculation in the individual FA dataset (MCW + USDA). USDA food codes are highlighted in red.

| Food item | Grams per 100g of food item[1] | | | | Codes for foods from MCW and USDA and the fraction (%) assigned to each code[2] | | | | | | | | | | | | | | | | | | | | | |
|---|---|---|---|---|---|---|---|---|---|---|---|---|---|---|---|---|---|---|---|---|---|---|---|---|---|---|
| | Total fat | Total SFAs | Total MUFAs | Total PUFAs | Code 1 | Fraction 1 | Code 2 | Fraction 2 | Code 3 | Fraction 3 | Code 4 | Fraction 4 | Code 5 | Fraction 5 | Code 6 | Fraction 6 | Code 7 | Fraction 7 | Code 8 | Fraction 8 | Code 9 | Fraction 9 | Code 10 | Fraction 10 | Code 11 | Fraction 11 |
| add_salt | 0.00 | 0.00 | 0.00 | 0.00 | 17-367 | 100 | | | | | | | | | | | | | | | | | | | | |
| alcohol_beercider | 0.00 | 0.00 | 0.00 | 0.00 | 17-749 | 35 | 17-506 | 35 | 17-222 | 12 | 17-224 | 18 | | | | | | | | | | | | | | |
| alcohol_other | 5.24 | 3.23 | 1.49 | 0.22 | 93301450 | 33.4 | 17-245 | 33.3 | 17-244 | 33.3 | | | | | | | | | | | | | | | | |
| alcohol_spirits | 0.00 | 0.00 | 0.00 | 0.00 | 17-247 | 100 | | | | | | | | | | | | | | | | | | | | |
| alcohol_wine_fort | 0.00 | 0.00 | 0.00 | 0.00 | 17-236 | 50 | 17-234 | 50 | | | | | | | | | | | | | | | | | | |
| alcohol_wine_red_large | 0.00 | 0.00 | 0.00 | 0.00 | 17-752 | 100 | | | | | | | | | | | | | | | | | | | | |
| alcohol_wine_red_med | 0.00 | 0.00 | 0.00 | 0.00 | 17-752 | 100 | | | | | | | | | | | | | | | | | | | | |
| alcohol_wine_red_small | 0.00 | 0.00 | 0.00 | 0.00 | 17-752 | 100 | | | | | | | | | | | | | | | | | | | | |
| alcohol_wine_rose_large | 0.00 | 0.00 | 0.00 | 0.00 | 17-754 | 100 | | | | | | | | | | | | | | | | | | | | |
| alcohol_wine_rose_med | 0.00 | 0.00 | 0.00 | 0.00 | 17-754 | 100 | | | | | | | | | | | | | | | | | | | | |
| alcohol_wine_rose_small | 0.00 | 0.00 | 0.00 | 0.00 | 17-754 | 100 | | | | | | | | | | | | | | | | | | | | |
| alcohol_wine_white_large | 0.00 | 0.00 | 0.00 | 0.00 | 17-755 | 25 | 17-756 | 25 | 17-757 | 25 | 17-233 | 25 | | | | | | | | | | | | | | |
| alcohol_wine_white_med | 0.00 | 0.00 | 0.00 | 0.00 | 17-755 | 25 | 17-756 | 25 | 17-757 | 25 | 17-233 | 25 | | | | | | | | | | | | | | |
| alcohol_wine_white_small | 0.00 | 0.00 | 0.00 | 0.00 | 17-755 | 25 | 17-756 | 25 | 17-757 | 25 | 17-233 | 25 | | | | | | | | | | | | | | |
| biscuit_choc | 24.40 | 11.77 | 8.68 | 2.43 | 11-815 | 25 | 11-807 | 50 | 11-816 | 25 | | | | | | | | | | | | | | | | |
| biscuit_choc_gf | 24.40 | 11.77 | 8.68 | 2.43 | 11-815 | 25 | 11-807 | 50 | 11-816 | 25 | | | | | | | | | | | | | | | | |
| biscuit_choccov | 26.81 | 15.61 | 8.11 | 1.50 | 11-810 | 34 | 17-657 | 33 | 11-812 | 33 | | | | | | | | | | | | | | | | |
| biscuit_choccov_gf | 26.81 | 15.61 | 8.11 | 1.50 | 11-810 | 34 | 17-657 | 33 | 11-812 | 33 | | | | | | | | | | | | | | | | |
| biscuit_sweet | 22.00 | 10.80 | 7.72 | 1.75 | 11-799 | 33.4 | 11-798 | 33.3 | 11-802 | 33.3 | | | | | | | | | | | | | | | | |
| biscuit_sweet_gf | 22.00 | 10.80 | 7.72 | 1.75 | 11-799 | 33.4 | 11-798 | 33.3 | 11-802 | 33.3 | | | | | | | | | | | | | | | | |
| brdbag_s_butter_dunno_med | 80.65 | 43.12 | 25.23 | 6.42 | 17-655 | 50 | 17-685 | 25 | 17-661 | 25 | | | | | | | | | | | | | | | | |
| brdbag_s_butter_dunno_thick | 80.65 | 43.12 | 25.23 | 6.42 | 17-655 | 50 | 17-685 | 25 | 17-661 | 25 | | | | | | | | | | | | | | | | |
| brdbag_s_butter_dunno_thin | 80.65 | 43.12 | 25.23 | 6.42 | 17-655 | 50 | 17-685 | 25 | 17-661 | 25 | | | | | | | | | | | | | | | | |
| brdbag_s_butter_fat_med | 82.20 | 52.09 | 20.91 | 2.83 | 17-685 | 50 | 17-661 | 50 | | | | | | | | | | | | | | | | | | |
| brdbag_s_butter_fat_thick | 82.20 | 52.09 | 20.91 | 2.83 | 17-685 | 50 | 17-661 | 50 | | | | | | | | | | | | | | | | | | |
| brdbag_s_butter_fat_thin | 82.20 | 52.09 | 20.91 | 2.83 | 17-685 | 50 | 17-661 | 50 | | | | | | | | | | | | | | | | | | |
| brdbag_s_butter_lowfat_med | 60.20 | 25.70 | 22.66 | 7.75 | 17-656 | 100 | | | | | | | | | | | | | | | | | | | | |
| brdbag_s_butter_lowfat_thick | 60.20 | 25.70 | 22.66 | 7.75 | 17-656 | 100 | | | | | | | | | | | | | | | | | | | | |
| brdbag_s_butter_lowfat_thin | 60.20 | 25.70 | 22.66 | 7.75 | 17-656 | 100 | | | | | | | | | | | | | | | | | | | | |
| brdbag_s_buttspr_fat_med | 79.10 | 34.15 | 29.54 | 10.00 | 17-655 | 100 | | | | | | | | | | | | | | | | | | | | |
| brdbag_s_buttspr_fat_thick | 79.10 | 34.15 | 29.54 | 10.00 | 17-655 | 100 | | | | | | | | | | | | | | | | | | | | |
| brdbag_s_buttspr_fat_thin | 79.10 | 34.15 | 29.54 | 10.00 | 17-655 | 100 | | | | | | | | | | | | | | | | | | | | |
| brdbag_s_buttspr_lowfat_med | 60.20 | 25.70 | 22.66 | 7.75 | 17-656 | 100 | | | | | | | | | | | | | | | | | | | | |
| brdbag_s_buttspr_lowfat_thick | 60.20 | 25.70 | 22.66 | 7.75 | 17-656 | 100 | | | | | | | | | | | | | | | | | | | | |
| brdbag_s_buttspr_lowfat_thin | 60.20 | 25.70 | 22.66 | 7.75 | 17-656 | 100 | | | | | | | | | | | | | | | | | | | | |
| brdbag_s_dairy_chol_med | 39.00 | 9.76 | 17.39 | 9.82 | 12-497 | 100 | | | | | | | | | | | | | | | | | | | | |
| brdbag_s_dairy_chol_thick | 39.00 | 9.76 | 17.39 | 9.82 | 12-497 | 100 | | | | | | | | | | | | | | | | | | | | |
| brdbag_s_dairy_chol_thin | 39.00 | 9.76 | 17.39 | 9.82 | 12-497 | 100 | | | | | | | | | | | | | | | | | | | | |
| brdbag_s_dairy_dunno_med | 49.80 | 12.69 | 23.67 | 10.86 | 12-497 | 50 | 12-504 | 50 | | | | | | | | | | | | | | | | | | |
| brdbag_s_dairy_dunno_thick | 49.80 | 12.69 | 23.67 | 10.86 | 12-497 | 50 | 12-504 | 50 | | | | | | | | | | | | | | | | | | |
| brdbag_s_dairy_dunno_thin | 49.80 | 12.69 | 23.67 | 10.86 | 12-497 | 50 | 12-504 | 50 | | | | | | | | | | | | | | | | | | |
| brdbag_s_dairy_fat_med | 60.60 | 15.61 | 29.95 | 11.90 | 12-504 | 100 | | | | | | | | | | | | | | | | | | | | |
| brdbag_s_dairy_fat_thick | 60.60 | 15.61 | 29.95 | 11.90 | 12-504 | 100 | | | | | | | | | | | | | | | | | | | | |
| brdbag_s_dairy_fat_thin | 60.60 | 15.61 | 29.95 | 11.90 | 12-504 | 100 | | | | | | | | | | | | | | | | | | | | |
| brdbag_s_dairy_lowfat_med | 39.00 | 9.76 | 17.39 | 9.82 | 12-497 | 100 | | | | | | | | | | | | | | | | | | | | |
| brdbag_s_dairy_lowfat_thick | 39.00 | 9.76 | 17.39 | 9.82 | 12-497 | 100 | | | | | | | | | | | | | | | | | | | | |
| brdbag_s_dairy_lowfat_thin | 39.00 | 9.76 | 17.39 | 9.82 | 12-497 | 100 | | | | | | | | | | | | | | | | | | | | |
| brdbag_s_dairy_vlowfat_med | 39.00 | 9.76 | 17.39 | 9.82 | 12-497 | 100 | | | | | | | | | | | | | | | | | | | | |
| brdbag_s_dairy_vlowfat_thick | 39.00 | 9.76 | 17.39 | 9.82 | 12-497 | 100 | | | | | | | | | | | | | | | | | | | | |
| brdbag_s_dairy_vlowfat_thin | 39.00 | 9.76 | 17.39 | 9.82 | 12-497 | 100 | | | | | | | | | | | | | | | | | | | | |
| brdbag_s_dunno_chol_med | 37.95 | 9.16 | 14.45 | 12.39 | 12-497 | 50 | 12-499 | 50 | | | | | | | | | | | | | | | | | | |
| brdbag_s_dunno_chol_thick | 37.95 | 9.16 | 14.45 | 12.39 | 12-497 | 50 | 12-499 | 50 | | | | | | | | | | | | | | | | | | |
| brdbag_s_dunno_chol_thin | 37.95 | 9.16 | 14.45 | 12.39 | 12-497 | 50 | 12-499 | 50 | | | | | | | | | | | | | | | | | | |
| brdbag_s_dunno_dunno_med | 48.93 | 11.78 | 19.14 | 15.46 | 12-497 | 25 | 12-499 | 25 | 12-503 | 25 | 12-504 | 25 | | | | | | | | | | | | | | |
| brdbag_s_dunno_dunno_thick | 48.93 | 11.78 | 19.14 | 15.46 | 12-497 | 25 | 12-499 | 25 | 12-503 | 25 | 12-504 | 25 | | | | | | | | | | | | | | |
| brdbag_s_dunno_dunno_thin | 48.93 | 11.78 | 19.14 | 15.46 | 12-497 | 25 | 12-499 | 25 | 12-503 | 25 | 12-504 | 25 | | | | | | | | | | | | | | |
| brdbag_s_dunno_fat_med | 59.90 | 14.41 | 23.83 | 18.54 | 12-503 | 50 | 12-504 | 50 | | | | | | | | | | | | | | | | | | |
| brdbag_s_dunno_fat_thick | 59.90 | 14.41 | 23.83 | 18.54 | 12-503 | 50 | 12-504 | 50 | | | | | | | | | | | | | | | | | | |
| brdbag_s_dunno_fat_thin | 59.90 | 14.41 | 23.83 | 18.54 | 12-503 | 50 | 12-504 | 50 | | | | | | | | | | | | | | | | | | |
| brdbag_s_dunno_lowfat_med | 37.95 | 9.16 | 14.45 | 12.39 | 12-497 | 50 | 12-499 | 50 | | | | | | | | | | | | | | | | | | |
| brdbag_s_dunno_lowfat_thick | 37.95 | 9.16 | 14.45 | 12.39 | 12-497 | 50 | 12-499 | 50 | | | | | | | | | | | | | | | | | | |
| brdbag_s_dunno_lowfat_thin | 37.95 | 9.16 | 14.45 | 12.39 | 12-497 | 50 | 12-499 | 50 | | | | | | | | | | | | | | | | | | |
| brdbag_s_dunno_vlowfat_med | 37.95 | 9.16 | 14.45 | 12.39 | 12-497 | 50 | 12-499 | 50 | | | | | | | | | | | | | | | | | | |
| brdbag_s_dunno_vlowfat_thick | 37.95 | 9.16 | 14.45 | 12.39 | 12-497 | 50 | 12-499 | 50 | | | | | | | | | | | | | | | | | | |
| brdbag_s_dunno_vlowfat_thin | 37.95 | 9.16 | 14.45 | 12.39 | 12-497 | 50 | 12-499 | 50 | | | | | | | | | | | | | | | | | | |
| brdbag_s_hardmarg_med | 76.40 | 26.41 | 34.00 | 12.21 | 12-500 | 100 | | | | | | | | | | | | | | | | | | | | |
| brdbag_s_hardmarg_thick | 76.40 | 26.41 | 34.00 | 12.21 | 12-500 | 100 | | | | | | | | | | | | | | | | | | | | |
| brdbag_s_hardmarg_thin | 76.40 | 26.41 | 34.00 | 12.21 | 12-500 | 100 | | | | | | | | | | | | | | | | | | | | |
| brdbag_s_olive_chol_med | 38.90 | 8.90 | 21.68 | 6.36 | 12-498 | 100 | | | | | | | | | | | | | | | | | | | | |
| brdbag_s_olive_chol_thick | 38.90 | 8.90 | 21.68 | 6.36 | 12-498 | 100 | | | | | | | | | | | | | | | | | | | | |
| brdbag_s_olive_chol_thin | 38.90 | 8.90 | 21.68 | 6.36 | 12-498 | 100 | | | | | | | | | | | | | | | | | | | | |
| brdbag_s_olive_dunno_med | 49.00 | 11.06 | 26.53 | 8.95 | 12-498 | 50 | 12-505 | 50 | | | | | | | | | | | | | | | | | | |
| brdbag_s_olive_dunno_thick | 49.00 | 11.06 | 26.53 | 8.95 | 12-498 | 50 | 12-505 | 50 | | | | | | | | | | | | | | | | | | |
| brdbag_s_olive_dunno_thin | 49.00 | 11.06 | 26.53 | 8.95 | 12-498 | 50 | 12-505 | 50 | | | | | | | | | | | | | | | | | | |
| brdbag_s_olive_fat_med | 59.10 | 13.22 | 31.37 | 11.53 | 12-505 | 100 | | | | | | | | | | | | | | | | | | | | |

**Table S1.** Individual FA and major nutrient calculation in the individual FA dataset (MCW + USDA). USDA food codes are highlighted in red.

| Food item | Grams per 100g of food item[1] | | | | Codes for foods from MCW and USDA and the fraction (%) assigned to each code[2] | | | | | | | | | | | | | | | | | | | | | |
|---|---|---|---|---|---|---|---|---|---|---|---|---|---|---|---|---|---|---|---|---|---|---|---|---|---|---|
| | Total fat | Total SFAs | Total MUFAs | Total PUFAs | Code 1 | Fraction 1 | Code 2 | Fraction 2 | Code 3 | Fraction 3 | Code 4 | Fraction 4 | Code 5 | Fraction 5 | Code 6 | Fraction 6 | Code 7 | Fraction 7 | Code 8 | Fraction 8 | Code 9 | Fraction 9 | Code 10 | Fraction 10 | Code 11 | Fraction 11 |
| brdbag_s_olive_fat_thick | 59.10 | 13.22 | 31.37 | 11.53 | 12-505 | 100 | | | | | | | | | | | | | | | | | | | | |
| brdbag_s_olive_fat_thin | 59.10 | 13.22 | 31.37 | 11.53 | 12-505 | 100 | | | | | | | | | | | | | | | | | | | | |
| brdbag_s_olive_lowfat_med | 38.90 | 8.90 | 21.68 | 6.36 | 12-498 | 100 | | | | | | | | | | | | | | | | | | | | |
| brdbag_s_olive_lowfat_thick | 38.90 | 8.90 | 21.68 | 6.36 | 12-498 | 100 | | | | | | | | | | | | | | | | | | | | |
| brdbag_s_olive_lowfat_thin | 38.90 | 8.90 | 21.68 | 6.36 | 12-498 | 100 | | | | | | | | | | | | | | | | | | | | |
| brdbag_s_olive_vlowfat_med | 38.90 | 8.90 | 21.68 | 6.36 | 12-498 | 100 | | | | | | | | | | | | | | | | | | | | |
| brdbag_s_olive_vlowfat_thick | 38.90 | 8.90 | 21.68 | 6.36 | 12-498 | 100 | | | | | | | | | | | | | | | | | | | | |
| brdbag_s_olive_vlowfat_thin | 38.90 | 8.90 | 21.68 | 6.36 | 12-498 | 100 | | | | | | | | | | | | | | | | | | | | |
| brdbag_s_other_med | 68.67 | 33.71 | 21.43 | 9.30 | 81204000 | 18 | 17-685 | 16 | 17-661 | 16 | 12-499 | 12.5 | 12-503 | 12.5 | 12-497 | 12.5 | 12-504 | 12.5 | | | | | | | | |
| brdbag_s_other_thick | 68.67 | 33.71 | 21.43 | 9.30 | 81204000 | 18 | 17-685 | 16 | 17-661 | 16 | 12-499 | 12.5 | 12-503 | 12.5 | 12-497 | 12.5 | 12-504 | 12.5 | | | | | | | | |
| brdbag_s_other_thin | 68.67 | 33.71 | 21.43 | 9.30 | 81204000 | 18 | 17-685 | 16 | 17-661 | 16 | 12-499 | 12.5 | 12-503 | 12.5 | 12-497 | 12.5 | 12-504 | 12.5 | | | | | | | | |
| brdbag_s_poly_chol_med | 36.90 | 8.55 | 11.50 | 14.96 | 12-499 | 100 | | | | | | | | | | | | | | | | | | | | |
| brdbag_s_poly_chol_thick | 36.90 | 8.55 | 11.50 | 14.96 | 12-499 | 100 | | | | | | | | | | | | | | | | | | | | |
| brdbag_s_poly_chol_thin | 36.90 | 8.55 | 11.50 | 14.96 | 12-499 | 100 | | | | | | | | | | | | | | | | | | | | |
| brdbag_s_poly_dunno_med | 48.05 | 10.88 | 14.60 | 20.07 | 12-499 | 50 | 12-503 | 50 | | | | | | | | | | | | | | | | | | |
| brdbag_s_poly_dunno_thick | 48.05 | 10.88 | 14.60 | 20.07 | 12-499 | 50 | 12-503 | 50 | | | | | | | | | | | | | | | | | | |
| brdbag_s_poly_dunno_thin | 48.05 | 10.88 | 14.60 | 20.07 | 12-499 | 50 | 12-503 | 50 | | | | | | | | | | | | | | | | | | |
| brdbag_s_poly_fat_med | 59.20 | 13.21 | 17.70 | 25.17 | 12-503 | 100 | | | | | | | | | | | | | | | | | | | | |
| brdbag_s_poly_fat_thick | 59.20 | 13.21 | 17.70 | 25.17 | 12-503 | 100 | | | | | | | | | | | | | | | | | | | | |
| brdbag_s_poly_fat_thin | 59.20 | 13.21 | 17.70 | 25.17 | 12-503 | 100 | | | | | | | | | | | | | | | | | | | | |
| brdbag_s_poly_lowfat_med | 36.90 | 8.55 | 11.50 | 14.96 | 12-499 | 100 | | | | | | | | | | | | | | | | | | | | |
| brdbag_s_poly_lowfat_thick | 36.90 | 8.55 | 11.50 | 14.96 | 12-499 | 100 | | | | | | | | | | | | | | | | | | | | |
| brdbag_s_poly_lowfat_thin | 36.90 | 8.55 | 11.50 | 14.96 | 12-499 | 100 | | | | | | | | | | | | | | | | | | | | |
| brdbag_s_poly_vlowfat_med | 36.90 | 8.55 | 11.50 | 14.96 | 12-499 | 100 | | | | | | | | | | | | | | | | | | | | |
| brdbag_s_poly_vlowfat_thick | 36.90 | 8.55 | 11.50 | 14.96 | 12-499 | 100 | | | | | | | | | | | | | | | | | | | | |
| brdbag_s_poly_vlowfat_thin | 36.90 | 8.55 | 11.50 | 14.96 | 12-499 | 100 | | | | | | | | | | | | | | | | | | | | |
| brdbag_s_soya_chol_med | 36.90 | 8.55 | 11.50 | 14.96 | 12-499 | 100 | | | | | | | | | | | | | | | | | | | | |
| brdbag_s_soya_chol_thick | 36.90 | 8.55 | 11.50 | 14.96 | 12-499 | 100 | | | | | | | | | | | | | | | | | | | | |
| brdbag_s_soya_chol_thin | 36.90 | 8.55 | 11.50 | 14.96 | 12-499 | 100 | | | | | | | | | | | | | | | | | | | | |
| brdbag_s_soya_dunno_med | 48.05 | 10.88 | 14.60 | 20.07 | 12-499 | 50 | 12-503 | 50 | | | | | | | | | | | | | | | | | | |
| brdbag_s_soya_dunno_thick | 48.05 | 10.88 | 14.60 | 20.07 | 12-499 | 50 | 12-503 | 50 | | | | | | | | | | | | | | | | | | |
| brdbag_s_soya_dunno_thin | 48.05 | 10.88 | 14.60 | 20.07 | 12-499 | 50 | 12-503 | 50 | | | | | | | | | | | | | | | | | | |
| brdbag_s_soya_fat_med | 59.20 | 13.21 | 17.70 | 25.17 | 12-503 | 100 | | | | | | | | | | | | | | | | | | | | |
| brdbag_s_soya_fat_thick | 59.20 | 13.21 | 17.70 | 25.17 | 12-503 | 100 | | | | | | | | | | | | | | | | | | | | |
| brdbag_s_soya_fat_thin | 59.20 | 13.21 | 17.70 | 25.17 | 12-503 | 100 | | | | | | | | | | | | | | | | | | | | |
| brdbag_s_soya_lowfat_med | 36.90 | 8.55 | 11.50 | 14.96 | 12-499 | 100 | | | | | | | | | | | | | | | | | | | | |
| brdbag_s_soya_lowfat_thick | 36.90 | 8.55 | 11.50 | 14.96 | 12-499 | 100 | | | | | | | | | | | | | | | | | | | | |
| brdbag_s_soya_lowfat_thin | 36.90 | 8.55 | 11.50 | 14.96 | 12-499 | 100 | | | | | | | | | | | | | | | | | | | | |
| brdbag_s_soya_vlowfat_med | 36.90 | 8.55 | 11.50 | 14.96 | 12-499 | 100 | | | | | | | | | | | | | | | | | | | | |
| brdbag_s_soya_vlowfat_thick | 36.90 | 8.55 | 11.50 | 14.96 | 12-499 | 100 | | | | | | | | | | | | | | | | | | | | |
| brdbag_s_soya_vlowfat_thin | 36.90 | 8.55 | 11.50 | 14.96 | 12-499 | 100 | | | | | | | | | | | | | | | | | | | | |
| brdcri_s_butt_fat_med | 79.10 | 34.15 | 29.54 | 10.00 | 17-655 | 100 | | | | | | | | | | | | | | | | | | | | |
| brdcri_s_butt_fat_thick | 79.10 | 34.15 | 29.54 | 10.00 | 17-655 | 100 | | | | | | | | | | | | | | | | | | | | |
| brdcri_s_butt_fat_thin | 79.10 | 34.15 | 29.54 | 10.00 | 17-655 | 100 | | | | | | | | | | | | | | | | | | | | |
| brdcri_s_butt_lowfat_med | 60.20 | 25.70 | 22.66 | 7.75 | 17-656 | 100 | | | | | | | | | | | | | | | | | | | | |
| brdcri_s_butt_lowfat_thick | 60.20 | 25.70 | 22.66 | 7.75 | 17-656 | 100 | | | | | | | | | | | | | | | | | | | | |
| brdcri_s_butt_lowfat_thin | 60.20 | 25.70 | 22.66 | 7.75 | 17-656 | 100 | | | | | | | | | | | | | | | | | | | | |
| brdcri_s_butter_dunno_med | 80.65 | 43.12 | 25.23 | 6.42 | 17-655 | 50 | 17-685 | 25 | 17-661 | 25 | | | | | | | | | | | | | | | | |
| brdcri_s_butter_dunno_thick | 80.65 | 43.12 | 25.23 | 6.42 | 17-655 | 50 | 17-685 | 25 | 17-661 | 25 | | | | | | | | | | | | | | | | |
| brdcri_s_butter_dunno_thin | 80.65 | 43.12 | 25.23 | 6.42 | 17-655 | 50 | 17-685 | 25 | 17-661 | 25 | | | | | | | | | | | | | | | | |
| brdcri_s_butter_fat_med | 82.20 | 52.09 | 20.91 | 2.83 | 17-685 | 50 | 17-661 | 50 | | | | | | | | | | | | | | | | | | |
| brdcri_s_butter_fat_thick | 82.20 | 52.09 | 20.91 | 2.83 | 17-685 | 50 | 17-661 | 50 | | | | | | | | | | | | | | | | | | |
| brdcri_s_butter_fat_thin | 82.20 | 52.09 | 20.91 | 2.83 | 17-685 | 50 | 17-661 | 50 | | | | | | | | | | | | | | | | | | |
| brdcri_s_butter_lowfat_med | 60.20 | 25.70 | 22.66 | 7.75 | 17-656 | 100 | | | | | | | | | | | | | | | | | | | | |
| brdcri_s_butter_lowfat_thick | 60.20 | 25.70 | 22.66 | 7.75 | 17-656 | 100 | | | | | | | | | | | | | | | | | | | | |
| brdcri_s_butter_lowfat_thin | 60.20 | 25.70 | 22.66 | 7.75 | 17-656 | 100 | | | | | | | | | | | | | | | | | | | | |
| brdcri_s_dairy_chol_med | 39.00 | 9.76 | 17.39 | 9.82 | 12-497 | 100 | | | | | | | | | | | | | | | | | | | | |
| brdcri_s_dairy_chol_thick | 39.00 | 9.76 | 17.39 | 9.82 | 12-497 | 100 | | | | | | | | | | | | | | | | | | | | |
| brdcri_s_dairy_chol_thin | 39.00 | 9.76 | 17.39 | 9.82 | 12-497 | 100 | | | | | | | | | | | | | | | | | | | | |
| brdcri_s_dairy_dunno_med | 49.80 | 12.69 | 23.67 | 10.86 | 12-497 | 50 | 12-504 | 50 | | | | | | | | | | | | | | | | | | |
| brdcri_s_dairy_dunno_thick | 49.80 | 12.69 | 23.67 | 10.86 | 12-497 | 50 | 12-504 | 50 | | | | | | | | | | | | | | | | | | |
| brdcri_s_dairy_dunno_thin | 49.80 | 12.69 | 23.67 | 10.86 | 12-497 | 50 | 12-504 | 50 | | | | | | | | | | | | | | | | | | |
| brdcri_s_dairy_fat_med | 60.60 | 15.61 | 29.95 | 11.90 | 12-504 | 100 | | | | | | | | | | | | | | | | | | | | |
| brdcri_s_dairy_fat_thick | 60.60 | 15.61 | 29.95 | 11.90 | 12-504 | 100 | | | | | | | | | | | | | | | | | | | | |
| brdcri_s_dairy_fat_thin | 60.60 | 15.61 | 29.95 | 11.90 | 12-504 | 100 | | | | | | | | | | | | | | | | | | | | |
| brdcri_s_dairy_lowfat_med | 39.00 | 9.76 | 17.39 | 9.82 | 12-497 | 100 | | | | | | | | | | | | | | | | | | | | |
| brdcri_s_dairy_lowfat_thick | 39.00 | 9.76 | 17.39 | 9.82 | 12-497 | 100 | | | | | | | | | | | | | | | | | | | | |
| brdcri_s_dairy_lowfat_thin | 39.00 | 9.76 | 17.39 | 9.82 | 12-497 | 100 | | | | | | | | | | | | | | | | | | | | |
| brdcri_s_dairy_vlowfat_med | 39.00 | 9.76 | 17.39 | 9.82 | 12-497 | 100 | | | | | | | | | | | | | | | | | | | | |
| brdcri_s_dairy_vlowfat_thick | 39.00 | 9.76 | 17.39 | 9.82 | 12-497 | 100 | | | | | | | | | | | | | | | | | | | | |
| brdcri_s_dairy_vlowfat_thin | 39.00 | 9.76 | 17.39 | 9.82 | 12-497 | 100 | | | | | | | | | | | | | | | | | | | | |
| brdcri_s_dunno_chol_med | 37.95 | 9.16 | 14.45 | 12.39 | 12-497 | 50 | 12-499 | 50 | | | | | | | | | | | | | | | | | | |
| brdcri_s_dunno_chol_thick | 37.95 | 9.16 | 14.45 | 12.39 | 12-497 | 50 | 12-499 | 50 | | | | | | | | | | | | | | | | | | |
| brdcri_s_dunno_chol_thin | 37.95 | 9.16 | 14.45 | 12.39 | 12-497 | 50 | 12-499 | 50 | | | | | | | | | | | | | | | | | | |
| brdcri_s_dunno_dunno_med | 48.93 | 11.78 | 19.14 | 15.46 | 12-497 | 25 | 12-499 | 25 | 12-503 | 25 | 12-504 | 25 | | | | | | | | | | | | | | |

**Table S1.** Individual FA and major nutrient calculation in the individual FA dataset (MCW + USDA). USDA food codes are highlighted in red.

| Food item | Grams per 100g of food item[1] | | | | Codes for foods from MCW and USDA and the fraction (%) assigned to each code[2] | | | | | | | | | | | | | | | | | | | | | |
|---|---|---|---|---|---|---|---|---|---|---|---|---|---|---|---|---|---|---|---|---|---|---|---|---|---|---|
| | Total fat | Total SFAs | Total MUFAs | Total PUFAs | Code 1 | Fraction 1 | Code 2 | Fraction 2 | Code 3 | Fraction 3 | Code 4 | Fraction 4 | Code 5 | Fraction 5 | Code 6 | Fraction 6 | Code 7 | Fraction 7 | Code 8 | Fraction 8 | Code 9 | Fraction 9 | Code 10 | Fraction 10 | Code 11 | Fraction 11 |
| brdcri_s_dunno_dunno_thick | 48.93 | 11.78 | 19.14 | 15.46 | 12-497 | 25 | 12-499 | 25 | 12-503 | 25 | 12-504 | 25 | | | | | | | | | | | | | | |
| brdcri_s_dunno_dunno_thin | 48.93 | 11.78 | 19.14 | 15.46 | 12-497 | 25 | 12-499 | 25 | 12-503 | 25 | 12-504 | 25 | | | | | | | | | | | | | | |
| brdcri_s_dunno_fat_med | 59.90 | 14.41 | 23.83 | 18.54 | 12-503 | 50 | 12-504 | 50 | | | | | | | | | | | | | | | | | | |
| brdcri_s_dunno_fat_thick | 59.90 | 14.41 | 23.83 | 18.54 | 12-503 | 50 | 12-504 | 50 | | | | | | | | | | | | | | | | | | |
| brdcri_s_dunno_fat_thin | 59.90 | 14.41 | 23.83 | 18.54 | 12-503 | 50 | 12-504 | 50 | | | | | | | | | | | | | | | | | | |
| brdcri_s_dunno_lowfat_med | 37.95 | 9.16 | 14.45 | 12.39 | 12-497 | 50 | 12-499 | 50 | | | | | | | | | | | | | | | | | | |
| brdcri_s_dunno_lowfat_thick | 37.95 | 9.16 | 14.45 | 12.39 | 12-497 | 50 | 12-499 | 50 | | | | | | | | | | | | | | | | | | |
| brdcri_s_dunno_lowfat_thin | 37.95 | 9.16 | 14.45 | 12.39 | 12-497 | 50 | 12-499 | 50 | | | | | | | | | | | | | | | | | | |
| brdcri_s_dunno_vlowfat_med | 37.95 | 9.16 | 14.45 | 12.39 | 12-497 | 50 | 12-499 | 50 | | | | | | | | | | | | | | | | | | |
| brdcri_s_dunno_vlowfat_thick | 37.95 | 9.16 | 14.45 | 12.39 | 12-497 | 50 | 12-499 | 50 | | | | | | | | | | | | | | | | | | |
| brdcri_s_dunno_vlowfat_thin | 37.95 | 9.16 | 14.45 | 12.39 | 12-497 | 50 | 12-499 | 50 | | | | | | | | | | | | | | | | | | |
| brdcri_s_hardmarg_med | 76.40 | 26.41 | 34.00 | 12.21 | 12-500 | 100 | | | | | | | | | | | | | | | | | | | | |
| brdcri_s_hardmarg_thick | 76.40 | 26.41 | 34.00 | 12.21 | 12-500 | 100 | | | | | | | | | | | | | | | | | | | | |
| brdcri_s_hardmarg_thin | 76.40 | 26.41 | 34.00 | 12.21 | 12-500 | 100 | | | | | | | | | | | | | | | | | | | | |
| brdcri_s_olive_chol_med | 38.90 | 8.90 | 21.68 | 6.36 | 12-498 | 100 | | | | | | | | | | | | | | | | | | | | |
| brdcri_s_olive_chol_thick | 38.90 | 8.90 | 21.68 | 6.36 | 12-498 | 100 | | | | | | | | | | | | | | | | | | | | |
| brdcri_s_olive_chol_thin | 38.90 | 8.90 | 21.68 | 6.36 | 12-498 | 100 | | | | | | | | | | | | | | | | | | | | |
| brdcri_s_olive_dunno_med | 49.00 | 11.06 | 26.53 | 8.95 | 12-498 | 50 | 12-505 | 50 | | | | | | | | | | | | | | | | | | |
| brdcri_s_olive_dunno_thick | 49.00 | 11.06 | 26.53 | 8.95 | 12-498 | 50 | 12-505 | 50 | | | | | | | | | | | | | | | | | | |
| brdcri_s_olive_dunno_thin | 49.00 | 11.06 | 26.53 | 8.95 | 12-498 | 50 | 12-505 | 50 | | | | | | | | | | | | | | | | | | |
| brdcri_s_olive_fat_med | 59.10 | 13.22 | 31.37 | 11.53 | 12-505 | 100 | | | | | | | | | | | | | | | | | | | | |
| brdcri_s_olive_fat_thick | 59.10 | 13.22 | 31.37 | 11.53 | 12-505 | 100 | | | | | | | | | | | | | | | | | | | | |
| brdcri_s_olive_fat_thin | 59.10 | 13.22 | 31.37 | 11.53 | 12-505 | 100 | | | | | | | | | | | | | | | | | | | | |
| brdcri_s_olive_lowfat_med | 38.90 | 8.90 | 21.68 | 6.36 | 12-498 | 100 | | | | | | | | | | | | | | | | | | | | |
| brdcri_s_olive_lowfat_thick | 38.90 | 8.90 | 21.68 | 6.36 | 12-498 | 100 | | | | | | | | | | | | | | | | | | | | |
| brdcri_s_olive_lowfat_thin | 38.90 | 8.90 | 21.68 | 6.36 | 12-498 | 100 | | | | | | | | | | | | | | | | | | | | |
| brdcri_s_olive_vlowfat_med | 38.90 | 8.90 | 21.68 | 6.36 | 12-498 | 100 | | | | | | | | | | | | | | | | | | | | |
| brdcri_s_olive_vlowfat_thick | 38.90 | 8.90 | 21.68 | 6.36 | 12-498 | 100 | | | | | | | | | | | | | | | | | | | | |
| brdcri_s_olive_vlowfat_thin | 38.90 | 8.90 | 21.68 | 6.36 | 12-498 | 100 | | | | | | | | | | | | | | | | | | | | |
| brdcri_s_other_med | 68.67 | 33.71 | 21.43 | 9.30 | 81204000 | 18 | 17-685 | 16 | 17-661 | 16 | 12-499 | 12.5 | 12-503 | 12.5 | 12-497 | 12.5 | 12-504 | 12.5 | | | | | | | | |
| brdcri_s_other_thick | 68.67 | 33.71 | 21.43 | 9.30 | 81204000 | 18 | 17-685 | 16 | 17-661 | 16 | 12-499 | 12.5 | 12-503 | 12.5 | 12-497 | 12.5 | 12-504 | 12.5 | | | | | | | | |
| brdcri_s_other_thin | 68.67 | 33.71 | 21.43 | 9.30 | 81204000 | 18 | 17-685 | 16 | 17-661 | 16 | 12-499 | 12.5 | 12-503 | 12.5 | 12-497 | 12.5 | 12-504 | 12.5 | | | | | | | | |
| brdcri_s_poly_chol_med | 36.90 | 8.55 | 11.50 | 14.96 | 12-499 | 100 | | | | | | | | | | | | | | | | | | | | |
| brdcri_s_poly_chol_thick | 36.90 | 8.55 | 11.50 | 14.96 | 12-499 | 100 | | | | | | | | | | | | | | | | | | | | |
| brdcri_s_poly_chol_thin | 36.90 | 8.55 | 11.50 | 14.96 | 12-499 | 100 | | | | | | | | | | | | | | | | | | | | |
| brdcri_s_poly_dunno_med | 48.05 | 10.88 | 14.60 | 20.07 | 12-499 | 50 | 12-503 | 50 | | | | | | | | | | | | | | | | | | |
| brdcri_s_poly_dunno_thick | 48.05 | 10.88 | 14.60 | 20.07 | 12-499 | 50 | 12-503 | 50 | | | | | | | | | | | | | | | | | | |
| brdcri_s_poly_dunno_thin | 48.05 | 10.88 | 14.60 | 20.07 | 12-499 | 50 | 12-503 | 50 | | | | | | | | | | | | | | | | | | |
| brdcri_s_poly_fat_med | 59.20 | 13.21 | 17.70 | 25.17 | 12-503 | 100 | | | | | | | | | | | | | | | | | | | | |
| brdcri_s_poly_fat_thick | 59.20 | 13.21 | 17.70 | 25.17 | 12-503 | 100 | | | | | | | | | | | | | | | | | | | | |
| brdcri_s_poly_fat_thin | 59.20 | 13.21 | 17.70 | 25.17 | 12-503 | 100 | | | | | | | | | | | | | | | | | | | | |
| brdcri_s_poly_lowfat_med | 36.90 | 8.55 | 11.50 | 14.96 | 12-499 | 100 | | | | | | | | | | | | | | | | | | | | |
| brdcri_s_poly_lowfat_thick | 36.90 | 8.55 | 11.50 | 14.96 | 12-499 | 100 | | | | | | | | | | | | | | | | | | | | |
| brdcri_s_poly_lowfat_thin | 36.90 | 8.55 | 11.50 | 14.96 | 12-499 | 100 | | | | | | | | | | | | | | | | | | | | |
| brdcri_s_poly_vlowfat_med | 36.90 | 8.55 | 11.50 | 14.96 | 12-499 | 100 | | | | | | | | | | | | | | | | | | | | |
| brdcri_s_poly_vlowfat_thick | 36.90 | 8.55 | 11.50 | 14.96 | 12-499 | 100 | | | | | | | | | | | | | | | | | | | | |
| brdcri_s_poly_vlowfat_thin | 36.90 | 8.55 | 11.50 | 14.96 | 12-499 | 100 | | | | | | | | | | | | | | | | | | | | |
| brdcri_s_soya_chol_med | 36.90 | 8.55 | 11.50 | 14.96 | 12-499 | 100 | | | | | | | | | | | | | | | | | | | | |
| brdcri_s_soya_chol_thick | 36.90 | 8.55 | 11.50 | 14.96 | 12-499 | 100 | | | | | | | | | | | | | | | | | | | | |
| brdcri_s_soya_chol_thin | 36.90 | 8.55 | 11.50 | 14.96 | 12-499 | 100 | | | | | | | | | | | | | | | | | | | | |
| brdcri_s_soya_dunno_med | 48.05 | 10.88 | 14.60 | 20.07 | 12-499 | 50 | 12-503 | 50 | | | | | | | | | | | | | | | | | | |
| brdcri_s_soya_dunno_thick | 48.05 | 10.88 | 14.60 | 20.07 | 12-499 | 50 | 12-503 | 50 | | | | | | | | | | | | | | | | | | |
| brdcri_s_soya_dunno_thin | 48.05 | 10.88 | 14.60 | 20.07 | 12-499 | 50 | 12-503 | 50 | | | | | | | | | | | | | | | | | | |
| brdcri_s_soya_fat_med | 59.20 | 13.21 | 17.70 | 25.17 | 12-503 | 100 | | | | | | | | | | | | | | | | | | | | |
| brdcri_s_soya_fat_thick | 59.20 | 13.21 | 17.70 | 25.17 | 12-503 | 100 | | | | | | | | | | | | | | | | | | | | |
| brdcri_s_soya_fat_thin | 59.20 | 13.21 | 17.70 | 25.17 | 12-503 | 100 | | | | | | | | | | | | | | | | | | | | |
| brdcri_s_soya_lowfat_med | 36.90 | 8.55 | 11.50 | 14.96 | 12-499 | 100 | | | | | | | | | | | | | | | | | | | | |
| brdcri_s_soya_lowfat_thick | 36.90 | 8.55 | 11.50 | 14.96 | 12-499 | 100 | | | | | | | | | | | | | | | | | | | | |
| brdcri_s_soya_lowfat_thin | 36.90 | 8.55 | 11.50 | 14.96 | 12-499 | 100 | | | | | | | | | | | | | | | | | | | | |
| brdcri_s_soya_vlowfat_med | 36.90 | 8.55 | 11.50 | 14.96 | 12-499 | 100 | | | | | | | | | | | | | | | | | | | | |
| brdcri_s_soya_vlowfat_thick | 36.90 | 8.55 | 11.50 | 14.96 | 12-499 | 100 | | | | | | | | | | | | | | | | | | | | |
| brdcri_s_soya_vlowfat_thin | 36.90 | 8.55 | 11.50 | 14.96 | 12-499 | 100 | | | | | | | | | | | | | | | | | | | | |
| brdlbp_s_butter_dunno_med | 80.65 | 43.12 | 25.23 | 6.42 | 17-655 | 50 | 17-685 | 25 | 17-661 | 25 | | | | | | | | | | | | | | | | |
| brdlbp_s_butter_dunno_thick | 80.65 | 43.12 | 25.23 | 6.42 | 17-655 | 50 | 17-685 | 25 | 17-661 | 25 | | | | | | | | | | | | | | | | |
| brdlbp_s_butter_dunno_thin | 80.65 | 43.12 | 25.23 | 6.42 | 17-655 | 50 | 17-685 | 25 | 17-661 | 25 | | | | | | | | | | | | | | | | |
| brdlbp_s_butter_fat_med | 82.20 | 52.09 | 20.91 | 2.83 | 17-685 | 50 | 17-661 | 50 | | | | | | | | | | | | | | | | | | |
| brdlbp_s_butter_fat_thick | 82.20 | 52.09 | 20.91 | 2.83 | 17-685 | 50 | 17-661 | 50 | | | | | | | | | | | | | | | | | | |
| brdlbp_s_butter_fat_thin | 82.20 | 52.09 | 20.91 | 2.83 | 17-685 | 50 | 17-661 | 50 | | | | | | | | | | | | | | | | | | |
| brdlbp_s_butter_lowfat_med | 60.20 | 25.70 | 22.66 | 7.75 | 17-656 | 100 | | | | | | | | | | | | | | | | | | | | |
| brdlbp_s_butter_lowfat_thick | 60.20 | 25.70 | 22.66 | 7.75 | 17-656 | 100 | | | | | | | | | | | | | | | | | | | | |
| brdlbp_s_butter_lowfat_thin | 60.20 | 25.70 | 22.66 | 7.75 | 17-656 | 100 | | | | | | | | | | | | | | | | | | | | |
| brdlbp_s_buttspr_fat_med | 79.10 | 34.15 | 29.54 | 10.00 | 17-655 | 100 | | | | | | | | | | | | | | | | | | | | |
| brdlbp_s_buttspr_fat_thick | 79.10 | 34.15 | 29.54 | 10.00 | 17-655 | 100 | | | | | | | | | | | | | | | | | | | | |
| brdlbp_s_buttspr_fat_thin | 79.10 | 34.15 | 29.54 | 10.00 | 17-655 | 100 | | | | | | | | | | | | | | | | | | | | |
| brdlbp_s_buttspr_lowfat_med | 60.20 | 25.70 | 22.66 | 7.75 | 17-656 | 100 | | | | | | | | | | | | | | | | | | | | |

**Table S1.** Individual FA and major nutrient calculation in the individual FA dataset (MCW + USDA). USDA food codes are highlighted in red.

| Food item | Grams per 100g of food item[1] | | | | Codes for foods from MCW and USDA and the fraction (%) assigned to each code[2] | | | | | | | | | | | | | | | | | | | | | |
|---|---|---|---|---|---|---|---|---|---|---|---|---|---|---|---|---|---|---|---|---|---|---|---|---|---|---|
| | Total fat | Total SFAs | Total MUFAs | Total PUFAs | Code 1 | Fraction 1 | Code 2 | Fraction 2 | Code 3 | Fraction 3 | Code 4 | Fraction 4 | Code 5 | Fraction 5 | Code 6 | Fraction 6 | Code 7 | Fraction 7 | Code 8 | Fraction 8 | Code 9 | Fraction 9 | Code 10 | Fraction 10 | Code 11 | Fraction 11 |
| brdlbp_s_buttspr_lowfat_thick | 60.20 | 25.70 | 22.66 | 7.75 | 17-656 | 100 | | | | | | | | | | | | | | | | | | | | |
| brdlbp_s_buttspr_lowfat_thin | 60.20 | 25.70 | 22.66 | 7.75 | 17-656 | 100 | | | | | | | | | | | | | | | | | | | | |
| brdlbp_s_dairy_chol_med | 39.00 | 9.76 | 17.39 | 9.82 | 12-497 | 100 | | | | | | | | | | | | | | | | | | | | |
| brdlbp_s_dairy_chol_thick | 39.00 | 9.76 | 17.39 | 9.82 | 12-497 | 100 | | | | | | | | | | | | | | | | | | | | |
| brdlbp_s_dairy_chol_thin | 39.00 | 9.76 | 17.39 | 9.82 | 12-497 | 100 | | | | | | | | | | | | | | | | | | | | |
| brdlbp_s_dairy_dunno_med | 49.80 | 12.69 | 23.67 | 10.86 | 12-497 | 50 | 12-504 | 50 | | | | | | | | | | | | | | | | | | |
| brdlbp_s_dairy_dunno_thick | 49.80 | 12.69 | 23.67 | 10.86 | 12-497 | 50 | 12-504 | 50 | | | | | | | | | | | | | | | | | | |
| brdlbp_s_dairy_dunno_thin | 49.80 | 12.69 | 23.67 | 10.86 | 12-497 | 50 | 12-504 | 50 | | | | | | | | | | | | | | | | | | |
| brdlbp_s_dairy_fat_med | 60.60 | 15.61 | 29.95 | 11.90 | 12-504 | 100 | | | | | | | | | | | | | | | | | | | | |
| brdlbp_s_dairy_fat_thick | 60.60 | 15.61 | 29.95 | 11.90 | 12-504 | 100 | | | | | | | | | | | | | | | | | | | | |
| brdlbp_s_dairy_fat_thin | 60.60 | 15.61 | 29.95 | 11.90 | 12-504 | 100 | | | | | | | | | | | | | | | | | | | | |
| brdlbp_s_dairy_lowfat_med | 39.00 | 9.76 | 17.39 | 9.82 | 12-497 | 100 | | | | | | | | | | | | | | | | | | | | |
| brdlbp_s_dairy_lowfat_thick | 39.00 | 9.76 | 17.39 | 9.82 | 12-497 | 100 | | | | | | | | | | | | | | | | | | | | |
| brdlbp_s_dairy_lowfat_thin | 39.00 | 9.76 | 17.39 | 9.82 | 12-497 | 100 | | | | | | | | | | | | | | | | | | | | |
| brdlbp_s_dairy_vlowfat_med | 39.00 | 9.76 | 17.39 | 9.82 | 12-497 | 100 | | | | | | | | | | | | | | | | | | | | |
| brdlbp_s_dairy_vlowfat_thick | 39.00 | 9.76 | 17.39 | 9.82 | 12-497 | 100 | | | | | | | | | | | | | | | | | | | | |
| brdlbp_s_dairy_vlowfat_thin | 39.00 | 9.76 | 17.39 | 9.82 | 12-497 | 100 | | | | | | | | | | | | | | | | | | | | |
| brdlbp_s_dunno_chol_med | 37.95 | 9.16 | 14.45 | 12.39 | 12-497 | 50 | 12-499 | 50 | | | | | | | | | | | | | | | | | | |
| brdlbp_s_dunno_chol_thick | 37.95 | 9.16 | 14.45 | 12.39 | 12-497 | 50 | 12-499 | 50 | | | | | | | | | | | | | | | | | | |
| brdlbp_s_dunno_chol_thin | 37.95 | 9.16 | 14.45 | 12.39 | 12-497 | 50 | 12-499 | 50 | | | | | | | | | | | | | | | | | | |
| brdlbp_s_dunno_dunno_med | 48.93 | 11.78 | 19.14 | 15.46 | 12-497 | 25 | 12-499 | 25 | 12-503 | 25 | 12-504 | 25 | | | | | | | | | | | | | | |
| brdlbp_s_dunno_dunno_thick | 48.93 | 11.78 | 19.14 | 15.46 | 12-497 | 25 | 12-499 | 25 | 12-503 | 25 | 12-504 | 25 | | | | | | | | | | | | | | |
| brdlbp_s_dunno_dunno_thin | 48.93 | 11.78 | 19.14 | 15.46 | 12-497 | 25 | 12-499 | 25 | 12-503 | 25 | 12-504 | 25 | | | | | | | | | | | | | | |
| brdlbp_s_dunno_fat_med | 59.90 | 14.41 | 23.83 | 18.54 | 12-503 | 50 | 12-504 | 50 | | | | | | | | | | | | | | | | | | |
| brdlbp_s_dunno_fat_thick | 59.90 | 14.41 | 23.83 | 18.54 | 12-503 | 50 | 12-504 | 50 | | | | | | | | | | | | | | | | | | |
| brdlbp_s_dunno_fat_thin | 59.90 | 14.41 | 23.83 | 18.54 | 12-503 | 50 | 12-504 | 50 | | | | | | | | | | | | | | | | | | |
| brdlbp_s_dunno_lowfat_med | 37.95 | 9.16 | 14.45 | 12.39 | 12-497 | 50 | 12-499 | 50 | | | | | | | | | | | | | | | | | | |
| brdlbp_s_dunno_lowfat_thick | 37.95 | 9.16 | 14.45 | 12.39 | 12-497 | 50 | 12-499 | 50 | | | | | | | | | | | | | | | | | | |
| brdlbp_s_dunno_lowfat_thin | 37.95 | 9.16 | 14.45 | 12.39 | 12-497 | 50 | 12-499 | 50 | | | | | | | | | | | | | | | | | | |
| brdlbp_s_dunno_vlowfat_med | 37.95 | 9.16 | 14.45 | 12.39 | 12-497 | 50 | 12-499 | 50 | | | | | | | | | | | | | | | | | | |
| brdlbp_s_dunno_vlowfat_thick | 37.95 | 9.16 | 14.45 | 12.39 | 12-497 | 50 | 12-499 | 50 | | | | | | | | | | | | | | | | | | |
| brdlbp_s_dunno_vlowfat_thin | 37.95 | 9.16 | 14.45 | 12.39 | 12-497 | 50 | 12-499 | 50 | | | | | | | | | | | | | | | | | | |
| brdlbp_s_hardmarg_med | 76.40 | 26.41 | 34.00 | 12.21 | 12-500 | 100 | | | | | | | | | | | | | | | | | | | | |
| brdlbp_s_hardmarg_thick | 76.40 | 26.41 | 34.00 | 12.21 | 12-500 | 100 | | | | | | | | | | | | | | | | | | | | |
| brdlbp_s_hardmarg_thin | 76.40 | 26.41 | 34.00 | 12.21 | 12-500 | 100 | | | | | | | | | | | | | | | | | | | | |
| brdlbp_s_olive_chol_med | 38.90 | 8.90 | 21.68 | 6.36 | 12-498 | 100 | | | | | | | | | | | | | | | | | | | | |
| brdlbp_s_olive_chol_thick | 38.90 | 8.90 | 21.68 | 6.36 | 12-498 | 100 | | | | | | | | | | | | | | | | | | | | |
| brdlbp_s_olive_chol_thin | 38.90 | 8.90 | 21.68 | 6.36 | 12-498 | 100 | | | | | | | | | | | | | | | | | | | | |
| brdlbp_s_olive_dunno_med | 49.00 | 11.06 | 26.53 | 8.95 | 12-498 | 50 | 12-505 | 50 | | | | | | | | | | | | | | | | | | |
| brdlbp_s_olive_dunno_thick | 49.00 | 11.06 | 26.53 | 8.95 | 12-498 | 50 | 12-505 | 50 | | | | | | | | | | | | | | | | | | |
| brdlbp_s_olive_dunno_thin | 49.00 | 11.06 | 26.53 | 8.95 | 12-498 | 50 | 12-505 | 50 | | | | | | | | | | | | | | | | | | |
| brdlbp_s_olive_fat_med | 59.10 | 13.22 | 31.37 | 11.53 | 12-505 | 100 | | | | | | | | | | | | | | | | | | | | |
| brdlbp_s_olive_fat_thick | 59.10 | 13.22 | 31.37 | 11.53 | 12-505 | 100 | | | | | | | | | | | | | | | | | | | | |
| brdlbp_s_olive_fat_thin | 59.10 | 13.22 | 31.37 | 11.53 | 12-505 | 100 | | | | | | | | | | | | | | | | | | | | |
| brdlbp_s_olive_lowfat_med | 38.90 | 8.90 | 21.68 | 6.36 | 12-498 | 100 | | | | | | | | | | | | | | | | | | | | |
| brdlbp_s_olive_lowfat_thick | 38.90 | 8.90 | 21.68 | 6.36 | 12-498 | 100 | | | | | | | | | | | | | | | | | | | | |
| brdlbp_s_olive_lowfat_thin | 38.90 | 8.90 | 21.68 | 6.36 | 12-498 | 100 | | | | | | | | | | | | | | | | | | | | |
| brdlbp_s_olive_vlowfat_med | 38.90 | 8.90 | 21.68 | 6.36 | 12-498 | 100 | | | | | | | | | | | | | | | | | | | | |
| brdlbp_s_olive_vlowfat_thick | 38.90 | 8.90 | 21.68 | 6.36 | 12-498 | 100 | | | | | | | | | | | | | | | | | | | | |
| brdlbp_s_olive_vlowfat_thin | 38.90 | 8.90 | 21.68 | 6.36 | 12-498 | 100 | | | | | | | | | | | | | | | | | | | | |
| brdlbp_s_other_med | 68.67 | 33.71 | 21.43 | 9.30 | 81204000 | 18 | 17-685 | 16 | 17-661 | 16 | 12-499 | 12.5 | 12-503 | 12.5 | 12-497 | 12.5 | 12-504 | 12.5 | | | | | | | | |
| brdlbp_s_other_thick | 68.67 | 33.71 | 21.43 | 9.30 | 81204000 | 18 | 17-685 | 16 | 17-661 | 16 | 12-499 | 12.5 | 12-503 | 12.5 | 12-497 | 12.5 | 12-504 | 12.5 | | | | | | | | |
| brdlbp_s_other_thin | 68.67 | 33.71 | 21.43 | 9.30 | 81204000 | 18 | 17-685 | 16 | 17-661 | 16 | 12-499 | 12.5 | 12-503 | 12.5 | 12-497 | 12.5 | 12-504 | 12.5 | | | | | | | | |
| brdlbp_s_poly_chol_med | 36.90 | 8.55 | 11.50 | 14.96 | 12-499 | 100 | | | | | | | | | | | | | | | | | | | | |
| brdlbp_s_poly_chol_thick | 36.90 | 8.55 | 11.50 | 14.96 | 12-499 | 100 | | | | | | | | | | | | | | | | | | | | |
| brdlbp_s_poly_chol_thin | 36.90 | 8.55 | 11.50 | 14.96 | 12-499 | 100 | | | | | | | | | | | | | | | | | | | | |
| brdlbp_s_poly_dunno_med | 48.05 | 10.88 | 14.60 | 20.07 | 12-499 | 50 | 12-503 | 50 | | | | | | | | | | | | | | | | | | |
| brdlbp_s_poly_dunno_thick | 48.05 | 10.88 | 14.60 | 20.07 | 12-499 | 50 | 12-503 | 50 | | | | | | | | | | | | | | | | | | |
| brdlbp_s_poly_dunno_thin | 48.05 | 10.88 | 14.60 | 20.07 | 12-499 | 50 | 12-503 | 50 | | | | | | | | | | | | | | | | | | |
| brdlbp_s_poly_fat_med | 59.20 | 13.21 | 17.70 | 25.17 | 12-503 | 100 | | | | | | | | | | | | | | | | | | | | |
| brdlbp_s_poly_fat_thick | 59.20 | 13.21 | 17.70 | 25.17 | 12-503 | 100 | | | | | | | | | | | | | | | | | | | | |
| brdlbp_s_poly_fat_thin | 59.20 | 13.21 | 17.70 | 25.17 | 12-503 | 100 | | | | | | | | | | | | | | | | | | | | |
| brdlbp_s_poly_lowfat_med | 36.90 | 8.55 | 11.50 | 14.96 | 12-499 | 100 | | | | | | | | | | | | | | | | | | | | |
| brdlbp_s_poly_lowfat_thick | 36.90 | 8.55 | 11.50 | 14.96 | 12-499 | 100 | | | | | | | | | | | | | | | | | | | | |
| brdlbp_s_poly_lowfat_thin | 36.90 | 8.55 | 11.50 | 14.96 | 12-499 | 100 | | | | | | | | | | | | | | | | | | | | |
| brdlbp_s_poly_vlowfat_med | 36.90 | 8.55 | 11.50 | 14.96 | 12-499 | 100 | | | | | | | | | | | | | | | | | | | | |
| brdlbp_s_poly_vlowfat_thick | 36.90 | 8.55 | 11.50 | 14.96 | 12-499 | 100 | | | | | | | | | | | | | | | | | | | | |
| brdlbp_s_poly_vlowfat_thin | 36.90 | 8.55 | 11.50 | 14.96 | 12-499 | 100 | | | | | | | | | | | | | | | | | | | | |
| brdlbp_s_soya_chol_med | 36.90 | 8.55 | 11.50 | 14.96 | 12-499 | 100 | | | | | | | | | | | | | | | | | | | | |
| brdlbp_s_soya_chol_thick | 36.90 | 8.55 | 11.50 | 14.96 | 12-499 | 100 | | | | | | | | | | | | | | | | | | | | |
| brdlbp_s_soya_chol_thin | 36.90 | 8.55 | 11.50 | 14.96 | 12-499 | 100 | | | | | | | | | | | | | | | | | | | | |
| brdlbp_s_soya_dunno_med | 48.05 | 10.88 | 14.60 | 20.07 | 12-499 | 50 | 12-503 | 50 | | | | | | | | | | | | | | | | | | |
| brdlbp_s_soya_dunno_thick | 48.05 | 10.88 | 14.60 | 20.07 | 12-499 | 50 | 12-503 | 50 | | | | | | | | | | | | | | | | | | |
| brdlbp_s_soya_dunno_thin | 48.05 | 10.88 | 14.60 | 20.07 | 12-499 | 50 | 12-503 | 50 | | | | | | | | | | | | | | | | | | |
| brdlbp_s_soya_fat_med | 59.20 | 13.21 | 17.70 | 25.17 | 12-503 | 100 | | | | | | | | | | | | | | | | | | | | |

**Table S1.** Individual FA and major nutrient calculation in the individual FA dataset (MCW + USDA). USDA food codes are highlighted in red.

| Food item | Grams per 100g of food item[1] | | | | Codes for foods from MCW and USDA and the fraction (%) assigned to each code[2] | | | | | | | | | | | | | | | | | | | | | |
|---|---|---|---|---|---|---|---|---|---|---|---|---|---|---|---|---|---|---|---|---|---|---|---|---|---|---|
| | Total fat | Total SFAs | Total MUFAs | Total PUFAs | Code 1 | Fraction 1 | Code 2 | Fraction 2 | Code 3 | Fraction 3 | Code 4 | Fraction 4 | Code 5 | Fraction 5 | Code 6 | Fraction 6 | Code 7 | Fraction 7 | Code 8 | Fraction 8 | Code 9 | Fraction 9 | Code 10 | Fraction 10 | Code 11 | Fraction 11 |
| brdlbp_s_soya_fat_thick | 59.20 | 13.21 | 17.70 | 25.17 | 12-503 | 100 | | | | | | | | | | | | | | | | | | | | |
| brdlbp_s_soya_fat_thin | 59.20 | 13.21 | 17.70 | 25.17 | 12-503 | 100 | | | | | | | | | | | | | | | | | | | | |
| brdlbp_s_soya_lowfat_med | 36.90 | 8.55 | 11.50 | 14.96 | 12-499 | 100 | | | | | | | | | | | | | | | | | | | | |
| brdlbp_s_soya_lowfat_thick | 36.90 | 8.55 | 11.50 | 14.96 | 12-499 | 100 | | | | | | | | | | | | | | | | | | | | |
| brdlbp_s_soya_lowfat_thin | 36.90 | 8.55 | 11.50 | 14.96 | 12-499 | 100 | | | | | | | | | | | | | | | | | | | | |
| brdlbp_s_soya_vlowfat_med | 36.90 | 8.55 | 11.50 | 14.96 | 12-499 | 100 | | | | | | | | | | | | | | | | | | | | |
| brdlbp_s_soya_vlowfat_thick | 36.90 | 8.55 | 11.50 | 14.96 | 12-499 | 100 | | | | | | | | | | | | | | | | | | | | |
| brdlbp_s_soya_vlowfat_thin | 36.90 | 8.55 | 11.50 | 14.96 | 12-499 | 100 | | | | | | | | | | | | | | | | | | | | |
| brdoth_s_butter_dunno_med | 80.65 | 43.12 | 25.23 | 6.42 | 17-655 | 50 | 17-685 | 25 | 17-661 | 25 | | | | | | | | | | | | | | | | |
| brdoth_s_butter_dunno_thick | 80.65 | 43.12 | 25.23 | 6.42 | 17-655 | 50 | 17-685 | 25 | 17-661 | 25 | | | | | | | | | | | | | | | | |
| brdoth_s_butter_dunno_thin | 80.65 | 43.12 | 25.23 | 6.42 | 17-655 | 50 | 17-685 | 25 | 17-661 | 25 | | | | | | | | | | | | | | | | |
| brdoth_s_butter_fat_med | 82.20 | 52.09 | 20.91 | 2.83 | 17-685 | 50 | 17-661 | 50 | | | | | | | | | | | | | | | | | | |
| brdoth_s_butter_fat_thick | 82.20 | 52.09 | 20.91 | 2.83 | 17-685 | 50 | 17-661 | 50 | | | | | | | | | | | | | | | | | | |
| brdoth_s_butter_fat_thin | 82.20 | 52.09 | 20.91 | 2.83 | 17-685 | 50 | 17-661 | 50 | | | | | | | | | | | | | | | | | | |
| brdoth_s_butter_lowfat_med | 60.20 | 25.70 | 22.66 | 7.75 | 17-656 | 100 | | | | | | | | | | | | | | | | | | | | |
| brdoth_s_butter_lowfat_thick | 60.20 | 25.70 | 22.66 | 7.75 | 17-656 | 100 | | | | | | | | | | | | | | | | | | | | |
| brdoth_s_butter_lowfat_thin | 60.20 | 25.70 | 22.66 | 7.75 | 17-656 | 100 | | | | | | | | | | | | | | | | | | | | |
| brdoth_s_buttspr_fat_med | 79.10 | 34.15 | 29.54 | 10.00 | 17-655 | 100 | | | | | | | | | | | | | | | | | | | | |
| brdoth_s_buttspr_fat_thick | 79.10 | 34.15 | 29.54 | 10.00 | 17-655 | 100 | | | | | | | | | | | | | | | | | | | | |
| brdoth_s_buttspr_fat_thin | 79.10 | 34.15 | 29.54 | 10.00 | 17-655 | 100 | | | | | | | | | | | | | | | | | | | | |
| brdoth_s_buttspr_lowfat_med | 60.20 | 25.70 | 22.66 | 7.75 | 17-656 | 100 | | | | | | | | | | | | | | | | | | | | |
| brdoth_s_buttspr_lowfat_thick | 60.20 | 25.70 | 22.66 | 7.75 | 17-656 | 100 | | | | | | | | | | | | | | | | | | | | |
| brdoth_s_buttspr_lowfat_thin | 60.20 | 25.70 | 22.66 | 7.75 | 17-656 | 100 | | | | | | | | | | | | | | | | | | | | |
| brdoth_s_dairy_chol_med | 39.00 | 9.76 | 17.39 | 9.82 | 12-497 | 100 | | | | | | | | | | | | | | | | | | | | |
| brdoth_s_dairy_chol_thick | 39.00 | 9.76 | 17.39 | 9.82 | 12-497 | 100 | | | | | | | | | | | | | | | | | | | | |
| brdoth_s_dairy_chol_thin | 39.00 | 9.76 | 17.39 | 9.82 | 12-497 | 100 | | | | | | | | | | | | | | | | | | | | |
| brdoth_s_dairy_dunno_med | 49.80 | 12.69 | 23.67 | 10.86 | 12-497 | 50 | 12-504 | 50 | | | | | | | | | | | | | | | | | | |
| brdoth_s_dairy_dunno_thick | 49.80 | 12.69 | 23.67 | 10.86 | 12-497 | 50 | 12-504 | 50 | | | | | | | | | | | | | | | | | | |
| brdoth_s_dairy_dunno_thin | 49.80 | 12.69 | 23.67 | 10.86 | 12-497 | 50 | 12-504 | 50 | | | | | | | | | | | | | | | | | | |
| brdoth_s_dairy_fat_med | 60.60 | 15.61 | 29.95 | 11.90 | 12-504 | 100 | | | | | | | | | | | | | | | | | | | | |
| brdoth_s_dairy_fat_thick | 60.60 | 15.61 | 29.95 | 11.90 | 12-504 | 100 | | | | | | | | | | | | | | | | | | | | |
| brdoth_s_dairy_fat_thin | 60.60 | 15.61 | 29.95 | 11.90 | 12-504 | 100 | | | | | | | | | | | | | | | | | | | | |
| brdoth_s_dairy_lowfat_med | 39.00 | 9.76 | 17.39 | 9.82 | 12-497 | 100 | | | | | | | | | | | | | | | | | | | | |
| brdoth_s_dairy_lowfat_thick | 39.00 | 9.76 | 17.39 | 9.82 | 12-497 | 100 | | | | | | | | | | | | | | | | | | | | |
| brdoth_s_dairy_lowfat_thin | 39.00 | 9.76 | 17.39 | 9.82 | 12-497 | 100 | | | | | | | | | | | | | | | | | | | | |
| brdoth_s_dairy_vlowfat_med | 39.00 | 9.76 | 17.39 | 9.82 | 12-497 | 100 | | | | | | | | | | | | | | | | | | | | |
| brdoth_s_dairy_vlowfat_thick | 39.00 | 9.76 | 17.39 | 9.82 | 12-497 | 100 | | | | | | | | | | | | | | | | | | | | |
| brdoth_s_dairy_vlowfat_thin | 39.00 | 9.76 | 17.39 | 9.82 | 12-497 | 100 | | | | | | | | | | | | | | | | | | | | |
| brdoth_s_dunno_chol_med | 37.95 | 9.16 | 14.45 | 12.39 | 12-497 | 50 | 12-499 | 50 | | | | | | | | | | | | | | | | | | |
| brdoth_s_dunno_chol_thick | 37.95 | 9.16 | 14.45 | 12.39 | 12-497 | 50 | 12-499 | 50 | | | | | | | | | | | | | | | | | | |
| brdoth_s_dunno_chol_thin | 37.95 | 9.16 | 14.45 | 12.39 | 12-497 | 50 | 12-499 | 50 | | | | | | | | | | | | | | | | | | |
| brdoth_s_dunno_dunno_med | 48.93 | 11.78 | 19.14 | 15.46 | 12-497 | 25 | 12-499 | 25 | 12-503 | 25 | 12-504 | 25 | | | | | | | | | | | | | | |
| brdoth_s_dunno_dunno_thick | 48.93 | 11.78 | 19.14 | 15.46 | 12-497 | 25 | 12-499 | 25 | 12-503 | 25 | 12-504 | 25 | | | | | | | | | | | | | | |
| brdoth_s_dunno_dunno_thin | 48.93 | 11.78 | 19.14 | 15.46 | 12-497 | 25 | 12-499 | 25 | 12-503 | 25 | 12-504 | 25 | | | | | | | | | | | | | | |
| brdoth_s_dunno_fat_med | 59.90 | 14.41 | 23.83 | 18.54 | 12-503 | 50 | 12-504 | 50 | | | | | | | | | | | | | | | | | | |
| brdoth_s_dunno_fat_thick | 59.90 | 14.41 | 23.83 | 18.54 | 12-503 | 50 | 12-504 | 50 | | | | | | | | | | | | | | | | | | |
| brdoth_s_dunno_fat_thin | 59.90 | 14.41 | 23.83 | 18.54 | 12-503 | 50 | 12-504 | 50 | | | | | | | | | | | | | | | | | | |
| brdoth_s_dunno_lowfat_med | 37.95 | 9.16 | 14.45 | 12.39 | 12-497 | 50 | 12-499 | 50 | | | | | | | | | | | | | | | | | | |
| brdoth_s_dunno_lowfat_thick | 37.95 | 9.16 | 14.45 | 12.39 | 12-497 | 50 | 12-499 | 50 | | | | | | | | | | | | | | | | | | |
| brdoth_s_dunno_lowfat_thin | 37.95 | 9.16 | 14.45 | 12.39 | 12-497 | 50 | 12-499 | 50 | | | | | | | | | | | | | | | | | | |
| brdoth_s_dunno_vlowfat_med | 37.95 | 9.16 | 14.45 | 12.39 | 12-497 | 50 | 12-499 | 50 | | | | | | | | | | | | | | | | | | |
| brdoth_s_dunno_vlowfat_thick | 37.95 | 9.16 | 14.45 | 12.39 | 12-497 | 50 | 12-499 | 50 | | | | | | | | | | | | | | | | | | |
| brdoth_s_dunno_vlowfat_thin | 37.95 | 9.16 | 14.45 | 12.39 | 12-497 | 50 | 12-499 | 50 | | | | | | | | | | | | | | | | | | |
| brdoth_s_hardmarg_med | 76.40 | 26.41 | 34.00 | 12.21 | 12-500 | 100 | | | | | | | | | | | | | | | | | | | | |
| brdoth_s_hardmarg_thick | 76.40 | 26.41 | 34.00 | 12.21 | 12-500 | 100 | | | | | | | | | | | | | | | | | | | | |
| brdoth_s_hardmarg_thin | 76.40 | 26.41 | 34.00 | 12.21 | 12-500 | 100 | | | | | | | | | | | | | | | | | | | | |
| brdoth_s_olive_chol_med | 38.90 | 8.90 | 21.68 | 6.36 | 12-498 | 100 | | | | | | | | | | | | | | | | | | | | |
| brdoth_s_olive_chol_thick | 38.90 | 8.90 | 21.68 | 6.36 | 12-498 | 100 | | | | | | | | | | | | | | | | | | | | |
| brdoth_s_olive_chol_thin | 38.90 | 8.90 | 21.68 | 6.36 | 12-498 | 100 | | | | | | | | | | | | | | | | | | | | |
| brdoth_s_olive_dunno_med | 49.00 | 11.06 | 26.53 | 8.95 | 12-498 | 50 | 12-505 | 50 | | | | | | | | | | | | | | | | | | |
| brdoth_s_olive_dunno_thick | 49.00 | 11.06 | 26.53 | 8.95 | 12-498 | 50 | 12-505 | 50 | | | | | | | | | | | | | | | | | | |
| brdoth_s_olive_dunno_thin | 49.00 | 11.06 | 26.53 | 8.95 | 12-498 | 50 | 12-505 | 50 | | | | | | | | | | | | | | | | | | |
| brdoth_s_olive_fat_med | 59.10 | 13.22 | 31.37 | 11.53 | 12-505 | 100 | | | | | | | | | | | | | | | | | | | | |
| brdoth_s_olive_fat_thick | 59.10 | 13.22 | 31.37 | 11.53 | 12-505 | 100 | | | | | | | | | | | | | | | | | | | | |
| brdoth_s_olive_fat_thin | 59.10 | 13.22 | 31.37 | 11.53 | 12-505 | 100 | | | | | | | | | | | | | | | | | | | | |
| brdoth_s_olive_lowfat_med | 38.90 | 8.90 | 21.68 | 6.36 | 12-498 | 100 | | | | | | | | | | | | | | | | | | | | |
| brdoth_s_olive_lowfat_thick | 38.90 | 8.90 | 21.68 | 6.36 | 12-498 | 100 | | | | | | | | | | | | | | | | | | | | |
| brdoth_s_olive_lowfat_thin | 38.90 | 8.90 | 21.68 | 6.36 | 12-498 | 100 | | | | | | | | | | | | | | | | | | | | |
| brdoth_s_olive_vlowfat_med | 38.90 | 8.90 | 21.68 | 6.36 | 12-498 | 100 | | | | | | | | | | | | | | | | | | | | |
| brdoth_s_olive_vlowfat_thick | 38.90 | 8.90 | 21.68 | 6.36 | 12-498 | 100 | | | | | | | | | | | | | | | | | | | | |
| brdoth_s_olive_vlowfat_thin | 38.90 | 8.90 | 21.68 | 6.36 | 12-498 | 100 | | | | | | | | | | | | | | | | | | | | |
| brdoth_s_other_med | 68.67 | 33.71 | 21.43 | 9.30 | 81204000 | 18 | 17-685 | 16 | 17-661 | 16 | 12-499 | 12.5 | 12-503 | 12.5 | 12-497 | 12.5 | 12-504 | 12.5 | | | | | | | | |
| brdoth_s_other_thick | 68.67 | 33.71 | 21.43 | 9.30 | 81204000 | 18 | 17-685 | 16 | 17-661 | 16 | 12-499 | 12.5 | 12-503 | 12.5 | 12-497 | 12.5 | 12-504 | 12.5 | | | | | | | | |
| brdoth_s_other_thin | 68.67 | 33.71 | 21.43 | 9.30 | 81204000 | 18 | 17-685 | 16 | 17-661 | 16 | 12-499 | 12.5 | 12-503 | 12.5 | 12-497 | 12.5 | 12-504 | 12.5 | | | | | | | | |
| brdoth_s_poly_chol_med | 36.90 | 8.55 | 11.50 | 14.96 | 12-499 | 100 | | | | | | | | | | | | | | | | | | | | |

**Table S1.** Individual FA and major nutrient calculation in the individual FA dataset (MCW + USDA). USDA food codes are highlighted in red.

| Food item | Grams per 100g of food item[1] | | | | Codes for foods from MCW and USDA and the fraction (%) assigned to each code[2] | | | | | | | | | | | | | | | | | | | | | |
|---|---|---|---|---|---|---|---|---|---|---|---|---|---|---|---|---|---|---|---|---|---|---|---|---|---|---|
| | Total fat | Total SFAs | Total MUFAs | Total PUFAs | Code 1 | Fraction 1 | Code 2 | Fraction 2 | Code 3 | Fraction 3 | Code 4 | Fraction 4 | Code 5 | Fraction 5 | Code 6 | Fraction 6 | Code 7 | Fraction 7 | Code 8 | Fraction 8 | Code 9 | Fraction 9 | Code 10 | Fraction 10 | Code 11 | Fraction 11 |
| brdoth_s_poly_chol_thick | 36.90 | 8.55 | 11.50 | 14.96 | 12-499 | 100 | | | | | | | | | | | | | | | | | | | | |
| brdoth_s_poly_chol_thin | 36.90 | 8.55 | 11.50 | 14.96 | 12-499 | 100 | | | | | | | | | | | | | | | | | | | | |
| brdoth_s_poly_dunno_med | 48.05 | 10.88 | 14.60 | 20.07 | 12-499 | 50 | 12-503 | 50 | | | | | | | | | | | | | | | | | | |
| brdoth_s_poly_dunno_thick | 48.05 | 10.88 | 14.60 | 20.07 | 12-499 | 50 | 12-503 | 50 | | | | | | | | | | | | | | | | | | |
| brdoth_s_poly_dunno_thin | 48.05 | 10.88 | 14.60 | 20.07 | 12-499 | 50 | 12-503 | 50 | | | | | | | | | | | | | | | | | | |
| brdoth_s_poly_fat_med | 59.20 | 13.21 | 17.70 | 25.17 | 12-503 | 100 | | | | | | | | | | | | | | | | | | | | |
| brdoth_s_poly_fat_thick | 59.20 | 13.21 | 17.70 | 25.17 | 12-503 | 100 | | | | | | | | | | | | | | | | | | | | |
| brdoth_s_poly_fat_thin | 59.20 | 13.21 | 17.70 | 25.17 | 12-503 | 100 | | | | | | | | | | | | | | | | | | | | |
| brdoth_s_poly_lowfat_med | 36.90 | 8.55 | 11.50 | 14.96 | 12-499 | 100 | | | | | | | | | | | | | | | | | | | | |
| brdoth_s_poly_lowfat_thick | 36.90 | 8.55 | 11.50 | 14.96 | 12-499 | 100 | | | | | | | | | | | | | | | | | | | | |
| brdoth_s_poly_lowfat_thin | 36.90 | 8.55 | 11.50 | 14.96 | 12-499 | 100 | | | | | | | | | | | | | | | | | | | | |
| brdoth_s_poly_vlowfat_med | 36.90 | 8.55 | 11.50 | 14.96 | 12-499 | 100 | | | | | | | | | | | | | | | | | | | | |
| brdoth_s_poly_vlowfat_thick | 36.90 | 8.55 | 11.50 | 14.96 | 12-499 | 100 | | | | | | | | | | | | | | | | | | | | |
| brdoth_s_poly_vlowfat_thin | 36.90 | 8.55 | 11.50 | 14.96 | 12-499 | 100 | | | | | | | | | | | | | | | | | | | | |
| brdoth_s_soya_chol_med | 36.90 | 8.55 | 11.50 | 14.96 | 12-499 | 100 | | | | | | | | | | | | | | | | | | | | |
| brdoth_s_soya_chol_thick | 36.90 | 8.55 | 11.50 | 14.96 | 12-499 | 100 | | | | | | | | | | | | | | | | | | | | |
| brdoth_s_soya_chol_thin | 36.90 | 8.55 | 11.50 | 14.96 | 12-499 | 100 | | | | | | | | | | | | | | | | | | | | |
| brdoth_s_soya_dunno_med | 48.05 | 10.88 | 14.60 | 20.07 | 12-499 | 50 | 12-503 | 50 | | | | | | | | | | | | | | | | | | |
| brdoth_s_soya_dunno_thick | 48.05 | 10.88 | 14.60 | 20.07 | 12-499 | 50 | 12-503 | 50 | | | | | | | | | | | | | | | | | | |
| brdoth_s_soya_dunno_thin | 48.05 | 10.88 | 14.60 | 20.07 | 12-499 | 50 | 12-503 | 50 | | | | | | | | | | | | | | | | | | |
| brdoth_s_soya_fat_med | 59.20 | 13.21 | 17.70 | 25.17 | 12-503 | 100 | | | | | | | | | | | | | | | | | | | | |
| brdoth_s_soya_fat_thick | 59.20 | 13.21 | 17.70 | 25.17 | 12-503 | 100 | | | | | | | | | | | | | | | | | | | | |
| brdoth_s_soya_fat_thin | 59.20 | 13.21 | 17.70 | 25.17 | 12-503 | 100 | | | | | | | | | | | | | | | | | | | | |
| brdoth_s_soya_lowfat_med | 36.90 | 8.55 | 11.50 | 14.96 | 12-499 | 100 | | | | | | | | | | | | | | | | | | | | |
| brdoth_s_soya_lowfat_thick | 36.90 | 8.55 | 11.50 | 14.96 | 12-499 | 100 | | | | | | | | | | | | | | | | | | | | |
| brdoth_s_soya_lowfat_thin | 36.90 | 8.55 | 11.50 | 14.96 | 12-499 | 100 | | | | | | | | | | | | | | | | | | | | |
| brdoth_s_soya_vlowfat_med | 36.90 | 8.55 | 11.50 | 14.96 | 12-499 | 100 | | | | | | | | | | | | | | | | | | | | |
| brdoth_s_soya_vlowfat_thick | 36.90 | 8.55 | 11.50 | 14.96 | 12-499 | 100 | | | | | | | | | | | | | | | | | | | | |
| brdoth_s_soya_vlowfat_thin | 36.90 | 8.55 | 11.50 | 14.96 | 12-499 | 100 | | | | | | | | | | | | | | | | | | | | |
| brdrol_s_butter_dunno_med | 80.65 | 43.12 | 25.23 | 6.42 | 17-655 | 50 | 17-685 | 25 | 17-661 | 25 | | | | | | | | | | | | | | | | |
| brdrol_s_butter_dunno_thick | 80.65 | 43.12 | 25.23 | 6.42 | 17-655 | 50 | 17-685 | 25 | 17-661 | 25 | | | | | | | | | | | | | | | | |
| brdrol_s_butter_dunno_thin | 80.65 | 43.12 | 25.23 | 6.42 | 17-655 | 50 | 17-685 | 25 | 17-661 | 25 | | | | | | | | | | | | | | | | |
| brdrol_s_butter_fat_med | 82.20 | 52.09 | 20.91 | 2.83 | 17-685 | 50 | 17-661 | 50 | | | | | | | | | | | | | | | | | | |
| brdrol_s_butter_fat_thick | 82.20 | 52.09 | 20.91 | 2.83 | 17-685 | 50 | 17-661 | 50 | | | | | | | | | | | | | | | | | | |
| brdrol_s_butter_fat_thin | 82.20 | 52.09 | 20.91 | 2.83 | 17-685 | 50 | 17-661 | 50 | | | | | | | | | | | | | | | | | | |
| brdrol_s_butter_lowfat_med | 60.20 | 25.70 | 22.66 | 7.75 | 17-656 | 100 | | | | | | | | | | | | | | | | | | | | |
| brdrol_s_butter_lowfat_thick | 60.20 | 25.70 | 22.66 | 7.75 | 17-656 | 100 | | | | | | | | | | | | | | | | | | | | |
| brdrol_s_butter_lowfat_thin | 60.20 | 25.70 | 22.66 | 7.75 | 17-656 | 100 | | | | | | | | | | | | | | | | | | | | |
| brdrol_s_buttspr_fat_med | 79.10 | 34.15 | 29.54 | 10.00 | 17-655 | 100 | | | | | | | | | | | | | | | | | | | | |
| brdrol_s_buttspr_fat_thick | 79.10 | 34.15 | 29.54 | 10.00 | 17-655 | 100 | | | | | | | | | | | | | | | | | | | | |
| brdrol_s_buttspr_fat_thin | 79.10 | 34.15 | 29.54 | 10.00 | 17-655 | 100 | | | | | | | | | | | | | | | | | | | | |
| brdrol_s_buttspr_lowfat_med | 60.20 | 25.70 | 22.66 | 7.75 | 17-656 | 100 | | | | | | | | | | | | | | | | | | | | |
| brdrol_s_buttspr_lowfat_thick | 60.20 | 25.70 | 22.66 | 7.75 | 17-656 | 100 | | | | | | | | | | | | | | | | | | | | |
| brdrol_s_buttspr_lowfat_thin | 60.20 | 25.70 | 22.66 | 7.75 | 17-656 | 100 | | | | | | | | | | | | | | | | | | | | |
| brdrol_s_dairy_chol_med | 39.00 | 9.76 | 17.39 | 9.82 | 12-497 | 100 | | | | | | | | | | | | | | | | | | | | |
| brdrol_s_dairy_chol_thick | 39.00 | 9.76 | 17.39 | 9.82 | 12-497 | 100 | | | | | | | | | | | | | | | | | | | | |
| brdrol_s_dairy_chol_thin | 39.00 | 9.76 | 17.39 | 9.82 | 12-497 | 100 | | | | | | | | | | | | | | | | | | | | |
| brdrol_s_dairy_dunno_med | 49.80 | 12.69 | 23.67 | 10.86 | 12-497 | 50 | 12-504 | 50 | | | | | | | | | | | | | | | | | | |
| brdrol_s_dairy_dunno_thick | 49.80 | 12.69 | 23.67 | 10.86 | 12-497 | 50 | 12-504 | 50 | | | | | | | | | | | | | | | | | | |
| brdrol_s_dairy_dunno_thin | 49.80 | 12.69 | 23.67 | 10.86 | 12-497 | 50 | 12-504 | 50 | | | | | | | | | | | | | | | | | | |
| brdrol_s_dairy_fat_med | 60.60 | 15.61 | 29.95 | 11.90 | 12-504 | 100 | | | | | | | | | | | | | | | | | | | | |
| brdrol_s_dairy_fat_thick | 60.60 | 15.61 | 29.95 | 11.90 | 12-504 | 100 | | | | | | | | | | | | | | | | | | | | |
| brdrol_s_dairy_fat_thin | 60.60 | 15.61 | 29.95 | 11.90 | 12-504 | 100 | | | | | | | | | | | | | | | | | | | | |
| brdrol_s_dairy_lowfat_med | 39.00 | 9.76 | 17.39 | 9.82 | 12-497 | 100 | | | | | | | | | | | | | | | | | | | | |
| brdrol_s_dairy_lowfat_thick | 39.00 | 9.76 | 17.39 | 9.82 | 12-497 | 100 | | | | | | | | | | | | | | | | | | | | |
| brdrol_s_dairy_lowfat_thin | 39.00 | 9.76 | 17.39 | 9.82 | 12-497 | 100 | | | | | | | | | | | | | | | | | | | | |
| brdrol_s_dairy_vlowfat_med | 39.00 | 9.76 | 17.39 | 9.82 | 12-497 | 100 | | | | | | | | | | | | | | | | | | | | |
| brdrol_s_dairy_vlowfat_thick | 39.00 | 9.76 | 17.39 | 9.82 | 12-497 | 100 | | | | | | | | | | | | | | | | | | | | |
| brdrol_s_dairy_vlowfat_thin | 39.00 | 9.76 | 17.39 | 9.82 | 12-497 | 100 | | | | | | | | | | | | | | | | | | | | |
| brdrol_s_dunno_chol_med | 37.95 | 9.16 | 14.45 | 12.39 | 12-497 | 50 | 12-499 | 50 | | | | | | | | | | | | | | | | | | |
| brdrol_s_dunno_chol_thick | 37.95 | 9.16 | 14.45 | 12.39 | 12-497 | 50 | 12-499 | 50 | | | | | | | | | | | | | | | | | | |
| brdrol_s_dunno_chol_thin | 37.95 | 9.16 | 14.45 | 12.39 | 12-497 | 50 | 12-499 | 50 | | | | | | | | | | | | | | | | | | |
| brdrol_s_dunno_dunno_med | 48.93 | 11.78 | 19.14 | 15.46 | 12-497 | 25 | 12-499 | 25 | 12-503 | 25 | 12-504 | 25 | | | | | | | | | | | | | | |
| brdrol_s_dunno_dunno_thick | 48.93 | 11.78 | 19.14 | 15.46 | 12-497 | 25 | 12-499 | 25 | 12-503 | 25 | 12-504 | 25 | | | | | | | | | | | | | | |
| brdrol_s_dunno_dunno_thin | 48.93 | 11.78 | 19.14 | 15.46 | 12-497 | 25 | 12-499 | 25 | 12-503 | 25 | 12-504 | 25 | | | | | | | | | | | | | | |
| brdrol_s_dunno_fat_med | 59.90 | 14.41 | 23.83 | 18.54 | 12-503 | 50 | 12-504 | 50 | | | | | | | | | | | | | | | | | | |
| brdrol_s_dunno_fat_thick | 59.90 | 14.41 | 23.83 | 18.54 | 12-503 | 50 | 12-504 | 50 | | | | | | | | | | | | | | | | | | |
| brdrol_s_dunno_fat_thin | 59.90 | 14.41 | 23.83 | 18.54 | 12-503 | 50 | 12-504 | 50 | | | | | | | | | | | | | | | | | | |
| brdrol_s_dunno_lowfat_med | 37.95 | 9.16 | 14.45 | 12.39 | 12-497 | 50 | 12-499 | 50 | | | | | | | | | | | | | | | | | | |
| brdrol_s_dunno_lowfat_thick | 37.95 | 9.16 | 14.45 | 12.39 | 12-497 | 50 | 12-499 | 50 | | | | | | | | | | | | | | | | | | |
| brdrol_s_dunno_lowfat_thin | 37.95 | 9.16 | 14.45 | 12.39 | 12-497 | 50 | 12-499 | 50 | | | | | | | | | | | | | | | | | | |
| brdrol_s_dunno_vlowfat_med | 37.95 | 9.16 | 14.45 | 12.39 | 12-497 | 50 | 12-499 | 50 | | | | | | | | | | | | | | | | | | |
| brdrol_s_dunno_vlowfat_thick | 37.95 | 9.16 | 14.45 | 12.39 | 12-497 | 50 | 12-499 | 50 | | | | | | | | | | | | | | | | | | |
| brdrol_s_dunno_vlowfat_thin | 37.95 | 9.16 | 14.45 | 12.39 | 12-497 | 50 | 12-499 | 50 | | | | | | | | | | | | | | | | | | |
| brdrol_s_hardmarg_med | 76.40 | 26.41 | 34.00 | 12.21 | 12-500 | 100 | | | | | | | | | | | | | | | | | | | | |

**Table S1.** Individual FA and major nutrient calculation in the individual FA dataset (MCW + USDA). USDA food codes are highlighted in red.

| Food item | Grams per 100g of food item[1] | | | | Codes for foods from MCW and USDA and the fraction (%) assigned to each code[2] | | | | | | | | | | | | | | | | | | | | | |
|---|---|---|---|---|---|---|---|---|---|---|---|---|---|---|---|---|---|---|---|---|---|---|---|---|---|---|
| | Total fat | Total SFAs | Total MUFAs | Total PUFAs | Code 1 | Fraction 1 | Code 2 | Fraction 2 | Code 3 | Fraction 3 | Code 4 | Fraction 4 | Code 5 | Fraction 5 | Code 6 | Fraction 6 | Code 7 | Fraction 7 | Code 8 | Fraction 8 | Code 9 | Fraction 9 | Code 10 | Fraction 10 | Code 11 | Fraction 11 |
| brdrol_s_hardmarg_thick | 76.40 | 26.41 | 34.00 | 12.21 | 12-500 | 100 | | | | | | | | | | | | | | | | | | | | |
| brdrol_s_hardmarg_thin | 76.40 | 26.41 | 34.00 | 12.21 | 12-500 | 100 | | | | | | | | | | | | | | | | | | | | |
| brdrol_s_olive_chol_med | 38.90 | 8.90 | 21.68 | 6.36 | 12-498 | 100 | | | | | | | | | | | | | | | | | | | | |
| brdrol_s_olive_chol_thick | 38.90 | 8.90 | 21.68 | 6.36 | 12-498 | 100 | | | | | | | | | | | | | | | | | | | | |
| brdrol_s_olive_chol_thin | 38.90 | 8.90 | 21.68 | 6.36 | 12-498 | 100 | | | | | | | | | | | | | | | | | | | | |
| brdrol_s_olive_dunno_med | 49.00 | 11.06 | 26.53 | 8.95 | 12-498 | 50 | 12-505 | 50 | | | | | | | | | | | | | | | | | | |
| brdrol_s_olive_dunno_thick | 49.00 | 11.06 | 26.53 | 8.95 | 12-498 | 50 | 12-505 | 50 | | | | | | | | | | | | | | | | | | |
| brdrol_s_olive_dunno_thin | 49.00 | 11.06 | 26.53 | 8.95 | 12-498 | 50 | 12-505 | 50 | | | | | | | | | | | | | | | | | | |
| brdrol_s_olive_fat_med | 59.10 | 13.22 | 31.37 | 11.53 | 12-505 | 100 | | | | | | | | | | | | | | | | | | | | |
| brdrol_s_olive_fat_thick | 59.10 | 13.22 | 31.37 | 11.53 | 12-505 | 100 | | | | | | | | | | | | | | | | | | | | |
| brdrol_s_olive_fat_thin | 59.10 | 13.22 | 31.37 | 11.53 | 12-505 | 100 | | | | | | | | | | | | | | | | | | | | |
| brdrol_s_olive_lowfat_med | 38.90 | 8.90 | 21.68 | 6.36 | 12-498 | 100 | | | | | | | | | | | | | | | | | | | | |
| brdrol_s_olive_lowfat_thick | 38.90 | 8.90 | 21.68 | 6.36 | 12-498 | 100 | | | | | | | | | | | | | | | | | | | | |
| brdrol_s_olive_lowfat_thin | 38.90 | 8.90 | 21.68 | 6.36 | 12-498 | 100 | | | | | | | | | | | | | | | | | | | | |
| brdrol_s_olive_vlowfat_med | 38.90 | 8.90 | 21.68 | 6.36 | 12-498 | 100 | | | | | | | | | | | | | | | | | | | | |
| brdrol_s_olive_vlowfat_thick | 38.90 | 8.90 | 21.68 | 6.36 | 12-498 | 100 | | | | | | | | | | | | | | | | | | | | |
| brdrol_s_olive_vlowfat_thin | 38.90 | 8.90 | 21.68 | 6.36 | 12-498 | 100 | | | | | | | | | | | | | | | | | | | | |
| brdrol_s_other_med | 68.67 | 33.71 | 21.43 | 9.30 | 81204000 | 18 | 17-685 | 16 | 17-661 | 16 | 12-499 | 12.5 | 12-503 | 12.5 | 12-497 | 12.5 | 12-504 | 12.5 | | | | | | | | |
| brdrol_s_other_thick | 68.67 | 33.71 | 21.43 | 9.30 | 81204000 | 18 | 17-685 | 16 | 17-661 | 16 | 12-499 | 12.5 | 12-503 | 12.5 | 12-497 | 12.5 | 12-504 | 12.5 | | | | | | | | |
| brdrol_s_other_thin | 68.67 | 33.71 | 21.43 | 9.30 | 81204000 | 18 | 17-685 | 16 | 17-661 | 16 | 12-499 | 12.5 | 12-503 | 12.5 | 12-497 | 12.5 | 12-504 | 12.5 | | | | | | | | |
| brdrol_s_poly_chol_med | 36.90 | 8.55 | 11.50 | 14.96 | 12-499 | 100 | | | | | | | | | | | | | | | | | | | | |
| brdrol_s_poly_chol_thick | 36.90 | 8.55 | 11.50 | 14.96 | 12-499 | 100 | | | | | | | | | | | | | | | | | | | | |
| brdrol_s_poly_chol_thin | 36.90 | 8.55 | 11.50 | 14.96 | 12-499 | 100 | | | | | | | | | | | | | | | | | | | | |
| brdrol_s_poly_dunno_med | 48.05 | 10.88 | 14.60 | 20.07 | 12-499 | 50 | 12-503 | 50 | | | | | | | | | | | | | | | | | | |
| brdrol_s_poly_dunno_thick | 48.05 | 10.88 | 14.60 | 20.07 | 12-499 | 50 | 12-503 | 50 | | | | | | | | | | | | | | | | | | |
| brdrol_s_poly_dunno_thin | 48.05 | 10.88 | 14.60 | 20.07 | 12-499 | 50 | 12-503 | 50 | | | | | | | | | | | | | | | | | | |
| brdrol_s_poly_fat_med | 59.20 | 13.21 | 17.70 | 25.17 | 12-503 | 100 | | | | | | | | | | | | | | | | | | | | |
| brdrol_s_poly_fat_thick | 59.20 | 13.21 | 17.70 | 25.17 | 12-503 | 100 | | | | | | | | | | | | | | | | | | | | |
| brdrol_s_poly_fat_thin | 59.20 | 13.21 | 17.70 | 25.17 | 12-503 | 100 | | | | | | | | | | | | | | | | | | | | |
| brdrol_s_poly_lowfat_med | 36.90 | 8.55 | 11.50 | 14.96 | 12-499 | 100 | | | | | | | | | | | | | | | | | | | | |
| brdrol_s_poly_lowfat_thick | 36.90 | 8.55 | 11.50 | 14.96 | 12-499 | 100 | | | | | | | | | | | | | | | | | | | | |
| brdrol_s_poly_lowfat_thin | 36.90 | 8.55 | 11.50 | 14.96 | 12-499 | 100 | | | | | | | | | | | | | | | | | | | | |
| brdrol_s_poly_vlowfat_med | 36.90 | 8.55 | 11.50 | 14.96 | 12-499 | 100 | | | | | | | | | | | | | | | | | | | | |
| brdrol_s_poly_vlowfat_thick | 36.90 | 8.55 | 11.50 | 14.96 | 12-499 | 100 | | | | | | | | | | | | | | | | | | | | |
| brdrol_s_poly_vlowfat_thin | 36.90 | 8.55 | 11.50 | 14.96 | 12-499 | 100 | | | | | | | | | | | | | | | | | | | | |
| brdrol_s_soya_chol_med | 36.90 | 8.55 | 11.50 | 14.96 | 12-499 | 100 | | | | | | | | | | | | | | | | | | | | |
| brdrol_s_soya_chol_thick | 36.90 | 8.55 | 11.50 | 14.96 | 12-499 | 100 | | | | | | | | | | | | | | | | | | | | |
| brdrol_s_soya_chol_thin | 36.90 | 8.55 | 11.50 | 14.96 | 12-499 | 100 | | | | | | | | | | | | | | | | | | | | |
| brdrol_s_soya_dunno_med | 48.05 | 10.88 | 14.60 | 20.07 | 12-499 | 50 | 12-503 | 50 | | | | | | | | | | | | | | | | | | |
| brdrol_s_soya_dunno_thick | 48.05 | 10.88 | 14.60 | 20.07 | 12-499 | 50 | 12-503 | 50 | | | | | | | | | | | | | | | | | | |
| brdrol_s_soya_dunno_thin | 48.05 | 10.88 | 14.60 | 20.07 | 12-499 | 50 | 12-503 | 50 | | | | | | | | | | | | | | | | | | |
| brdrol_s_soya_fat_med | 59.20 | 13.21 | 17.70 | 25.17 | 12-503 | 100 | | | | | | | | | | | | | | | | | | | | |
| brdrol_s_soya_fat_thick | 59.20 | 13.21 | 17.70 | 25.17 | 12-503 | 100 | | | | | | | | | | | | | | | | | | | | |
| brdrol_s_soya_fat_thin | 59.20 | 13.21 | 17.70 | 25.17 | 12-503 | 100 | | | | | | | | | | | | | | | | | | | | |
| brdrol_s_soya_lowfat_med | 36.90 | 8.55 | 11.50 | 14.96 | 12-499 | 100 | | | | | | | | | | | | | | | | | | | | |
| brdrol_s_soya_lowfat_thick | 36.90 | 8.55 | 11.50 | 14.96 | 12-499 | 100 | | | | | | | | | | | | | | | | | | | | |
| brdrol_s_soya_lowfat_thin | 36.90 | 8.55 | 11.50 | 14.96 | 12-499 | 100 | | | | | | | | | | | | | | | | | | | | |
| brdrol_s_soya_vlowfat_med | 36.90 | 8.55 | 11.50 | 14.96 | 12-499 | 100 | | | | | | | | | | | | | | | | | | | | |
| brdrol_s_soya_vlowfat_thick | 36.90 | 8.55 | 11.50 | 14.96 | 12-499 | 100 | | | | | | | | | | | | | | | | | | | | |
| brdrol_s_soya_vlowfat_thin | 36.90 | 8.55 | 11.50 | 14.96 | 12-499 | 100 | | | | | | | | | | | | | | | | | | | | |
| brdsli_s_butter_dunno_med | 80.65 | 43.12 | 25.23 | 6.42 | 17-655 | 50 | 17-685 | 25 | 17-661 | 25 | | | | | | | | | | | | | | | | |
| brdsli_s_butter_dunno_thick | 80.65 | 43.12 | 25.23 | 6.42 | 17-655 | 50 | 17-685 | 25 | 17-661 | 25 | | | | | | | | | | | | | | | | |
| brdsli_s_butter_dunno_thin | 80.65 | 43.12 | 25.23 | 6.42 | 17-655 | 50 | 17-685 | 25 | 17-661 | 25 | | | | | | | | | | | | | | | | |
| brdsli_s_butter_fat_med | 82.20 | 52.09 | 20.91 | 2.83 | 17-685 | 50 | 17-661 | 50 | | | | | | | | | | | | | | | | | | |
| brdsli_s_butter_fat_thick | 82.20 | 52.09 | 20.91 | 2.83 | 17-685 | 50 | 17-661 | 50 | | | | | | | | | | | | | | | | | | |
| brdsli_s_butter_fat_thin | 82.20 | 52.09 | 20.91 | 2.83 | 17-685 | 50 | 17-661 | 50 | | | | | | | | | | | | | | | | | | |
| brdsli_s_butter_lowfat_med | 60.20 | 25.70 | 22.66 | 7.75 | 17-656 | 100 | | | | | | | | | | | | | | | | | | | | |
| brdsli_s_butter_lowfat_thick | 60.20 | 25.70 | 22.66 | 7.75 | 17-656 | 100 | | | | | | | | | | | | | | | | | | | | |
| brdsli_s_butter_lowfat_thin | 60.20 | 25.70 | 22.66 | 7.75 | 17-656 | 100 | | | | | | | | | | | | | | | | | | | | |
| brdsli_s_buttspr_fat_med | 79.10 | 34.15 | 29.54 | 10.00 | 17-655 | 100 | | | | | | | | | | | | | | | | | | | | |
| brdsli_s_buttspr_fat_thick | 79.10 | 34.15 | 29.54 | 10.00 | 17-655 | 100 | | | | | | | | | | | | | | | | | | | | |
| brdsli_s_buttspr_fat_thin | 79.10 | 34.15 | 29.54 | 10.00 | 17-655 | 100 | | | | | | | | | | | | | | | | | | | | |
| brdsli_s_buttspr_lowfat_med | 60.20 | 25.70 | 22.66 | 7.75 | 17-656 | 100 | | | | | | | | | | | | | | | | | | | | |
| brdsli_s_buttspr_lowfat_thick | 60.20 | 25.70 | 22.66 | 7.75 | 17-656 | 100 | | | | | | | | | | | | | | | | | | | | |
| brdsli_s_buttspr_lowfat_thin | 60.20 | 25.70 | 22.66 | 7.75 | 17-656 | 100 | | | | | | | | | | | | | | | | | | | | |
| brdsli_s_dairy_chol_med | 39.00 | 9.76 | 17.39 | 9.82 | 12-497 | 100 | | | | | | | | | | | | | | | | | | | | |
| brdsli_s_dairy_chol_thick | 39.00 | 9.76 | 17.39 | 9.82 | 12-497 | 100 | | | | | | | | | | | | | | | | | | | | |
| brdsli_s_dairy_chol_thin | 39.00 | 9.76 | 17.39 | 9.82 | 12-497 | 100 | | | | | | | | | | | | | | | | | | | | |
| brdsli_s_dairy_dunno_med | 49.80 | 12.69 | 23.67 | 10.86 | 12-497 | 50 | 12-504 | 50 | | | | | | | | | | | | | | | | | | |
| brdsli_s_dairy_dunno_thick | 49.80 | 12.69 | 23.67 | 10.86 | 12-497 | 50 | 12-504 | 50 | | | | | | | | | | | | | | | | | | |
| brdsli_s_dairy_dunno_thin | 49.80 | 12.69 | 23.67 | 10.86 | 12-497 | 50 | 12-504 | 50 | | | | | | | | | | | | | | | | | | |
| brdsli_s_dairy_fat_med | 60.60 | 15.61 | 29.95 | 11.90 | 12-504 | 100 | | | | | | | | | | | | | | | | | | | | |
| brdsli_s_dairy_fat_thick | 60.60 | 15.61 | 29.95 | 11.90 | 12-504 | 100 | | | | | | | | | | | | | | | | | | | | |
| brdsli_s_dairy_fat_thin | 60.60 | 15.61 | 29.95 | 11.90 | 12-504 | 100 | | | | | | | | | | | | | | | | | | | | |
| brdsli_s_dairy_lowfat_med | 39.00 | 9.76 | 17.39 | 9.82 | 12-497 | 100 | | | | | | | | | | | | | | | | | | | | |

**Table S1.** Individual FA and major nutrient calculation in the individual FA dataset (MCW + USDA). USDA food codes are highlighted in red.

| Food item | Grams per 100g of food item[1] | | | | Codes for foods from MCW and USDA and the fraction (%) assigned to each code[2] | | | | | | | | | | | | | | | | | | | | | |
|---|---|---|---|---|---|---|---|---|---|---|---|---|---|---|---|---|---|---|---|---|---|---|---|---|---|---|
| | Total fat | Total SFAs | Total MUFAs | Total PUFAs | Code 1 | Fraction 1 | Code 2 | Fraction 2 | Code 3 | Fraction 3 | Code 4 | Fraction 4 | Code 5 | Fraction 5 | Code 6 | Fraction 6 | Code 7 | Fraction 7 | Code 8 | Fraction 8 | Code 9 | Fraction 9 | Code 10 | Fraction 10 | Code 11 | Fraction 11 |
| brdsli_s_dairy_lowfat_thick | 39.00 | 9.76 | 17.39 | 9.82 | 12-497 | 100 | | | | | | | | | | | | | | | | | | | | |
| brdsli_s_dairy_lowfat_thin | 39.00 | 9.76 | 17.39 | 9.82 | 12-497 | 100 | | | | | | | | | | | | | | | | | | | | |
| brdsli_s_dairy_vlowfat_med | 39.00 | 9.76 | 17.39 | 9.82 | 12-497 | 100 | | | | | | | | | | | | | | | | | | | | |
| brdsli_s_dairy_vlowfat_thick | 39.00 | 9.76 | 17.39 | 9.82 | 12-497 | 100 | | | | | | | | | | | | | | | | | | | | |
| brdsli_s_dairy_vlowfat_thin | 39.00 | 9.76 | 17.39 | 9.82 | 12-497 | 100 | | | | | | | | | | | | | | | | | | | | |
| brdsli_s_dunno_chol_med | 37.95 | 9.16 | 14.45 | 12.39 | 12-497 | 50 | 12-499 | 50 | | | | | | | | | | | | | | | | | | |
| brdsli_s_dunno_chol_thick | 37.95 | 9.16 | 14.45 | 12.39 | 12-497 | 50 | 12-499 | 50 | | | | | | | | | | | | | | | | | | |
| brdsli_s_dunno_chol_thin | 37.95 | 9.16 | 14.45 | 12.39 | 12-497 | 50 | 12-499 | 50 | | | | | | | | | | | | | | | | | | |
| brdsli_s_dunno_dunno_med | 48.93 | 11.78 | 19.14 | 15.46 | 12-497 | 25 | 12-499 | 25 | 12-503 | 25 | 12-504 | 25 | | | | | | | | | | | | | | |
| brdsli_s_dunno_dunno_thick | 48.93 | 11.78 | 19.14 | 15.46 | 12-497 | 25 | 12-499 | 25 | 12-503 | 25 | 12-504 | 25 | | | | | | | | | | | | | | |
| brdsli_s_dunno_dunno_thin | 48.93 | 11.78 | 19.14 | 15.46 | 12-497 | 25 | 12-499 | 25 | 12-503 | 25 | 12-504 | 25 | | | | | | | | | | | | | | |
| brdsli_s_dunno_fat_med | 59.90 | 14.41 | 23.83 | 18.54 | 12-503 | 50 | 12-504 | 50 | | | | | | | | | | | | | | | | | | |
| brdsli_s_dunno_fat_thick | 59.90 | 14.41 | 23.83 | 18.54 | 12-503 | 50 | 12-504 | 50 | | | | | | | | | | | | | | | | | | |
| brdsli_s_dunno_fat_thin | 59.90 | 14.41 | 23.83 | 18.54 | 12-503 | 50 | 12-504 | 50 | | | | | | | | | | | | | | | | | | |
| brdsli_s_dunno_lowfat_med | 37.95 | 9.16 | 14.45 | 12.39 | 12-497 | 50 | 12-499 | 50 | | | | | | | | | | | | | | | | | | |
| brdsli_s_dunno_lowfat_thick | 37.95 | 9.16 | 14.45 | 12.39 | 12-497 | 50 | 12-499 | 50 | | | | | | | | | | | | | | | | | | |
| brdsli_s_dunno_lowfat_thin | 37.95 | 9.16 | 14.45 | 12.39 | 12-497 | 50 | 12-499 | 50 | | | | | | | | | | | | | | | | | | |
| brdsli_s_dunno_vlowfat_med | 37.95 | 9.16 | 14.45 | 12.39 | 12-497 | 50 | 12-499 | 50 | | | | | | | | | | | | | | | | | | |
| brdsli_s_dunno_vlowfat_thick | 37.95 | 9.16 | 14.45 | 12.39 | 12-497 | 50 | 12-499 | 50 | | | | | | | | | | | | | | | | | | |
| brdsli_s_dunno_vlowfat_thin | 37.95 | 9.16 | 14.45 | 12.39 | 12-497 | 50 | 12-499 | 50 | | | | | | | | | | | | | | | | | | |
| brdsli_s_hardmarg_med | 76.40 | 26.41 | 34.00 | 12.21 | 12-500 | 100 | | | | | | | | | | | | | | | | | | | | |
| brdsli_s_hardmarg_thick | 76.40 | 26.41 | 34.00 | 12.21 | 12-500 | 100 | | | | | | | | | | | | | | | | | | | | |
| brdsli_s_hardmarg_thin | 76.40 | 26.41 | 34.00 | 12.21 | 12-500 | 100 | | | | | | | | | | | | | | | | | | | | |
| brdsli_s_olive_chol_med | 38.90 | 8.90 | 21.68 | 6.36 | 12-498 | 100 | | | | | | | | | | | | | | | | | | | | |
| brdsli_s_olive_chol_thick | 38.90 | 8.90 | 21.68 | 6.36 | 12-498 | 100 | | | | | | | | | | | | | | | | | | | | |
| brdsli_s_olive_chol_thin | 38.90 | 8.90 | 21.68 | 6.36 | 12-498 | 100 | | | | | | | | | | | | | | | | | | | | |
| brdsli_s_olive_dunno_med | 49.00 | 11.06 | 26.53 | 8.95 | 12-498 | 50 | 12-505 | 50 | | | | | | | | | | | | | | | | | | |
| brdsli_s_olive_dunno_thick | 49.00 | 11.06 | 26.53 | 8.95 | 12-498 | 50 | 12-505 | 50 | | | | | | | | | | | | | | | | | | |
| brdsli_s_olive_dunno_thin | 49.00 | 11.06 | 26.53 | 8.95 | 12-498 | 50 | 12-505 | 50 | | | | | | | | | | | | | | | | | | |
| brdsli_s_olive_fat_med | 59.10 | 13.22 | 31.37 | 11.53 | 12-505 | 100 | | | | | | | | | | | | | | | | | | | | |
| brdsli_s_olive_fat_thick | 59.10 | 13.22 | 31.37 | 11.53 | 12-505 | 100 | | | | | | | | | | | | | | | | | | | | |
| brdsli_s_olive_fat_thin | 59.10 | 13.22 | 31.37 | 11.53 | 12-505 | 100 | | | | | | | | | | | | | | | | | | | | |
| brdsli_s_olive_lowfat_med | 38.90 | 8.90 | 21.68 | 6.36 | 12-498 | 100 | | | | | | | | | | | | | | | | | | | | |
| brdsli_s_olive_lowfat_thick | 38.90 | 8.90 | 21.68 | 6.36 | 12-498 | 100 | | | | | | | | | | | | | | | | | | | | |
| brdsli_s_olive_lowfat_thin | 38.90 | 8.90 | 21.68 | 6.36 | 12-498 | 100 | | | | | | | | | | | | | | | | | | | | |
| brdsli_s_olive_vlowfat_med | 38.90 | 8.90 | 21.68 | 6.36 | 12-498 | 100 | | | | | | | | | | | | | | | | | | | | |
| brdsli_s_olive_vlowfat_thick | 38.90 | 8.90 | 21.68 | 6.36 | 12-498 | 100 | | | | | | | | | | | | | | | | | | | | |
| brdsli_s_olive_vlowfat_thin | 38.90 | 8.90 | 21.68 | 6.36 | 12-498 | 100 | | | | | | | | | | | | | | | | | | | | |
| brdsli_s_other_med | 68.67 | 33.71 | 21.43 | 9.30 | 81204000 | 18 | 17-685 | 16 | 17-661 | 16 | 12-499 | 12.5 | 12-503 | 12.5 | 12-497 | 12.5 | 12-504 | 12.5 | | | | | | | | |
| brdsli_s_other_thick | 68.67 | 33.71 | 21.43 | 9.30 | 81204000 | 18 | 17-685 | 16 | 17-661 | 16 | 12-499 | 12.5 | 12-503 | 12.5 | 12-497 | 12.5 | 12-504 | 12.5 | | | | | | | | |
| brdsli_s_other_thin | 68.67 | 33.71 | 21.43 | 9.30 | 81204000 | 18 | 17-685 | 16 | 17-661 | 16 | 12-499 | 12.5 | 12-503 | 12.5 | 12-497 | 12.5 | 12-504 | 12.5 | | | | | | | | |
| brdsli_s_poly_chol_med | 36.90 | 8.55 | 11.50 | 14.96 | 12-499 | 100 | | | | | | | | | | | | | | | | | | | | |
| brdsli_s_poly_chol_thick | 36.90 | 8.55 | 11.50 | 14.96 | 12-499 | 100 | | | | | | | | | | | | | | | | | | | | |
| brdsli_s_poly_chol_thin | 36.90 | 8.55 | 11.50 | 14.96 | 12-499 | 100 | | | | | | | | | | | | | | | | | | | | |
| brdsli_s_poly_dunno_med | 48.05 | 10.88 | 14.60 | 20.07 | 12-499 | 50 | 12-503 | 50 | | | | | | | | | | | | | | | | | | |
| brdsli_s_poly_dunno_thick | 48.05 | 10.88 | 14.60 | 20.07 | 12-499 | 50 | 12-503 | 50 | | | | | | | | | | | | | | | | | | |
| brdsli_s_poly_dunno_thin | 48.05 | 10.88 | 14.60 | 20.07 | 12-499 | 50 | 12-503 | 50 | | | | | | | | | | | | | | | | | | |
| brdsli_s_poly_fat_med | 59.20 | 13.21 | 17.70 | 25.17 | 12-503 | 100 | | | | | | | | | | | | | | | | | | | | |
| brdsli_s_poly_fat_thick | 59.20 | 13.21 | 17.70 | 25.17 | 12-503 | 100 | | | | | | | | | | | | | | | | | | | | |
| brdsli_s_poly_fat_thin | 59.20 | 13.21 | 17.70 | 25.17 | 12-503 | 100 | | | | | | | | | | | | | | | | | | | | |
| brdsli_s_poly_lowfat_med | 36.90 | 8.55 | 11.50 | 14.96 | 12-499 | 100 | | | | | | | | | | | | | | | | | | | | |
| brdsli_s_poly_lowfat_thick | 36.90 | 8.55 | 11.50 | 14.96 | 12-499 | 100 | | | | | | | | | | | | | | | | | | | | |
| brdsli_s_poly_lowfat_thin | 36.90 | 8.55 | 11.50 | 14.96 | 12-499 | 100 | | | | | | | | | | | | | | | | | | | | |
| brdsli_s_poly_vlowfat_med | 36.90 | 8.55 | 11.50 | 14.96 | 12-499 | 100 | | | | | | | | | | | | | | | | | | | | |
| brdsli_s_poly_vlowfat_thick | 36.90 | 8.55 | 11.50 | 14.96 | 12-499 | 100 | | | | | | | | | | | | | | | | | | | | |
| brdsli_s_poly_vlowfat_thin | 36.90 | 8.55 | 11.50 | 14.96 | 12-499 | 100 | | | | | | | | | | | | | | | | | | | | |
| brdsli_s_soya_chol_med | 36.90 | 8.55 | 11.50 | 14.96 | 12-499 | 100 | | | | | | | | | | | | | | | | | | | | |
| brdsli_s_soya_chol_thick | 36.90 | 8.55 | 11.50 | 14.96 | 12-499 | 100 | | | | | | | | | | | | | | | | | | | | |
| brdsli_s_soya_chol_thin | 36.90 | 8.55 | 11.50 | 14.96 | 12-499 | 100 | | | | | | | | | | | | | | | | | | | | |
| brdsli_s_soya_dunno_med | 48.05 | 10.88 | 14.60 | 20.07 | 12-499 | 50 | 12-503 | 50 | | | | | | | | | | | | | | | | | | |
| brdsli_s_soya_dunno_thick | 48.05 | 10.88 | 14.60 | 20.07 | 12-499 | 50 | 12-503 | 50 | | | | | | | | | | | | | | | | | | |
| brdsli_s_soya_dunno_thin | 48.05 | 10.88 | 14.60 | 20.07 | 12-499 | 50 | 12-503 | 50 | | | | | | | | | | | | | | | | | | |
| brdsli_s_soya_fat_med | 59.20 | 13.21 | 17.70 | 25.17 | 12-503 | 100 | | | | | | | | | | | | | | | | | | | | |
| brdsli_s_soya_fat_thick | 59.20 | 13.21 | 17.70 | 25.17 | 12-503 | 100 | | | | | | | | | | | | | | | | | | | | |
| brdsli_s_soya_fat_thin | 59.20 | 13.21 | 17.70 | 25.17 | 12-503 | 100 | | | | | | | | | | | | | | | | | | | | |
| brdsli_s_soya_lowfat_med | 36.90 | 8.55 | 11.50 | 14.96 | 12-499 | 100 | | | | | | | | | | | | | | | | | | | | |
| brdsli_s_soya_lowfat_thick | 36.90 | 8.55 | 11.50 | 14.96 | 12-499 | 100 | | | | | | | | | | | | | | | | | | | | |
| brdsli_s_soya_lowfat_thin | 36.90 | 8.55 | 11.50 | 14.96 | 12-499 | 100 | | | | | | | | | | | | | | | | | | | | |
| brdsli_s_soya_vlowfat_med | 36.90 | 8.55 | 11.50 | 14.96 | 12-499 | 100 | | | | | | | | | | | | | | | | | | | | |
| brdsli_s_soya_vlowfat_thick | 36.90 | 8.55 | 11.50 | 14.96 | 12-499 | 100 | | | | | | | | | | | | | | | | | | | | |
| brdsli_s_soya_vlowfat_thin | 36.90 | 8.55 | 11.50 | 14.96 | 12-499 | 100 | | | | | | | | | | | | | | | | | | | | |
| bread_baguette_gf_nonwhite | 14.79 | 2.40 | 4.28 | 7.40 | 11-972 | 15 | 11-971 | 15 | 11-969 | 15 | 11-981 | 15 | 11-975 | 15 | 14-844 | 8.4 | 14-845 | 8.3 | 13-849 | 8.3 | | | | | | |
| bread_baguette_gf_unanswered | 2.80 | 0.49 | 1.04 | 0.78 | 11-969 | 33.4 | 11-981 | 33.3 | 11-971 | 33.3 | | | | | | | | | | | | | | | | |
| bread_baguette_gf_white | 2.90 | 0.46 | 1.20 | 0.77 | 11-978 | 50 | 11-969 | 50 | | | | | | | | | | | | | | | | | | |
| bread_baguette_mixed | 2.73 | 0.53 | 1.05 | 0.76 | 11-972 | 33.4 | 11-971 | 33.3 | 11-969 | 33.3 | | | | | | | | | | | | | | | | |

**Table S1.** Individual FA and major nutrient calculation in the individual FA dataset (MCW + USDA). USDA food codes are highlighted in red.

| Food item | Grams per 100g of food item[1] | | | | Codes for foods from MCW and USDA and the fraction (%) assigned to each code[2] | | | | | | | | | | | | | | | | | | | | | |
|---|---|---|---|---|---|---|---|---|---|---|---|---|---|---|---|---|---|---|---|---|---|---|---|---|---|---|
| | Total fat | Total SFAs | Total MUFAs | Total PUFAs | Code 1 | Fraction 1 | Code 2 | Fraction 2 | Code 3 | Fraction 3 | Code 4 | Fraction 4 | Code 5 | Fraction 5 | Code 6 | Fraction 6 | Code 7 | Fraction 7 | Code 8 | Fraction 8 | Code 9 | Fraction 9 | Code 10 | Fraction 10 | Code 11 | Fraction 11 |
| bread_baguette_other | 3.35 | 0.56 | 1.49 | 0.85 | 11-975 | 13.4 | 11-981 | 13.3 | 11-969 | 60 | 11-971 | 13.3 | | | | | | | | | | | | | | |
| bread_baguette_seeded | 50.87 | 7.96 | 14.45 | 27.10 | 14-844 | 33.4 | 14-845 | 33.3 | 13-849 | 33.3 | | | | | | | | | | | | | | | | |
| bread_baguette_unanswered | 2.80 | 0.49 | 1.04 | 0.78 | 11-969 | 33.4 | 11-981 | 33.3 | 11-971 | 33.3 | | | | | | | | | | | | | | | | |
| bread_baguette_white | 2.90 | 0.46 | 1.20 | 0.77 | 11-978 | 50 | 11-969 | 50 | | | | | | | | | | | | | | | | | | |
| bread_baguette_wholemeal | 2.50 | 0.46 | 0.59 | 0.82 | 11-981 | 100 | | | | | | | | | | | | | | | | | | | | |
| bread_crisp | 7.91 | 4.08 | 2.09 | 1.11 | 11-822 | 40 | 11-820 | 30 | 11-826 | 30 | | | | | | | | | | | | | | | | |
| bread_crisp_gf | 7.91 | 4.08 | 2.09 | 1.11 | 11-822 | 40 | 11-820 | 30 | 11-826 | 30 | | | | | | | | | | | | | | | | |
| bread_garlic | 16.70 | 8.63 | 5.39 | 1.56 | 11-937 | 100 | | | | | | | | | | | | | | | | | | | | |
| bread_large_bap_gf_nonwhite | 14.60 | 2.41 | 4.05 | 7.39 | 11-972 | 15 | 11-971 | 15 | 11-1006 | 15 | 11-981 | 15 | 11-975 | 15 | 14-844 | 8.4 | 14-845 | 8.3 | 13-849 | 8.3 | | | | | | |
| bread_large_bap_gf_unanswered | 2.37 | 0.50 | 0.54 | 0.76 | 11-1006 | 33.4 | 11-971 | 33.3 | 11-981 | 33.3 | | | | | | | | | | | | | | | | |
| bread_large_bap_gf_white | 2.40 | 0.57 | 0.54 | 0.77 | 11-985 | 50 | 11-1006 | 50 | | | | | | | | | | | | | | | | | | |
| bread_large_bap_mixed | 2.30 | 0.54 | 0.54 | 0.74 | 11-972 | 33.4 | 11-971 | 33.3 | 11-1006 | 33.3 | | | | | | | | | | | | | | | | |
| bread_large_bap_other | 2.57 | 0.59 | 0.58 | 0.83 | 11-975 | 13.4 | 11-981 | 13.3 | 11-1006 | 60 | 11-971 | 13.3 | | | | | | | | | | | | | | |
| bread_large_bap_seeded | 50.87 | 7.96 | 14.45 | 27.10 | 14-844 | 33.4 | 14-845 | 33.3 | 13-849 | 33.3 | | | | | | | | | | | | | | | | |
| bread_large_bap_unanswered | 2.37 | 0.50 | 0.54 | 0.76 | 11-1006 | 33.4 | 11-971 | 33.3 | 11-981 | 33.3 | | | | | | | | | | | | | | | | |
| bread_large_bap_white | 2.40 | 0.57 | 0.54 | 0.77 | 11-985 | 50 | 11-1006 | 50 | | | | | | | | | | | | | | | | | | |
| bread_large_bap_wholemeal | 2.50 | 0.46 | 0.59 | 0.82 | 11-981 | 100 | | | | | | | | | | | | | | | | | | | | |
| bread_naan | 7.30 | 0.97 | 3.05 | 2.37 | 11-973 | 100 | | | | | | | | | | | | | | | | | | | | |
| bread_other | 2.56 | 0.96 | 0.55 | 0.67 | 11-541 | 80 | 11-826 | 10 | 11-462 | 10 | | | | | | | | | | | | | | | | |
| bread_other_gf | 2.56 | 0.96 | 0.55 | 0.67 | 11-541 | 80 | 11-826 | 10 | 11-462 | 10 | | | | | | | | | | | | | | | | |
| bread_roll_gf_nonwhite | 14.60 | 2.41 | 4.05 | 7.39 | 11-972 | 15 | 11-971 | 15 | 11-1006 | 15 | 11-981 | 15 | 11-975 | 15 | 14-844 | 8.4 | 14-845 | 8.3 | 13-849 | 8.3 | | | | | | |
| bread_roll_gf_unanswered | 2.37 | 0.50 | 0.54 | 0.76 | 11-1006 | 33.4 | 11-971 | 33.3 | 11-981 | 33.3 | | | | | | | | | | | | | | | | |
| bread_roll_gf_white | 2.40 | 0.57 | 0.54 | 0.77 | 11-985 | 50 | 11-1006 | 50 | | | | | | | | | | | | | | | | | | |
| bread_roll_mixed | 2.30 | 0.54 | 0.54 | 0.74 | 11-972 | 33.4 | 11-971 | 33.3 | 11-1006 | 33.3 | | | | | | | | | | | | | | | | |
| bread_roll_other | 2.57 | 0.59 | 0.58 | 0.83 | 11-975 | 13.4 | 11-981 | 13.3 | 11-1006 | 60 | 11-971 | 13.3 | | | | | | | | | | | | | | |
| bread_roll_seeded | 50.87 | 7.96 | 14.45 | 27.10 | 14-844 | 33.4 | 14-845 | 33.3 | 13-849 | 33.3 | | | | | | | | | | | | | | | | |
| bread_roll_unanswered | 2.37 | 0.50 | 0.54 | 0.76 | 11-1006 | 33.4 | 11-971 | 33.3 | 11-981 | 33.3 | | | | | | | | | | | | | | | | |
| bread_roll_white | 2.40 | 0.57 | 0.54 | 0.77 | 11-985 | 50 | 11-1006 | 50 | | | | | | | | | | | | | | | | | | |
| bread_roll_wholemeal | 2.50 | 0.46 | 0.59 | 0.82 | 11-981 | 100 | | | | | | | | | | | | | | | | | | | | |
| bread_sliced_gf_nonwhite | 14.45 | 2.36 | 4.01 | 7.34 | 11-972 | 15 | 11-971 | 15 | 11-980 | 15 | 11-975 | 15 | 11-981 | 15 | 14-844 | 8.4 | 14-845 | 8.3 | 13-849 | 8.3 | | | | | | |
| bread_sliced_gf_unanswered | 2.03 | 0.39 | 0.45 | 0.66 | 11-980 | 33.4 | 11-981 | 33.3 | 11-971 | 33.3 | | | | | | | | | | | | | | | | |
| bread_sliced_gf_white | 1.60 | 0.29 | 0.32 | 0.52 | 11-980 | 100 | | | | | | | | | | | | | | | | | | | | |
| bread_sliced_mixed | 1.97 | 0.43 | 0.45 | 0.64 | 11-972 | 33.4 | 11-971 | 33.3 | 11-980 | 33.3 | | | | | | | | | | | | | | | | |
| bread_sliced_other | 2.30 | 0.48 | 0.51 | 0.76 | 11-975 | 25 | 11-981 | 25 | 11-971 | 25 | 11-980 | 25 | | | | | | | | | | | | | | |
| bread_sliced_seeded | 50.87 | 7.96 | 14.45 | 27.10 | 14-844 | 33.4 | 14-845 | 33.3 | 13-849 | 33.3 | | | | | | | | | | | | | | | | |
| bread_sliced_unanswered | 2.03 | 0.39 | 0.45 | 0.66 | 11-980 | 33.4 | 11-981 | 33.3 | 11-971 | 33.3 | | | | | | | | | | | | | | | | |
| bread_sliced_white | 1.60 | 0.29 | 0.32 | 0.52 | 11-980 | 100 | | | | | | | | | | | | | | | | | | | | |
| bread_sliced_wholemeal | 2.50 | 0.46 | 0.59 | 0.82 | 11-981 | 100 | | | | | | | | | | | | | | | | | | | | |
| cake | 21.03 | 7.68 | 8.20 | 3.86 | 11-832 | 20 | 11-814 | 20 | 11-836 | 20 | 11-799 | 6 | 14-318 | 5 | 12-335 | 5 | 17-063 | 1 | 12-324 | 2 | 17-661 | 1 | 11-737 | 20 | | |
| cake_gf | 21.03 | 7.68 | 8.20 | 3.86 | 11-832 | 20 | 11-814 | 20 | 11-836 | 20 | 11-799 | 6 | 14-318 | 5 | 12-335 | 5 | 17-063 | 1 | 12-324 | 2 | 17-661 | 1 | 11-737 | 20 | | |
| cereal_bran | 3.10 | 0.48 | 0.39 | 1.64 | 11-763 | 25 | 11-767 | 75 | | | | | | | | | | | | | | | | | | |
| cereal_bran_driedfruit | 5.80 | 2.65 | 0.88 | 0.64 | 11-779 | 100 | | | | | | | | | | | | | | | | | | | | |
| cereal_muesli | 6.30 | 1.27 | 2.97 | 1.68 | 11-780 | 100 | | | | | | | | | | | | | | | | | | | | |
| cereal_muesli_driedfruit | 5.71 | 1.14 | 2.67 | 1.51 | 11-780 | 90 | 14-087 | 10 | | | | | | | | | | | | | | | | | | |
| cereal_oatcrunch | 20.50 | 4.61 | 10.63 | 4.22 | 11-939 | 100 | | | | | | | | | | | | | | | | | | | | |
| cereal_oatcrunch_driedfruit | 18.49 | 4.15 | 9.57 | 3.80 | 11-939 | 90 | 14-087 | 10 | | | | | | | | | | | | | | | | | | |
| cereal_other | 4.90 | 0.83 | 1.54 | 2.10 | 11-755 | 20 | 11-767 | 40 | 11-792 | 40 | | | | | | | | | | | | | | | | |
| cereal_other_driedfruit | 4.80 | 1.35 | 1.38 | 1.30 | 11-755 | 15 | 11-779 | 32.5 | 11-792 | 32.5 | 14-087 | 20 | | | | | | | | | | | | | | |
| cereal_plain | 1.07 | 0.21 | 0.20 | 0.41 | 11-760 | 33.4 | 11-742 | 16.7 | 11-742 | 16.6 | 11-750 | 16.7 | 11-750 | 16.6 | | | | | | | | | | | | |
| cereal_plain_driedfruit | 0.85 | 0.14 | 0.14 | 0.28 | 11-760 | 22.7 | 11-742 | 11.4 | 11-742 | 11.3 | 11-750 | 11.3 | 11-750 | 11.3 | 14-087 | 32 | | | | | | | | | | |
| cereal_sugar | 0.00 | 0.00 | 0.00 | 0.00 | 17-063 | 33.4 | 17-050 | 33.3 | 17-065 | 33.3 | | | | | | | | | | | | | | | | |
| cereal_sweet | 2.30 | 0.67 | 1.03 | 0.48 | 11-754 | 33.4 | 11-746 | 33.3 | 11-753 | 33.3 | | | | | | | | | | | | | | | | |
| cereal_sweet_driedfruit | 2.11 | 0.61 | 0.92 | 0.43 | 11-754 | 30.06 | 11-746 | 29.97 | 11-753 | 29.97 | 14-087 | 10 | | | | | | | | | | | | | | |
| cereal_wwheat | 2.23 | 0.31 | 0.31 | 0.97 | 11-775 | 33.4 | 11-761 | 33.3 | 11-773 | 33.3 | | | | | | | | | | | | | | | | |
| cereal_wwheat_driedfruit | 1.80 | 0.26 | 0.17 | 0.88 | 11-777 | 50 | 11-773 | 50 | | | | | | | | | | | | | | | | | | |
| cerealbar | 14.12 | 5.91 | 5.69 | 1.80 | 11-819 | 55 | 11-818 | 45 | | | | | | | | | | | | | | | | | | |
| cerealbar_gf | 14.12 | 5.91 | 5.69 | 1.80 | 11-819 | 55 | 11-818 | 45 | | | | | | | | | | | | | | | | | | |
| cheese_blue | 33.88 | 21.65 | 9.04 | 1.35 | 12-354 | 25 | 12-367 | 25 | 12-494 | 50 | | | | | | | | | | | | | | | | |
| cheese_cottage | 3.56 | 1.42 | 0.64 | 0.10 | 14201010 | 60 | 14204010 | 40 | | | | | | | | | | | | | | | | | | |
| cheese_feta | 20.30 | 13.75 | 5.04 | 0.76 | 12-360 | 100 | | | | | | | | | | | | | | | | | | | | |
| cheese_goat | 25.80 | 17.91 | 6.09 | 1.04 | 12-357 | 100 | | | | | | | | | | | | | | | | | | | | |
| cheese_hard | 33.86 | 21.19 | 9.08 | 1.10 | 12-346 | 80 | 12-526 | 20 | | | | | | | | | | | | | | | | | | |
| cheese_hard_lof | 14.10 | 8.85 | 4.13 | 0.41 | 14410120 | 100 | | | | | | | | | | | | | | | | | | | | |
| cheese_mozzarella | 20.30 | 13.75 | 5.04 | 0.76 | 12-360 | 100 | | | | | | | | | | | | | | | | | | | | |
| cheese_other | 26.70 | 18.13 | 6.60 | 1.06 | 12-346 | 20 | 12-496 | 40 | 12-357 | 40 | | | | | | | | | | | | | | | | |
| cheese_soft | 25.75 | 17.99 | 6.34 | 1.03 | 12-492 | 50 | 12-357 | 50 | | | | | | | | | | | | | | | | | | |
| cheese_spread | 23.00 | 14.32 | 6.34 | 0.83 | 12-527 | 100 | | | | | | | | | | | | | | | | | | | | |
| cheese_spread_lof | 8.88 | 5.65 | 2.46 | 0.42 | 14410330 | 100 | | | | | | | | | | | | | | | | | | | | |
| cheesecake | 12.30 | 7.54 | 3.49 | 0.51 | 12-542 | 100 | | | | | | | | | | | | | | | | | | | | |
| cheesecake_gf | 12.30 | 7.54 | 3.49 | 0.51 | 12-542 | 100 | | | | | | | | | | | | | | | | | | | | |
| choc_bar | 25.21 | 11.81 | 10.19 | 1.95 | 17-658 | 34 | 17-670 | 33 | 11-740 | 33 | | | | | | | | | | | | | | | | |
| choc_dark | 28.00 | 16.80 | 9.00 | 1.00 | 17-491 | 100 | | | | | | | | | | | | | | | | | | | | |
| choc_milk | 31.10 | 18.71 | 9.60 | 1.13 | 17-648 | 100 | | | | | | | | | | | | | | | | | | | | |
| choc_sweets | 21.30 | 11.30 | 8.00 | 1.00 | 17-690 | 100 | | | | | | | | | | | | | | | | | | | | |
| choc_white | 32.09 | 19.41 | 9.10 | 1.01 | 91705400 | 100 | | | | | | | | | | | | | | | | | | | | |

**Table S1.** Individual FA and major nutrient calculation in the individual FA dataset (MCW + USDA). USDA food codes are highlighted in red.

| Food item | Grams per 100g of food item[1] | | | | Codes for foods from MCW and USDA and the fraction (%) assigned to each code[2] | | | | | | | | | | | | | | | | | | | | | |
|---|---|---|---|---|---|---|---|---|---|---|---|---|---|---|---|---|---|---|---|---|---|---|---|---|---|---|
| | Total fat | Total SFAs | Total MUFAs | Total PUFAs | Code 1 | Fraction 1 | Code 2 | Fraction 2 | Code 3 | Fraction 3 | Code 4 | Fraction 4 | Code 5 | Fraction 5 | Code 6 | Fraction 6 | Code 7 | Fraction 7 | Code 8 | Fraction 8 | Code 9 | Fraction 9 | Code 10 | Fraction 10 | Code 11 | Fraction 11 |
| chocyog_raisin | 16.55 | 9.78 | 4.77 | 0.60 | 14-393 | 50 | 91705400 | 50 | | | | | | | | | | | | | | | | | | |
| chutney | 0.15 | 0.00 | 0.01 | 0.04 | 17-784 | 25 | 17-718 | 50 | 17-345 | 25 | | | | | | | | | | | | | | | | |
| cof_capp | 0.00 | 0.00 | 0.00 | 0.00 | 17-153 | 100 | | | | | | | | | | | | | | | | | | | | |
| cof_capp_decaf | 0.00 | 0.00 | 0.00 | 0.00 | 17-153 | 100 | | | | | | | | | | | | | | | | | | | | |
| cof_capp_milk_chol | 0.28 | 0.12 | 0.06 | 0.01 | 12-307 | 92.5 | 17-377 | 7.5 | | | | | | | | | | | | | | | | | | |
| cof_capp_milk_dontknow | 1.67 | 1.04 | 0.41 | 0.04 | 12-313 | 55.5 | 12-307 | 18.5 | 12-596 | 18.5 | 17-377 | 7.5 | | | | | | | | | | | | | | |
| cof_capp_milk_goatsheep | 4.39 | 2.75 | 1.11 | 0.23 | 12-329 | 46.25 | 12-328 | 46.25 | 17-377 | 7.5 | | | | | | | | | | | | | | | | |
| cof_capp_milk_other | 1.41 | 0.65 | 0.50 | 0.11 | 12-313 | 24.975 | 11350000 | 37 | 11114330 | 30.525 | 17-377 | 7.5 | | | | | | | | | | | | | | |
| cof_capp_milk_powdered | 0.28 | 0.12 | 0.06 | 0.01 | 12-307 | 92.5 | 17-377 | 7.5 | | | | | | | | | | | | | | | | | | |
| cof_capp_milk_riceoatveg | 1.22 | 0.11 | 0.44 | 0.60 | 12-524 | 46.25 | 11350000 | 46.25 | 17-377 | 7.5 | | | | | | | | | | | | | | | | |
| cof_capp_milk_semi | 1.57 | 0.99 | 0.37 | 0.04 | 12-313 | 92.5 | 17-377 | 7.5 | | | | | | | | | | | | | | | | | | |
| cof_capp_milk_skimmed | 0.28 | 0.12 | 0.06 | 0.01 | 12-307 | 92.5 | 17-377 | 7.5 | | | | | | | | | | | | | | | | | | |
| cof_capp_milk_soya_ca | 1.48 | 0.22 | 0.31 | 1.02 | 12-524 | 92.5 | 17-377 | 7.5 | | | | | | | | | | | | | | | | | | |
| cof_capp_milk_soya_noca | 1.48 | 0.22 | 0.31 | 1.02 | 12-524 | 92.5 | 17-377 | 7.5 | | | | | | | | | | | | | | | | | | |
| cof_capp_milk_whole | 3.33 | 2.12 | 0.89 | 0.08 | 12-596 | 92.5 | 17-377 | 7.5 | | | | | | | | | | | | | | | | | | |
| cof_espresso | 0.00 | 0.00 | 0.00 | 0.00 | 17-153 | 100 | | | | | | | | | | | | | | | | | | | | |
| cof_espresso_decaf | 0.00 | 0.00 | 0.00 | 0.00 | 17-153 | 100 | | | | | | | | | | | | | | | | | | | | |
| cof_filter | 0.00 | 0.00 | 0.00 | 0.00 | 17-153 | 50 | 17-154 | 50 | | | | | | | | | | | | | | | | | | |
| cof_filter_decaf | 0.00 | 0.00 | 0.00 | 0.00 | 17-153 | 50 | 17-154 | 50 | | | | | | | | | | | | | | | | | | |
| cof_instant | 0.00 | 0.00 | 0.00 | 0.00 | 17-158 | 0.9 | 17-377 | 99.1 | | | | | | | | | | | | | | | | | | |
| cof_instant_decaf | 0.00 | 0.00 | 0.00 | 0.00 | 17-158 | 0.9 | 17-377 | 99.1 | | | | | | | | | | | | | | | | | | |
| cof_latte | 0.00 | 0.00 | 0.00 | 0.00 | 17-153 | 100 | | | | | | | | | | | | | | | | | | | | |
| cof_latte_decaf | 0.00 | 0.00 | 0.00 | 0.00 | 17-153 | 100 | | | | | | | | | | | | | | | | | | | | |
| cof_latte_milk_chol | 0.29 | 0.13 | 0.06 | 0.01 | 12-307 | 96.2 | 17-377 | 3.8 | | | | | | | | | | | | | | | | | | |
| cof_latte_milk_dontknow | 1.73 | 1.08 | 0.43 | 0.04 | 12-313 | 57.72 | 12-307 | 19.24 | 12-596 | 19.24 | 17-377 | 3.8 | | | | | | | | | | | | | | |
| cof_latte_milk_goatsheep | 4.57 | 2.86 | 1.16 | 0.24 | 12-329 | 48.1 | 12-328 | 48.1 | 17-377 | 3.8 | | | | | | | | | | | | | | | | |
| cof_latte_milk_other | 1.47 | 0.68 | 0.52 | 0.11 | 12-313 | 25.974 | 11350000 | 38.48 | 11114330 | 31.746 | 17-377 | 3.8 | | | | | | | | | | | | | | |
| cof_latte_milk_powdered | 0.29 | 0.13 | 0.06 | 0.01 | 12-307 | 96.2 | 17-377 | 3.8 | | | | | | | | | | | | | | | | | | |
| cof_latte_milk_riceoatveg | 1.27 | 0.12 | 0.46 | 0.63 | 12-524 | 48.1 | 11350000 | 48.1 | 17-377 | 3.8 | | | | | | | | | | | | | | | | |
| cof_latte_milk_semi | 1.64 | 1.03 | 0.38 | 0.04 | 12-313 | 96.2 | 17-377 | 3.8 | | | | | | | | | | | | | | | | | | |
| cof_latte_milk_skimmed | 0.29 | 0.13 | 0.06 | 0.01 | 12-307 | 96.2 | 17-377 | 3.8 | | | | | | | | | | | | | | | | | | |
| cof_latte_milk_soya_ca | 1.54 | 0.23 | 0.32 | 1.06 | 12-524 | 96.2 | 17-377 | 3.8 | | | | | | | | | | | | | | | | | | |
| cof_latte_milk_soya_noca | 1.54 | 0.23 | 0.32 | 1.06 | 12-524 | 96.2 | 17-377 | 3.8 | | | | | | | | | | | | | | | | | | |
| cof_latte_milk_whole | 3.46 | 2.20 | 0.92 | 0.09 | 12-596 | 96.2 | 17-377 | 3.8 | | | | | | | | | | | | | | | | | | |
| cof_other | 0.00 | 0.00 | 0.00 | 0.00 | 17-158 | 0.45 | 17-377 | 49.55 | 17-153 | 25 | 17-154 | 25 | | | | | | | | | | | | | | |
| cof_other_decaf | 0.00 | 0.00 | 0.00 | 0.00 | 17-158 | 0.45 | 17-377 | 49.55 | 17-153 | 25 | 17-154 | 25 | | | | | | | | | | | | | | |
| cof_sugar | 0.00 | 0.00 | 0.00 | 0.00 | 17-063 | 66.7 | 17-061 | 33.3 | | | | | | | | | | | | | | | | | | |
| cream | 31.84 | 20.68 | 7.69 | 0.98 | 12-332 | 50 | 12-334 | 16.7 | 12-335 | 33.3 | | | | | | | | | | | | | | | | |
| croissant | 19.70 | 9.76 | 5.86 | 1.84 | 11-988 | 100 | | | | | | | | | | | | | | | | | | | | |
| crumble | 26.15 | 16.04 | 6.72 | 1.22 | 11-886 | 38 | 11-792 | 10 | 17-063 | 20 | 17-661 | 29 | 12-346 | 3 | | | | | | | | | | | | |
| danish_pastry | 29.20 | 11.83 | 11.45 | 4.39 | 11-841 | 100 | | | | | | | | | | | | | | | | | | | | |
| dessert_milkbased | 9.25 | 5.51 | 2.70 | 0.41 | 12-398 | 50 | 12-397 | 25 | 12-476 | 25 | | | | | | | | | | | | | | | | |
| dessert_milkpuds | 1.94 | 1.19 | 0.55 | 0.07 | 12-543 | 34 | 12-580 | 33 | 12-397 | 33 | | | | | | | | | | | | | | | | |
| dessert_other | 6.60 | 2.46 | 2.80 | 0.97 | 11-921 | 40 | 14-332 | 22.5 | 14-253 | 22.5 | 12-409 | 15 | | | | | | | | | | | | | | |
| dessert_soya | 5.76 | 3.42 | 1.41 | 0.68 | 12-609 | 34 | 12-509 | 66 | | | | | | | | | | | | | | | | | | |
| double_crust | 37.90 | 14.10 | 16.02 | 5.84 | 11-853 | 100 | | | | | | | | | | | | | | | | | | | | |
| doughnut | 19.40 | 9.55 | 6.53 | 2.25 | 11-850 | 50 | 11-849 | 50 | | | | | | | | | | | | | | | | | | |
| drink_diethotchoc | 0.40 | 0.28 | 0.07 | 0.03 | 11830260 | 2.75 | 17-732 | 2.75 | 17-377 | 94.5 | | | | | | | | | | | | | | | | |
| drink_fizzy | 0.00 | 0.00 | 0.00 | 0.00 | 17-175 | 33.4 | 17-735 | 33.3 | 17-179 | 33.3 | | | | | | | | | | | | | | | | |
| drink_grapefruit | 0.10 | 0.00 | 0.00 | 0.00 | 14-275 | 100 | | | | | | | | | | | | | | | | | | | | |
| drink_hotchoc | 0.91 | 0.53 | 0.28 | 0.05 | 17-498 | 12.2 | 11830260 | 6 | 17-377 | 81.8 | | | | | | | | | | | | | | | | |
| drink_hotchoc_milk_chol | 0.30 | 0.13 | 0.06 | 0.01 | 12-307 | 100 | | | | | | | | | | | | | | | | | | | | |
| drink_hotchoc_milk_dontknow | 1.80 | 1.13 | 0.44 | 0.04 | 12-313 | 60 | 12-307 | 20 | 12-596 | 20 | | | | | | | | | | | | | | | | |
| drink_hotchoc_milk_goatsheep | 4.75 | 2.98 | 1.21 | 0.25 | 12-328 | 50 | 12-329 | 50 | | | | | | | | | | | | | | | | | | |
| drink_hotchoc_milk_other | 1.53 | 0.70 | 0.54 | 0.12 | 12-313 | 27 | 11350000 | 40 | 11114330 | 33 | | | | | | | | | | | | | | | | |
| drink_hotchoc_milk_powdered | 0.30 | 0.13 | 0.06 | 0.01 | 12-307 | 100 | | | | | | | | | | | | | | | | | | | | |
| drink_hotchoc_milk_riceoatveg | 1.32 | 0.12 | 0.48 | 0.65 | 12-524 | 50 | 11350000 | 50 | | | | | | | | | | | | | | | | | | |
| drink_hotchoc_milk_semi | 1.70 | 1.07 | 0.40 | 0.04 | 12-313 | 100 | | | | | | | | | | | | | | | | | | | | |
| drink_hotchoc_milk_skimmed | 0.30 | 0.13 | 0.06 | 0.01 | 12-307 | 100 | | | | | | | | | | | | | | | | | | | | |
| drink_hotchoc_milk_soya_ca | 1.60 | 0.24 | 0.33 | 1.10 | 12-524 | 100 | | | | | | | | | | | | | | | | | | | | |
| drink_hotchoc_milk_soya_noca | 1.60 | 0.24 | 0.33 | 1.10 | 12-524 | 100 | | | | | | | | | | | | | | | | | | | | |
| drink_hotchoc_milk_whole | 3.60 | 2.29 | 0.96 | 0.09 | 12-596 | 100 | | | | | | | | | | | | | | | | | | | | |
| drink_lowcal | 0.00 | 0.00 | 0.00 | 0.00 | 17-505 | 50 | 17-673 | 25 | 17-739 | 5 | 17-377 | 20 | | | | | | | | | | | | | | |
| drink_milkbased | 1.52 | 1.02 | 0.35 | 0.05 | 12-379 | 20 | 12-327 | 40 | 12-326 | 40 | | | | | | | | | | | | | | | | |
| drink_orange | 0.00 | 0.00 | 0.00 | 0.00 | 14-329 | 33.4 | 14-330 | 33.3 | 14-369 | 33.3 | | | | | | | | | | | | | | | | |
| drink_other | 0.71 | 0.45 | 0.17 | 0.02 | 17-377 | 20 | 12-313 | 40 | 11830260 | 0.9 | 17-377 | 9.1 | 14-330 | 10 | 17-741 | 20 | | | | | | | | | | |
| drink_purejuice | 0.02 | 0.00 | 0.00 | 0.00 | 14-331 | 40 | 14-286 | 20 | 13-382 | 20 | 17-744 | 20 | | | | | | | | | | | | | | |
| drink_squash | 0.00 | 0.00 | 0.00 | 0.00 | 17-738 | 5 | 17-377 | 60 | 17-741 | 25 | 17-674 | 5 | 17-200 | 5 | | | | | | | | | | | | |
| drink_water | 0.00 | 0.00 | 0.00 | 0.00 | 17-377 | 100 | | | | | | | | | | | | | | | | | | | | |
| drizzle_oil | 99.90 | 14.30 | 73.00 | 8.20 | 17-038 | 100 | | | | | | | | | | | | | | | | | | | | |
| egg_omelet | 14.09 | 4.68 | 4.55 | 3.01 | 12-940 | 87.5 | 12-313 | 6.25 | 17-685 | 3.75 | 17-045 | 2.5 | | | | | | | | | | | | | | |
| egg_other | 14.93 | 6.44 | 5.53 | 1.73 | 12-940 | 33.4 | 12-936 | 66.6 | | | | | | | | | | | | | | | | | | |
| egg_scotch | 16.00 | 4.30 | 6.77 | 2.80 | 19-518 | 100 | | | | | | | | | | | | | | | | | | | | |
| egg_swich | 19.38 | 3.14 | 9.97 | 4.27 | 12-940 | 85 | 17-654 | 15 | | | | | | | | | | | | | | | | | | |
| egg_whole | 11.96 | 3.02 | 4.37 | 2.47 | 12-940 | 33.4 | 12-943 | 33.3 | 12-944 | 33.3 | | | | | | | | | | | | | | | | |

**Table S1.** Individual FA and major nutrient calculation in the individual FA dataset (MCW + USDA). USDA food codes are highlighted in red.

| Food item | Grams per 100g of food item[1] | | | | Codes for foods from MCW and USDA and the fraction (%) assigned to each code[2] | | | | | | | | | | | | | | | | | | | | | |
|---|---|---|---|---|---|---|---|---|---|---|---|---|---|---|---|---|---|---|---|---|---|---|---|---|---|---|
| | Total fat | Total SFAs | Total MUFAs | Total PUFAs | Code 1 | Fraction 1 | Code 2 | Fraction 2 | Code 3 | Fraction 3 | Code 4 | Fraction 4 | Code 5 | Fraction 5 | Code 6 | Fraction 6 | Code 7 | Fraction 7 | Code 8 | Fraction 8 | Code 9 | Fraction 9 | Code 10 | Fraction 10 | Code 11 | Fraction 11 |
| fish_battered | 11.80 | 1.71 | 6.65 | 2.75 | 16-369 | 100 | | | | | | | | | | | | | | | | | | | | |
| fish_breaded | 9.75 | 1.22 | 5.09 | 2.85 | 16-405 | 16.5 | 16-407 | 16.5 | 16-403 | 34 | 16-370 | 33 | | | | | | | | | | | | | | |
| fish_lobcrab | 2.83 | 0.43 | 0.61 | 0.72 | 16-391 | 25 | 16-392 | 25 | 16-332 | 50 | | | | | | | | | | | | | | | | |
| fish_oily | 13.17 | 2.79 | 5.09 | 3.31 | 16-176 | 10 | 16-397 | 10 | 16-394 | 10 | 16-358 | 30 | 16-422 | 10 | 16-419 | 20 | 16-400 | 10 | | | | | | | | |
| fish_other | 8.91 | 1.81 | 2.65 | 2.86 | 16-412 | 70 | 16-404 | 6 | 16-396 | 12 | 16-402 | 12 | | | | | | | | | | | | | | |
| fish_prawns | 0.90 | 0.15 | 0.19 | 0.22 | 16-384 | 100 | | | | | | | | | | | | | | | | | | | | |
| fish_shell | 1.96 | 0.36 | 0.21 | 0.55 | 16-262 | 30 | 16-390 | 70 | | | | | | | | | | | | | | | | | | |
| fish_tinnedtuna | 3.70 | 0.54 | 0.91 | 2.01 | 16-416 | 50 | 16-417 | 50 | | | | | | | | | | | | | | | | | | |
| fish_white | 0.40 | 0.09 | 0.07 | 0.14 | 16-373 | 50 | 16-376 | 50 | | | | | | | | | | | | | | | | | | |
| fruit_apple | 0.40 | 0.10 | 0.03 | 0.17 | 14-346 | 100 | | | | | | | | | | | | | | | | | | | | |
| fruit_banana | 0.10 | 0.04 | 0.00 | 0.04 | 14-318 | 100 | | | | | | | | | | | | | | | | | | | | |
| fruit_berry | 0.29 | 0.04 | 0.07 | 0.15 | 14-375 | 25 | 14-324 | 25 | 14-053 | 5 | 14-325 | 25 | 14-388 | 20 | | | | | | | | | | | | |
| fruit_cherry | 0.40 | 0.08 | 0.09 | 0.10 | 14-382 | 100 | | | | | | | | | | | | | | | | | | | | |
| fruit_dried | 0.90 | 0.14 | 0.30 | 0.27 | 14-393 | 62.5 | 14-392 | 12.5 | 14-395 | 12.5 | 14-016 | 12.5 | | | | | | | | | | | | | | |
| fruit_grapefruit | 0.35 | 0.05 | 0.05 | 0.08 | 14-384 | 70 | 14-107 | 30 | | | | | | | | | | | | | | | | | | |
| fruit_grapes | 0.15 | 0.06 | 0.01 | 0.06 | 14-323 | 50 | 14-322 | 50 | | | | | | | | | | | | | | | | | | |
| fruit_mango | 0.38 | 0.09 | 0.14 | 0.07 | 63129010 | 100 | | | | | | | | | | | | | | | | | | | | |
| fruit_melon | 0.17 | 0.03 | 0.03 | 0.03 | 14-339 | 33.4 | 14-159 | 33.3 | 14-296 | 33.3 | | | | | | | | | | | | | | | | |
| fruit_mixed | 0.11 | 0.00 | 0.00 | 0.00 | 14-097 | 35 | 14-336 | 30 | 14-333 | 35 | | | | | | | | | | | | | | | | |
| fruit_orange | 0.20 | 0.05 | 0.05 | 0.07 | 14-327 | 100 | | | | | | | | | | | | | | | | | | | | |
| fruit_other | 0.63 | 0.06 | 0.09 | 0.34 | 14-371 | 60 | 63133010 | 10 | 14-226 | 10 | 14-025 | 10 | 14-091 | 10 | | | | | | | | | | | | |
| fruit_peach | 0.20 | 0.01 | 0.04 | 0.06 | 14-299 | 50 | 14-380 | 50 | | | | | | | | | | | | | | | | | | |
| fruit_pear | 0.10 | 0.01 | 0.03 | 0.03 | 14-361 | 100 | | | | | | | | | | | | | | | | | | | | |
| fruit_pineapple | 0.07 | 0.00 | 0.04 | 0.04 | 14-376 | 70 | 14-211 | 30 | | | | | | | | | | | | | | | | | | |
| fruit_plum | 0.10 | 0.00 | 0.00 | 0.00 | 14-220 | 100 | | | | | | | | | | | | | | | | | | | | |
| fruit_prunes | 0.30 | 0.06 | 0.04 | 0.12 | 14-391 | 100 | | | | | | | | | | | | | | | | | | | | |
| fruit_satsuma | 0.20 | 0.04 | 0.06 | 0.07 | 14-326 | 100 | | | | | | | | | | | | | | | | | | | | |
| fruit_stewed | 0.17 | 0.02 | 0.01 | 0.04 | 14-320 | 33.4 | 14-253 | 33.3 | 14-314 | 33.3 | | | | | | | | | | | | | | | | |
| fruitcake | 12.10 | 4.64 | 4.72 | 1.88 | 11-829 | 100 | | | | | | | | | | | | | | | | | | | | |
| fruitcake_gf | 12.10 | 4.64 | 4.72 | 1.88 | 11-829 | 100 | | | | | | | | | | | | | | | | | | | | |
| grains_couscous | 0.95 | 0.14 | 0.12 | 0.38 | 11-901 | 45 | 17-377 | 55 | | | | | | | | | | | | | | | | | | |
| grains_other | 0.83 | 0.14 | 0.22 | 0.30 | 11-904 | 10.5 | 17-377 | 24.5 | 11-869 | 55 | 11-877 | 10 | | | | | | | | | | | | | | |
| guacamole | 12.47 | 1.81 | 8.33 | 1.55 | 63105010 | 85 | 14-277 | 5 | 13-517 | 10 | | | | | | | | | | | | | | | | |
| hummus | 20.95 | 3.47 | 8.64 | 8.47 | 41302120 | 58 | 14-844 | 29 | 14-277 | 9 | 17-041 | 1.5 | 17-038 | 1.5 | 75111500 | 1 | | | | | | | | | | |
| icecream | 12.33 | 8.13 | 3.06 | 0.45 | 12-508 | 33.4 | 12-509 | 33.3 | 12-510 | 33.3 | | | | | | | | | | | | | | | | |
| indian_snack | 12.18 | 2.07 | 4.07 | 5.37 | 13-489 | 8.75 | 41302120 | 20 | 75214010 | 8.75 | 17-045 | 5 | 11-886 | 7.5 | 19-326 | 25 | 15-305 | 25 | | | | | | | | |
| jam_honey | 0.00 | 0.00 | 0.00 | 0.00 | 17-688 | 35 | 17-050 | 20 | 17-065 | 10 | 17-078 | 35 | | | | | | | | | | | | | | |
| mashed_butter_dunno | 4.22 | 2.25 | 1.29 | 0.33 | 13-490 | 89 | 12-313 | 6 | 17-655 | 2.5 | 17-685 | 1.25 | 17-661 | 1.25 | | | | | | | | | | | | |
| mashed_butter_fat | 4.30 | 2.70 | 1.08 | 0.15 | 13-490 | 89 | 12-313 | 6 | 17-685 | 2.5 | 17-661 | 2.5 | | | | | | | | | | | | | | |
| mashed_butter_lowfat | 3.20 | 1.38 | 1.17 | 0.40 | 13-490 | 89 | 12-313 | 6 | 17-656 | 5 | | | | | | | | | | | | | | | | |
| mashed_butter_spread_fat | 4.15 | 1.80 | 1.51 | 0.51 | 13-490 | 89 | 12-313 | 6 | 17-655 | 5 | | | | | | | | | | | | | | | | |
| mashed_butter_spread_lowfat | 3.20 | 1.38 | 1.17 | 0.40 | 13-490 | 89 | 12-313 | 6 | 17-656 | 5 | | | | | | | | | | | | | | | | |
| mashed_fat_dunno | 5.19 | 0.42 | 3.00 | 1.48 | 13-490 | 89 | 12-313 | 6 | 17-686 | 5 | | | | | | | | | | | | | | | | |
| mashed_fat_none | 0.21 | 0.10 | 0.04 | 0.01 | 13-490 | 93 | 12-313 | 7 | | | | | | | | | | | | | | | | | | |
| mashed_fat_other | 5.15 | 2.90 | 1.70 | 0.16 | 13-490 | 89 | 12-313 | 6 | 81204000 | 2.5 | 17-487 | 2.5 | | | | | | | | | | | | | | |
| mashed_lard | 5.14 | 2.11 | 2.20 | 0.51 | 13-490 | 89 | 12-313 | 6 | 17-010 | 5 | | | | | | | | | | | | | | | | |
| mashed_marg_hard | 4.01 | 1.41 | 1.73 | 0.62 | 13-490 | 89 | 12-313 | 6 | 12-500 | 5 | | | | | | | | | | | | | | | | |
| mashed_marg_poly_chol | 2.04 | 0.52 | 0.61 | 0.76 | 13-490 | 89 | 12-313 | 6 | 12-499 | 5 | | | | | | | | | | | | | | | | |
| mashed_marg_poly_dunno | 2.59 | 0.63 | 0.76 | 1.01 | 13-490 | 89 | 12-313 | 6 | 12-499 | 2.5 | 12-503 | 2.5 | | | | | | | | | | | | | | |
| mashed_marg_poly_fat | 3.15 | 0.75 | 0.92 | 1.27 | 13-490 | 89 | 12-313 | 6 | 12-503 | 5 | | | | | | | | | | | | | | | | |
| mashed_marg_poly_lowfat | 2.04 | 0.52 | 0.61 | 0.76 | 13-490 | 89 | 12-313 | 6 | 12-499 | 5 | | | | | | | | | | | | | | | | |
| mashed_marg_poly_vlowfat | 2.04 | 0.52 | 0.61 | 0.76 | 13-490 | 89 | 12-313 | 6 | 12-499 | 5 | | | | | | | | | | | | | | | | |
| mashed_marg_soya_chol | 2.04 | 0.52 | 0.61 | 0.76 | 13-490 | 89 | 12-313 | 6 | 12-499 | 5 | | | | | | | | | | | | | | | | |
| mashed_marg_soya_dunno | 2.59 | 0.63 | 0.76 | 1.01 | 13-490 | 89 | 12-313 | 6 | 12-499 | 2.5 | 12-503 | 2.5 | | | | | | | | | | | | | | |
| mashed_marg_soya_fat | 3.15 | 0.75 | 0.92 | 1.27 | 13-490 | 89 | 12-313 | 6 | 12-503 | 5 | | | | | | | | | | | | | | | | |
| mashed_marg_soya_lowfat | 2.04 | 0.52 | 0.61 | 0.76 | 13-490 | 89 | 12-313 | 6 | 12-499 | 5 | | | | | | | | | | | | | | | | |
| mashed_marg_soya_vlowfat | 2.04 | 0.52 | 0.61 | 0.76 | 13-490 | 89 | 12-313 | 6 | 12-499 | 5 | | | | | | | | | | | | | | | | |
| mashed_oil_olive | 5.19 | 0.81 | 3.68 | 0.42 | 13-490 | 89 | 12-313 | 6 | 17-038 | 5 | | | | | | | | | | | | | | | | |
| mashed_oil_other | 4.56 | 0.63 | 2.04 | 1.64 | 13-490 | 89 | 12-313 | 6 | 17-033 | 1.5 | 17-040 | 1 | 17-686 | 1.875 | 17-377 | 0.625 | | | | | | | | | | |
| mashed_oil_rapeseed | 5.19 | 0.42 | 3.00 | 1.48 | 13-490 | 89 | 12-313 | 6 | 17-041 | 5 | | | | | | | | | | | | | | | | |
| mashed_oil_sunflower | 5.19 | 0.69 | 1.06 | 3.18 | 13-490 | 89 | 12-313 | 6 | 17-045 | 5 | | | | | | | | | | | | | | | | |
| mashed_oil_veg | 5.19 | 0.42 | 3.00 | 1.48 | 13-490 | 89 | 12-313 | 6 | 17-686 | 5 | | | | | | | | | | | | | | | | |
| mashed_s_dairy_chol | 2.14 | 0.58 | 0.90 | 0.50 | 13-490 | 89 | 12-313 | 6 | 12-497 | 5 | | | | | | | | | | | | | | | | |
| mashed_s_dairy_dunno | 2.68 | 0.73 | 1.22 | 0.55 | 13-490 | 89 | 12-313 | 6 | 12-497 | 2.5 | 12-504 | 2.5 | | | | | | | | | | | | | | |
| mashed_s_dairy_fat | 3.22 | 0.87 | 1.53 | 0.61 | 13-490 | 89 | 12-313 | 6 | 12-504 | 5 | | | | | | | | | | | | | | | | |
| mashed_s_dairy_lowfat | 2.14 | 0.58 | 0.90 | 0.50 | 13-490 | 89 | 12-313 | 6 | 12-497 | 5 | | | | | | | | | | | | | | | | |
| mashed_s_dairy_vlowfat | 2.14 | 0.58 | 0.90 | 0.50 | 13-490 | 89 | 12-313 | 6 | 12-497 | 5 | | | | | | | | | | | | | | | | |
| mashed_s_dunno_chol | 2.09 | 0.55 | 0.76 | 0.63 | 13-490 | 89 | 12-313 | 6 | 12-499 | 2.5 | 12-497 | 2.5 | | | | | | | | | | | | | | |
| mashed_s_dunno_dunno | 2.64 | 0.68 | 0.99 | 0.78 | 13-490 | 89 | 12-313 | 6 | 12-499 | 1.25 | 12-503 | 1.25 | 12-497 | 1.25 | 12-504 | 1.25 | | | | | | | | | | |
| mashed_s_dunno_fat | 3.19 | 0.81 | 1.22 | 0.94 | 13-490 | 89 | 12-313 | 6 | 12-503 | 2.5 | 12-504 | 2.5 | | | | | | | | | | | | | | |
| mashed_s_dunno_lowfat | 2.09 | 0.55 | 0.76 | 0.63 | 13-490 | 89 | 12-313 | 6 | 12-497 | 2.5 | 12-499 | 2.5 | | | | | | | | | | | | | | |
| mashed_s_dunno_vlowfat | 2.09 | 0.55 | 0.76 | 0.63 | 13-490 | 89 | 12-313 | 6 | 12-497 | 2.5 | 12-499 | 2.5 | | | | | | | | | | | | | | |
| mashed_s_olive_chol | 2.14 | 0.54 | 1.12 | 0.33 | 13-490 | 89 | 12-313 | 6 | 12-498 | 5 | | | | | | | | | | | | | | | | |
| mashed_s_olive_dunno | 2.64 | 0.64 | 1.36 | 0.46 | 13-490 | 89 | 12-313 | 6 | 12-498 | 2.5 | 12-505 | 2.5 | | | | | | | | | | | | | | |
| mashed_s_olive_fat | 3.15 | 0.75 | 1.60 | 0.59 | 13-490 | 89 | 12-313 | 6 | 12-505 | 5 | | | | | | | | | | | | | | | | |

**Table S1.** Individual FA and major nutrient calculation in the individual FA dataset (MCW + USDA). USDA food codes are highlighted in red.

| Food item | Grams per 100g of food item[1] | | | | Codes for foods from MCW and USDA and the fraction (%) assigned to each code[2] | | | | | | | | | | | | | | | | | | | | | |
|---|---|---|---|---|---|---|---|---|---|---|---|---|---|---|---|---|---|---|---|---|---|---|---|---|---|---|
| | Total fat | Total SFAs | Total MUFAs | Total PUFAs | Code 1 | Fraction 1 | Code 2 | Fraction 2 | Code 3 | Fraction 3 | Code 4 | Fraction 4 | Code 5 | Fraction 5 | Code 6 | Fraction 6 | Code 7 | Fraction 7 | Code 8 | Fraction 8 | Code 9 | Fraction 9 | Code 10 | Fraction 10 | Code 11 | Fraction 11 |
| mashed_s_olive_lowfat | 2.14 | 0.54 | 1.12 | 0.33 | 13-490 | 89 | 12-313 | 6 | 12-498 | 5 | | | | | | | | | | | | | | | | |
| mashed_s_olive_vlowfat | 2.14 | 0.54 | 1.12 | 0.33 | 13-490 | 89 | 12-313 | 6 | 12-498 | 5 | | | | | | | | | | | | | | | | |
| mayo | 49.17 | 3.77 | 29.60 | 13.31 | 17-654 | 50 | 17-041 | 11 | 17-339 | 9 | 17-377 | 20 | 17-063 | 7.5 | 12-939 | 2.5 | | | | | | | | | | |
| mayo_lowfat | 16.39 | 2.24 | 5.47 | 7.94 | 83204000 | 50 | 17-041 | 4.5 | 17-339 | 11 | 17-377 | 24 | 17-063 | 8 | 12-939 | 2.5 | | | | | | | | | | |
| meat_bacon_nofat | 21.60 | 8.10 | 9.00 | 2.80 | 19-500 | 100 | | | | | | | | | | | | | | | | | | | | |
| meat_bacon_withfat | 23.86 | 8.77 | 10.17 | 3.17 | 19-500 | 33.4 | 19-503 | 33.3 | 19-018 | 33.3 | | | | | | | | | | | | | | | | |
| meat_beef_nofat | 8.65 | 3.68 | 3.70 | 0.50 | 18-022 | 25 | 18-088 | 25 | 18-080 | 25 | 18-042 | 25 | | | | | | | | | | | | | | |
| meat_beef_withfat | 13.45 | 5.73 | 5.80 | 0.62 | 18-009 | 25 | 18-089 | 25 | 18-470 | 25 | 18-042 | 25 | | | | | | | | | | | | | | |
| meat_ham_nofat | 3.30 | 1.10 | 1.39 | 0.52 | 19-496 | 100 | | | | | | | | | | | | | | | | | | | | |
| meat_ham_withfat | 14.99 | 5.16 | 6.59 | 2.15 | 19-021 | 90 | 19-517 | 10 | | | | | | | | | | | | | | | | | | |
| meat_lamb_nofat | 12.26 | 5.61 | 4.72 | 0.67 | 18-179 | 30 | 18-141 | 30 | 18-186 | 20 | 18-159 | 20 | | | | | | | | | | | | | | |
| meat_lamb_withfat | 22.14 | 10.59 | 8.53 | 1.10 | 18-180 | 30 | 18-169 | 30 | 18-187 | 20 | 18-159 | 20 | | | | | | | | | | | | | | |
| meat_liverpate | 22.81 | 6.62 | 7.93 | 2.67 | 18-413 | 19.5 | 17-033 | 1 | 18-417 | 18 | 11-886 | 1 | 17-685 | 0.5 | 19-317 | 60 | | | | | | | | | | |
| meat_other | 26.10 | 7.95 | 13.07 | 3.67 | 18-374 | 64 | 18-390 | 25 | 18-383 | 11 | | | | | | | | | | | | | | | | |
| meat_pork_nofat | 5.70 | 1.98 | 2.43 | 0.72 | 18-534 | 33.4 | 18-515 | 33.3 | 18-527 | 33.3 | | | | | | | | | | | | | | | | |
| meat_pork_withfat | 11.27 | 4.02 | 4.94 | 1.47 | 18-535 | 33.4 | 18-517 | 33.3 | 18-528 | 33.3 | | | | | | | | | | | | | | | | |
| meat_sausage | 23.18 | 8.42 | 9.81 | 3.50 | 19-657 | 25 | 19-512 | 75 | | | | | | | | | | | | | | | | | | |
| milk_chol_cereal | 0.30 | 0.13 | 0.06 | 0.01 | 12-307 | 100 | | | | | | | | | | | | | | | | | | | | |
| milk_chol_coffee | 0.30 | 0.13 | 0.06 | 0.01 | 12-307 | 100 | | | | | | | | | | | | | | | | | | | | |
| milk_chol_glass | 0.30 | 0.13 | 0.06 | 0.01 | 12-307 | 100 | | | | | | | | | | | | | | | | | | | | |
| milk_chol_tea | 0.30 | 0.13 | 0.06 | 0.01 | 12-307 | 100 | | | | | | | | | | | | | | | | | | | | |
| milk_dontknow_cereal | 1.80 | 1.13 | 0.44 | 0.04 | 12-313 | 60 | 12-307 | 20 | 12-596 | 20 | | | | | | | | | | | | | | | | |
| milk_dontknow_coffee | 1.80 | 1.13 | 0.44 | 0.04 | 12-313 | 60 | 12-307 | 20 | 12-596 | 20 | | | | | | | | | | | | | | | | |
| milk_dontknow_glass | 1.80 | 1.13 | 0.44 | 0.04 | 12-313 | 60 | 12-307 | 20 | 12-596 | 20 | | | | | | | | | | | | | | | | |
| milk_dontknow_tea | 1.80 | 1.13 | 0.44 | 0.04 | 12-313 | 60 | 12-307 | 20 | 12-596 | 20 | | | | | | | | | | | | | | | | |
| milk_goatsheep_cereal | 3.70 | 2.37 | 0.94 | 0.16 | 12-328 | 100 | | | | | | | | | | | | | | | | | | | | |
| milk_goatsheep_coffee | 4.23 | 2.67 | 1.07 | 0.21 | 12-328 | 75 | 12-329 | 25 | | | | | | | | | | | | | | | | | | |
| milk_goatsheep_glass | 4.23 | 2.67 | 1.07 | 0.21 | 12-328 | 75 | 12-329 | 25 | | | | | | | | | | | | | | | | | | |
| milk_goatsheep_tea | 4.23 | 2.67 | 1.07 | 0.21 | 12-328 | 75 | 12-329 | 25 | | | | | | | | | | | | | | | | | | |
| milk_other_cereal | 1.53 | 0.70 | 0.54 | 0.12 | 12-313 | 27 | 11350000 | 40 | 11114330 | 33 | | | | | | | | | | | | | | | | |
| milk_other_coffee | 1.53 | 0.70 | 0.54 | 0.12 | 12-313 | 27 | 11350000 | 40 | 11114330 | 33 | | | | | | | | | | | | | | | | |
| milk_other_glass | 1.53 | 0.70 | 0.54 | 0.12 | 12-313 | 27 | 11350000 | 40 | 11114330 | 33 | | | | | | | | | | | | | | | | |
| milk_other_tea | 1.53 | 0.70 | 0.54 | 0.12 | 12-313 | 27 | 11350000 | 40 | 11114330 | 33 | | | | | | | | | | | | | | | | |
| milk_powdered_cereal | 0.30 | 0.13 | 0.06 | 0.01 | 12-307 | 100 | | | | | | | | | | | | | | | | | | | | |
| milk_powdered_coffee | 18.10 | 16.50 | 0.58 | 0.02 | 11810000 | 50 | 12210400 | 50 | | | | | | | | | | | | | | | | | | |
| milk_powdered_glass | 0.30 | 0.13 | 0.06 | 0.01 | 12-307 | 100 | | | | | | | | | | | | | | | | | | | | |
| milk_powdered_tea | 0.72 | 0.47 | 0.19 | 0.03 | 11810000 | 100 | | | | | | | | | | | | | | | | | | | | |
| milk_riceoatveg_cereal | 1.32 | 0.12 | 0.48 | 0.65 | 12-524 | 50 | 11350000 | 50 | | | | | | | | | | | | | | | | | | |
| milk_riceoatveg_coffee | 1.32 | 0.12 | 0.48 | 0.65 | 12-524 | 50 | 11350000 | 50 | | | | | | | | | | | | | | | | | | |
| milk_riceoatveg_glass | 1.32 | 0.12 | 0.48 | 0.65 | 12-524 | 50 | 11350000 | 50 | | | | | | | | | | | | | | | | | | |
| milk_riceoatveg_tea | 1.32 | 0.12 | 0.48 | 0.65 | 12-524 | 50 | 11350000 | 50 | | | | | | | | | | | | | | | | | | |
| milk_semi_cereal | 1.70 | 1.07 | 0.40 | 0.04 | 12-313 | 100 | | | | | | | | | | | | | | | | | | | | |
| milk_semi_coffee | 1.70 | 1.07 | 0.40 | 0.04 | 12-313 | 100 | | | | | | | | | | | | | | | | | | | | |
| milk_semi_glass | 1.70 | 1.07 | 0.40 | 0.04 | 12-313 | 100 | | | | | | | | | | | | | | | | | | | | |
| milk_semi_tea | 1.70 | 1.07 | 0.40 | 0.04 | 12-313 | 100 | | | | | | | | | | | | | | | | | | | | |
| milk_skimmed_cereal | 0.30 | 0.13 | 0.06 | 0.01 | 12-307 | 100 | | | | | | | | | | | | | | | | | | | | |
| milk_skimmed_coffee | 0.30 | 0.13 | 0.06 | 0.01 | 12-307 | 100 | | | | | | | | | | | | | | | | | | | | |
| milk_skimmed_glass | 0.30 | 0.13 | 0.06 | 0.01 | 12-307 | 100 | | | | | | | | | | | | | | | | | | | | |
| milk_skimmed_tea | 0.30 | 0.13 | 0.06 | 0.01 | 12-307 | 100 | | | | | | | | | | | | | | | | | | | | |
| milk_soya_ca_cereal | 1.60 | 0.24 | 0.33 | 1.10 | 12-524 | 100 | | | | | | | | | | | | | | | | | | | | |
| milk_soya_ca_coffee | 1.60 | 0.24 | 0.33 | 1.10 | 12-524 | 100 | | | | | | | | | | | | | | | | | | | | |
| milk_soya_ca_glass | 1.60 | 0.24 | 0.33 | 1.10 | 12-524 | 100 | | | | | | | | | | | | | | | | | | | | |
| milk_soya_ca_tea | 1.60 | 0.24 | 0.33 | 1.10 | 12-524 | 100 | | | | | | | | | | | | | | | | | | | | |
| milk_soya_noca_cereal | 1.60 | 0.24 | 0.33 | 1.10 | 12-524 | 100 | | | | | | | | | | | | | | | | | | | | |
| milk_soya_noca_coffee | 1.60 | 0.24 | 0.33 | 1.10 | 12-524 | 100 | | | | | | | | | | | | | | | | | | | | |
| milk_soya_noca_glass | 1.60 | 0.24 | 0.33 | 1.10 | 12-524 | 100 | | | | | | | | | | | | | | | | | | | | |
| milk_soya_noca_tea | 1.60 | 0.24 | 0.33 | 1.10 | 12-524 | 100 | | | | | | | | | | | | | | | | | | | | |
| milk_whole_cereal | 3.60 | 2.29 | 0.96 | 0.09 | 12-596 | 100 | | | | | | | | | | | | | | | | | | | | |
| milk_whole_coffee | 3.60 | 2.29 | 0.96 | 0.09 | 12-596 | 100 | | | | | | | | | | | | | | | | | | | | |
| milk_whole_glass | 3.60 | 2.29 | 0.96 | 0.09 | 12-596 | 100 | | | | | | | | | | | | | | | | | | | | |
| milk_whole_tea | 3.60 | 2.29 | 0.96 | 0.09 | 12-596 | 100 | | | | | | | | | | | | | | | | | | | | |
| oatcak_s_butter_dunno_med | 80.65 | 43.12 | 25.23 | 6.42 | 17-655 | 50 | 17-685 | 25 | 17-661 | 25 | | | | | | | | | | | | | | | | |
| oatcak_s_butter_dunno_thick | 80.65 | 43.12 | 25.23 | 6.42 | 17-655 | 50 | 17-685 | 25 | 17-661 | 25 | | | | | | | | | | | | | | | | |
| oatcak_s_butter_dunno_thin | 80.65 | 43.12 | 25.23 | 6.42 | 17-655 | 50 | 17-685 | 25 | 17-661 | 25 | | | | | | | | | | | | | | | | |
| oatcak_s_butter_fat_med | 82.20 | 52.09 | 20.91 | 2.83 | 17-685 | 50 | 17-661 | 50 | | | | | | | | | | | | | | | | | | |
| oatcak_s_butter_fat_thick | 82.20 | 52.09 | 20.91 | 2.83 | 17-685 | 50 | 17-661 | 50 | | | | | | | | | | | | | | | | | | |
| oatcak_s_butter_fat_thin | 82.20 | 52.09 | 20.91 | 2.83 | 17-685 | 50 | 17-661 | 50 | | | | | | | | | | | | | | | | | | |
| oatcak_s_butter_lowfat_med | 60.20 | 25.70 | 22.66 | 7.75 | 17-656 | 100 | | | | | | | | | | | | | | | | | | | | |
| oatcak_s_butter_lowfat_thick | 60.20 | 25.70 | 22.66 | 7.75 | 17-656 | 100 | | | | | | | | | | | | | | | | | | | | |
| oatcak_s_butter_lowfat_thin | 60.20 | 25.70 | 22.66 | 7.75 | 17-656 | 100 | | | | | | | | | | | | | | | | | | | | |
| oatcak_s_buttspr_fat_med | 79.10 | 34.15 | 29.54 | 10.00 | 17-655 | 100 | | | | | | | | | | | | | | | | | | | | |
| oatcak_s_buttspr_fat_thick | 79.10 | 34.15 | 29.54 | 10.00 | 17-655 | 100 | | | | | | | | | | | | | | | | | | | | |
| oatcak_s_buttspr_fat_thin | 79.10 | 34.15 | 29.54 | 10.00 | 17-655 | 100 | | | | | | | | | | | | | | | | | | | | |
| oatcak_s_buttspr_lowfat_med | 60.20 | 25.70 | 22.66 | 7.75 | 17-656 | 100 | | | | | | | | | | | | | | | | | | | | |
| oatcak_s_buttspr_lowfat_thick | 60.20 | 25.70 | 22.66 | 7.75 | 17-656 | 100 | | | | | | | | | | | | | | | | | | | | |

**Table S1.** Individual FA and major nutrient calculation in the individual FA dataset (MCW + USDA). USDA food codes are highlighted in red.

| Food item | Grams per 100g of food item[1] | | | | Codes for foods from MCW and USDA and the fraction (%) assigned to each code[2] | | | | | | | | | | | | | | | | | | | | | |
|---|---|---|---|---|---|---|---|---|---|---|---|---|---|---|---|---|---|---|---|---|---|---|---|---|---|---|
| | Total fat | Total SFAs | Total MUFAs | Total PUFAs | Code 1 | Fraction 1 | Code 2 | Fraction 2 | Code 3 | Fraction 3 | Code 4 | Fraction 4 | Code 5 | Fraction 5 | Code 6 | Fraction 6 | Code 7 | Fraction 7 | Code 8 | Fraction 8 | Code 9 | Fraction 9 | Code 10 | Fraction 10 | Code 11 | Fraction 11 |
| oatcak_s_buttspr_lowfat_thin | 60.20 | 25.70 | 22.66 | 7.75 | 17-656 | 100 | | | | | | | | | | | | | | | | | | | | |
| oatcak_s_dairy_chol_med | 39.00 | 9.76 | 17.39 | 9.82 | 12-497 | 100 | | | | | | | | | | | | | | | | | | | | |
| oatcak_s_dairy_chol_thick | 39.00 | 9.76 | 17.39 | 9.82 | 12-497 | 100 | | | | | | | | | | | | | | | | | | | | |
| oatcak_s_dairy_chol_thin | 39.00 | 9.76 | 17.39 | 9.82 | 12-497 | 100 | | | | | | | | | | | | | | | | | | | | |
| oatcak_s_dairy_dunno_med | 49.80 | 12.69 | 23.67 | 10.86 | 12-497 | 50 | 12-504 | 50 | | | | | | | | | | | | | | | | | | |
| oatcak_s_dairy_dunno_thick | 49.80 | 12.69 | 23.67 | 10.86 | 12-497 | 50 | 12-504 | 50 | | | | | | | | | | | | | | | | | | |
| oatcak_s_dairy_dunno_thin | 49.80 | 12.69 | 23.67 | 10.86 | 12-497 | 50 | 12-504 | 50 | | | | | | | | | | | | | | | | | | |
| oatcak_s_dairy_fat_med | 60.60 | 15.61 | 29.95 | 11.90 | 12-504 | 100 | | | | | | | | | | | | | | | | | | | | |
| oatcak_s_dairy_fat_thick | 60.60 | 15.61 | 29.95 | 11.90 | 12-504 | 100 | | | | | | | | | | | | | | | | | | | | |
| oatcak_s_dairy_fat_thin | 60.60 | 15.61 | 29.95 | 11.90 | 12-504 | 100 | | | | | | | | | | | | | | | | | | | | |
| oatcak_s_dairy_lowfat_med | 39.00 | 9.76 | 17.39 | 9.82 | 12-497 | 100 | | | | | | | | | | | | | | | | | | | | |
| oatcak_s_dairy_lowfat_thick | 39.00 | 9.76 | 17.39 | 9.82 | 12-497 | 100 | | | | | | | | | | | | | | | | | | | | |
| oatcak_s_dairy_lowfat_thin | 39.00 | 9.76 | 17.39 | 9.82 | 12-497 | 100 | | | | | | | | | | | | | | | | | | | | |
| oatcak_s_dairy_vlowfat_med | 39.00 | 9.76 | 17.39 | 9.82 | 12-497 | 100 | | | | | | | | | | | | | | | | | | | | |
| oatcak_s_dairy_vlowfat_thick | 39.00 | 9.76 | 17.39 | 9.82 | 12-497 | 100 | | | | | | | | | | | | | | | | | | | | |
| oatcak_s_dairy_vlowfat_thin | 39.00 | 9.76 | 17.39 | 9.82 | 12-497 | 100 | | | | | | | | | | | | | | | | | | | | |
| oatcak_s_dunno_chol_med | 37.95 | 9.16 | 14.45 | 12.39 | 12-497 | 50 | 12-499 | 50 | | | | | | | | | | | | | | | | | | |
| oatcak_s_dunno_chol_thick | 37.95 | 9.16 | 14.45 | 12.39 | 12-497 | 50 | 12-499 | 50 | | | | | | | | | | | | | | | | | | |
| oatcak_s_dunno_chol_thin | 37.95 | 9.16 | 14.45 | 12.39 | 12-497 | 50 | 12-499 | 50 | | | | | | | | | | | | | | | | | | |
| oatcak_s_dunno_dunno_med | 48.93 | 11.78 | 19.14 | 15.46 | 12-497 | 25 | 12-499 | 25 | 12-503 | 25 | 12-504 | 25 | | | | | | | | | | | | | | |
| oatcak_s_dunno_dunno_thick | 48.93 | 11.78 | 19.14 | 15.46 | 12-497 | 25 | 12-499 | 25 | 12-503 | 25 | 12-504 | 25 | | | | | | | | | | | | | | |
| oatcak_s_dunno_dunno_thin | 48.93 | 11.78 | 19.14 | 15.46 | 12-497 | 25 | 12-499 | 25 | 12-503 | 25 | 12-504 | 25 | | | | | | | | | | | | | | |
| oatcak_s_dunno_fat_med | 59.90 | 14.41 | 23.83 | 18.54 | 12-503 | 50 | 12-504 | 50 | | | | | | | | | | | | | | | | | | |
| oatcak_s_dunno_fat_thick | 59.90 | 14.41 | 23.83 | 18.54 | 12-503 | 50 | 12-504 | 50 | | | | | | | | | | | | | | | | | | |
| oatcak_s_dunno_fat_thin | 59.90 | 14.41 | 23.83 | 18.54 | 12-503 | 50 | 12-504 | 50 | | | | | | | | | | | | | | | | | | |
| oatcak_s_dunno_lowfat_med | 37.95 | 9.16 | 14.45 | 12.39 | 12-497 | 50 | 12-499 | 50 | | | | | | | | | | | | | | | | | | |
| oatcak_s_dunno_lowfat_thick | 37.95 | 9.16 | 14.45 | 12.39 | 12-497 | 50 | 12-499 | 50 | | | | | | | | | | | | | | | | | | |
| oatcak_s_dunno_lowfat_thin | 37.95 | 9.16 | 14.45 | 12.39 | 12-497 | 50 | 12-499 | 50 | | | | | | | | | | | | | | | | | | |
| oatcak_s_dunno_vlowfat_med | 37.95 | 9.16 | 14.45 | 12.39 | 12-497 | 50 | 12-499 | 50 | | | | | | | | | | | | | | | | | | |
| oatcak_s_dunno_vlowfat_thick | 37.95 | 9.16 | 14.45 | 12.39 | 12-497 | 50 | 12-499 | 50 | | | | | | | | | | | | | | | | | | |
| oatcak_s_dunno_vlowfat_thin | 37.95 | 9.16 | 14.45 | 12.39 | 12-497 | 50 | 12-499 | 50 | | | | | | | | | | | | | | | | | | |
| oatcak_s_hardmarg_med | 76.40 | 26.41 | 34.00 | 12.21 | 12-500 | 100 | | | | | | | | | | | | | | | | | | | | |
| oatcak_s_hardmarg_thick | 76.40 | 26.41 | 34.00 | 12.21 | 12-500 | 100 | | | | | | | | | | | | | | | | | | | | |
| oatcak_s_hardmarg_thin | 76.40 | 26.41 | 34.00 | 12.21 | 12-500 | 100 | | | | | | | | | | | | | | | | | | | | |
| oatcak_s_olive_chol_med | 38.90 | 8.90 | 21.68 | 6.36 | 12-498 | 100 | | | | | | | | | | | | | | | | | | | | |
| oatcak_s_olive_chol_thick | 38.90 | 8.90 | 21.68 | 6.36 | 12-498 | 100 | | | | | | | | | | | | | | | | | | | | |
| oatcak_s_olive_chol_thin | 38.90 | 8.90 | 21.68 | 6.36 | 12-498 | 100 | | | | | | | | | | | | | | | | | | | | |
| oatcak_s_olive_dunno_med | 49.00 | 11.06 | 26.53 | 8.95 | 12-498 | 50 | 12-505 | 50 | | | | | | | | | | | | | | | | | | |
| oatcak_s_olive_dunno_thick | 49.00 | 11.06 | 26.53 | 8.95 | 12-498 | 50 | 12-505 | 50 | | | | | | | | | | | | | | | | | | |
| oatcak_s_olive_dunno_thin | 49.00 | 11.06 | 26.53 | 8.95 | 12-498 | 50 | 12-505 | 50 | | | | | | | | | | | | | | | | | | |
| oatcak_s_olive_fat_med | 59.10 | 13.22 | 31.37 | 11.53 | 12-505 | 100 | | | | | | | | | | | | | | | | | | | | |
| oatcak_s_olive_fat_thick | 59.10 | 13.22 | 31.37 | 11.53 | 12-505 | 100 | | | | | | | | | | | | | | | | | | | | |
| oatcak_s_olive_fat_thin | 59.10 | 13.22 | 31.37 | 11.53 | 12-505 | 100 | | | | | | | | | | | | | | | | | | | | |
| oatcak_s_olive_lowfat_med | 38.90 | 8.90 | 21.68 | 6.36 | 12-498 | 100 | | | | | | | | | | | | | | | | | | | | |
| oatcak_s_olive_lowfat_thick | 38.90 | 8.90 | 21.68 | 6.36 | 12-498 | 100 | | | | | | | | | | | | | | | | | | | | |
| oatcak_s_olive_lowfat_thin | 38.90 | 8.90 | 21.68 | 6.36 | 12-498 | 100 | | | | | | | | | | | | | | | | | | | | |
| oatcak_s_olive_vlowfat_med | 38.90 | 8.90 | 21.68 | 6.36 | 12-498 | 100 | | | | | | | | | | | | | | | | | | | | |
| oatcak_s_olive_vlowfat_thick | 38.90 | 8.90 | 21.68 | 6.36 | 12-498 | 100 | | | | | | | | | | | | | | | | | | | | |
| oatcak_s_olive_vlowfat_thin | 38.90 | 8.90 | 21.68 | 6.36 | 12-498 | 100 | | | | | | | | | | | | | | | | | | | | |
| oatcak_s_other_med | 68.67 | 33.71 | 21.43 | 9.30 | 81204000 | 18 | 17-685 | 16 | 17-661 | 16 | 12-499 | 12.5 | 12-503 | 12.5 | 12-497 | 12.5 | 12-504 | 12.5 | | | | | | | | |
| oatcak_s_other_thick | 68.67 | 33.71 | 21.43 | 9.30 | 81204000 | 18 | 17-685 | 16 | 17-661 | 16 | 12-499 | 12.5 | 12-503 | 12.5 | 12-497 | 12.5 | 12-504 | 12.5 | | | | | | | | |
| oatcak_s_other_thin | 68.67 | 33.71 | 21.43 | 9.30 | 81204000 | 18 | 17-685 | 16 | 17-661 | 16 | 12-499 | 12.5 | 12-503 | 12.5 | 12-497 | 12.5 | 12-504 | 12.5 | | | | | | | | |
| oatcak_s_poly_chol_med | 36.90 | 8.55 | 11.50 | 14.96 | 12-499 | 100 | | | | | | | | | | | | | | | | | | | | |
| oatcak_s_poly_chol_thick | 36.90 | 8.55 | 11.50 | 14.96 | 12-499 | 100 | | | | | | | | | | | | | | | | | | | | |
| oatcak_s_poly_chol_thin | 36.90 | 8.55 | 11.50 | 14.96 | 12-499 | 100 | | | | | | | | | | | | | | | | | | | | |
| oatcak_s_poly_dunno_med | 48.05 | 10.88 | 14.60 | 20.07 | 12-499 | 50 | 12-503 | 50 | | | | | | | | | | | | | | | | | | |
| oatcak_s_poly_dunno_thick | 48.05 | 10.88 | 14.60 | 20.07 | 12-499 | 50 | 12-503 | 50 | | | | | | | | | | | | | | | | | | |
| oatcak_s_poly_dunno_thin | 48.05 | 10.88 | 14.60 | 20.07 | 12-499 | 50 | 12-503 | 50 | | | | | | | | | | | | | | | | | | |
| oatcak_s_poly_fat_med | 59.20 | 13.21 | 17.70 | 25.17 | 12-503 | 100 | | | | | | | | | | | | | | | | | | | | |
| oatcak_s_poly_fat_thick | 59.20 | 13.21 | 17.70 | 25.17 | 12-503 | 100 | | | | | | | | | | | | | | | | | | | | |
| oatcak_s_poly_fat_thin | 59.20 | 13.21 | 17.70 | 25.17 | 12-503 | 100 | | | | | | | | | | | | | | | | | | | | |
| oatcak_s_poly_lowfat_med | 36.90 | 8.55 | 11.50 | 14.96 | 12-499 | 100 | | | | | | | | | | | | | | | | | | | | |
| oatcak_s_poly_lowfat_thick | 36.90 | 8.55 | 11.50 | 14.96 | 12-499 | 100 | | | | | | | | | | | | | | | | | | | | |
| oatcak_s_poly_lowfat_thin | 36.90 | 8.55 | 11.50 | 14.96 | 12-499 | 100 | | | | | | | | | | | | | | | | | | | | |
| oatcak_s_poly_vlowfat_med | 36.90 | 8.55 | 11.50 | 14.96 | 12-499 | 100 | | | | | | | | | | | | | | | | | | | | |
| oatcak_s_poly_vlowfat_thick | 36.90 | 8.55 | 11.50 | 14.96 | 12-499 | 100 | | | | | | | | | | | | | | | | | | | | |
| oatcak_s_poly_vlowfat_thin | 36.90 | 8.55 | 11.50 | 14.96 | 12-499 | 100 | | | | | | | | | | | | | | | | | | | | |
| oatcak_s_soya_chol_med | 36.90 | 8.55 | 11.50 | 14.96 | 12-499 | 100 | | | | | | | | | | | | | | | | | | | | |
| oatcak_s_soya_chol_thick | 36.90 | 8.55 | 11.50 | 14.96 | 12-499 | 100 | | | | | | | | | | | | | | | | | | | | |
| oatcak_s_soya_chol_thin | 36.90 | 8.55 | 11.50 | 14.96 | 12-499 | 100 | | | | | | | | | | | | | | | | | | | | |
| oatcak_s_soya_dunno_med | 48.05 | 10.88 | 14.60 | 20.07 | 12-499 | 50 | 12-503 | 50 | | | | | | | | | | | | | | | | | | |
| oatcak_s_soya_dunno_thick | 48.05 | 10.88 | 14.60 | 20.07 | 12-499 | 50 | 12-503 | 50 | | | | | | | | | | | | | | | | | | |
| oatcak_s_soya_dunno_thin | 48.05 | 10.88 | 14.60 | 20.07 | 12-499 | 50 | 12-503 | 50 | | | | | | | | | | | | | | | | | | |
| oatcak_s_soya_fat_med | 59.20 | 13.21 | 17.70 | 25.17 | 12-503 | 100 | | | | | | | | | | | | | | | | | | | | |
| oatcak_s_soya_fat_thick | 59.20 | 13.21 | 17.70 | 25.17 | 12-503 | 100 | | | | | | | | | | | | | | | | | | | | |

**Table S1.** Individual FA and major nutrient calculation in the individual FA dataset (MCW + USDA). USDA food codes are highlighted in red.

| Food item | Grams per 100g of food item[1] | | | | Codes for foods from MCW and USDA and the fraction (%) assigned to each code[2] | | | | | | | | | | | | | | | | | | | | | |
|---|---|---|---|---|---|---|---|---|---|---|---|---|---|---|---|---|---|---|---|---|---|---|---|---|---|---|
| | Total fat | Total SFAs | Total MUFAs | Total PUFAs | Code 1 | Fraction 1 | Code 2 | Fraction 2 | Code 3 | Fraction 3 | Code 4 | Fraction 4 | Code 5 | Fraction 5 | Code 6 | Fraction 6 | Code 7 | Fraction 7 | Code 8 | Fraction 8 | Code 9 | Fraction 9 | Code 10 | Fraction 10 | Code 11 | Fraction 11 |
| oatcak_s_soya_fat_thin | 59.20 | 13.21 | 17.70 | 25.17 | 12-503 | 100 | | | | | | | | | | | | | | | | | | | | |
| oatcak_s_soya_lowfat_med | 36.90 | 8.55 | 11.50 | 14.96 | 12-499 | 100 | | | | | | | | | | | | | | | | | | | | |
| oatcak_s_soya_lowfat_thick | 36.90 | 8.55 | 11.50 | 14.96 | 12-499 | 100 | | | | | | | | | | | | | | | | | | | | |
| oatcak_s_soya_lowfat_thin | 36.90 | 8.55 | 11.50 | 14.96 | 12-499 | 100 | | | | | | | | | | | | | | | | | | | | |
| oatcak_s_soya_vlowfat_med | 36.90 | 8.55 | 11.50 | 14.96 | 12-499 | 100 | | | | | | | | | | | | | | | | | | | | |
| oatcak_s_soya_vlowfat_thick | 36.90 | 8.55 | 11.50 | 14.96 | 12-499 | 100 | | | | | | | | | | | | | | | | | | | | |
| oatcak_s_soya_vlowfat_thin | 36.90 | 8.55 | 11.50 | 14.96 | 12-499 | 100 | | | | | | | | | | | | | | | | | | | | |
| oatcakes | 20.00 | 5.67 | 9.32 | 3.87 | 11-823 | 100 | | | | | | | | | | | | | | | | | | | | |
| pancake_blini_chol | 7.68 | 0.91 | 4.09 | 2.01 | 11-886 | 26 | 12-940 | 12 | 17-686 | 6 | 12-307 | 56 | | | | | | | | | | | | | | |
| pancake_blini_dontknow | 8.52 | 1.46 | 4.31 | 2.03 | 11-886 | 26 | 12-940 | 12 | 17-686 | 6 | 12-313 | 33.6 | 12-307 | 11.2 | 12-596 | 11.2 | | | | | | | | | | |
| pancake_blini_goatsheep | 10.17 | 2.50 | 4.74 | 2.14 | 11-886 | 26 | 12-940 | 12 | 17-686 | 6 | 12-328 | 28 | 12-329 | 28 | | | | | | | | | | | | |
| pancake_blini_other | 8.37 | 1.23 | 4.36 | 2.07 | 11-886 | 26 | 12-940 | 12 | 17-686 | 6 | 12-313 | 15.12 | 11350000 | 22.4 | 11114330 | 18.48 | | | | | | | | | | |
| pancake_blini_powdered | 7.68 | 0.91 | 4.09 | 2.01 | 11-886 | 26 | 12-940 | 12 | 17-686 | 6 | 12-307 | 56 | | | | | | | | | | | | | | |
| pancake_blini_riceoatveg | 8.25 | 0.90 | 4.33 | 2.37 | 11-886 | 26 | 12-940 | 12 | 17-686 | 6 | 12-524 | 28 | 11350000 | 28 | | | | | | | | | | | | |
| pancake_blini_semi | 8.46 | 1.43 | 4.28 | 2.02 | 11-886 | 26 | 12-940 | 12 | 17-686 | 6 | 12-313 | 56 | | | | | | | | | | | | | | |
| pancake_blini_skimmed | 7.68 | 0.91 | 4.09 | 2.01 | 11-886 | 26 | 12-940 | 12 | 17-686 | 6 | 12-307 | 56 | | | | | | | | | | | | | | |
| pancake_blini_soya_ca | 8.41 | 0.97 | 4.25 | 2.62 | 11-886 | 26 | 12-940 | 12 | 17-686 | 6 | 12-524 | 56 | | | | | | | | | | | | | | |
| pancake_blini_soya_noca | 8.41 | 0.97 | 4.25 | 2.62 | 11-886 | 26 | 12-940 | 12 | 17-686 | 6 | 12-524 | 56 | | | | | | | | | | | | | | |
| pancake_blini_whole | 9.53 | 2.12 | 4.60 | 2.05 | 11-886 | 26 | 12-940 | 12 | 17-686 | 6 | 12-596 | 56 | | | | | | | | | | | | | | |
| pancake_crepe_chol | 7.68 | 0.91 | 4.09 | 2.01 | 11-886 | 26 | 12-940 | 12 | 17-686 | 6 | 12-307 | 56 | | | | | | | | | | | | | | |
| pancake_crepe_dontknow | 8.52 | 1.46 | 4.31 | 2.03 | 11-886 | 26 | 12-940 | 12 | 17-686 | 6 | 12-313 | 33.6 | 12-307 | 11.2 | 12-596 | 11.2 | | | | | | | | | | |
| pancake_crepe_goatsheep | 10.17 | 2.50 | 4.74 | 2.14 | 11-886 | 26 | 12-940 | 12 | 17-686 | 6 | 12-328 | 28 | 12-329 | 28 | | | | | | | | | | | | |
| pancake_crepe_other | 8.37 | 1.23 | 4.36 | 2.07 | 11-886 | 26 | 12-940 | 12 | 17-686 | 6 | 12-313 | 15.12 | 11350000 | 22.4 | 11114330 | 18.48 | | | | | | | | | | |
| pancake_crepe_powdered | 7.68 | 0.91 | 4.09 | 2.01 | 11-886 | 26 | 12-940 | 12 | 17-686 | 6 | 12-307 | 56 | | | | | | | | | | | | | | |
| pancake_crepe_riceoatveg | 8.25 | 0.90 | 4.33 | 2.37 | 11-886 | 26 | 12-940 | 12 | 17-686 | 6 | 12-524 | 28 | 11350000 | 28 | | | | | | | | | | | | |
| pancake_crepe_semi | 8.46 | 1.43 | 4.28 | 2.02 | 11-886 | 26 | 12-940 | 12 | 17-686 | 6 | 12-313 | 56 | | | | | | | | | | | | | | |
| pancake_crepe_skimmed | 7.68 | 0.91 | 4.09 | 2.01 | 11-886 | 26 | 12-940 | 12 | 17-686 | 6 | 12-307 | 56 | | | | | | | | | | | | | | |
| pancake_crepe_soya_ca | 8.41 | 0.97 | 4.25 | 2.62 | 11-886 | 26 | 12-940 | 12 | 17-686 | 6 | 12-524 | 56 | | | | | | | | | | | | | | |
| pancake_crepe_soya_noca | 8.41 | 0.97 | 4.25 | 2.62 | 11-886 | 26 | 12-940 | 12 | 17-686 | 6 | 12-524 | 56 | | | | | | | | | | | | | | |
| pancake_crepe_whole | 9.53 | 2.12 | 4.60 | 2.05 | 11-886 | 26 | 12-940 | 12 | 17-686 | 6 | 12-596 | 56 | | | | | | | | | | | | | | |
| pasta_brown | 0.40 | 0.06 | 0.05 | 0.15 | 11-720 | 100 | | | | | | | | | | | | | | | | | | | | |
| pasta_gf | 0.93 | 0.15 | 0.15 | 0.30 | 11-450 | 25 | 11-722 | 50 | 11-724 | 25 | | | | | | | | | | | | | | | | |
| pasta_white | 0.93 | 0.15 | 0.15 | 0.30 | 11-450 | 25 | 11-722 | 50 | 11-724 | 25 | | | | | | | | | | | | | | | | |
| pesto | 42.50 | 6.50 | 16.55 | 17.55 | 17-622 | 100 | | | | | | | | | | | | | | | | | | | | |
| pizza | 9.80 | 4.12 | 3.67 | 1.39 | 11-936 | 100 | | | | | | | | | | | | | | | | | | | | |
| pizza_gf | 9.80 | 4.12 | 3.67 | 1.39 | 11-936 | 100 | | | | | | | | | | | | | | | | | | | | |
| pnutbutter_nutella | 46.16 | 11.75 | 18.98 | 13.41 | 14-892 | 70 | 17-687 | 30 | | | | | | | | | | | | | | | | | | |
| por_milk_chol | 1.28 | 0.28 | 0.46 | 0.40 | 11-792 | 14 | 12-307 | 86 | | | | | | | | | | | | | | | | | | |
| por_milk_chol_driedfruit | 1.08 | 0.23 | 0.36 | 0.32 | 11-792 | 11 | 14-087 | 13 | 12-307 | 76 | | | | | | | | | | | | | | | | |
| por_milk_dontknow | 2.57 | 1.14 | 0.79 | 0.43 | 11-792 | 14 | 12-313 | 51.6 | 12-307 | 17.2 | 12-596 | 17.2 | | | | | | | | | | | | | | |
| por_milk_dontknow_driedfruit | 2.22 | 0.99 | 0.66 | 0.34 | 11-792 | 11 | 14-087 | 13 | 12-313 | 45.6 | 12-307 | 15.2 | 12-596 | 15.2 | | | | | | | | | | | | |
| por_milk_goatsheep | 5.11 | 2.73 | 1.44 | 0.61 | 11-792 | 14 | 12-328 | 43 | 12-329 | 43 | | | | | | | | | | | | | | | | |
| por_milk_goatsheep_driedfruit | 4.47 | 2.39 | 1.23 | 0.50 | 11-792 | 11 | 14-087 | 13 | 12-328 | 38 | 12-329 | 38 | | | | | | | | | | | | | | |
| por_milk_other | 2.34 | 0.77 | 0.87 | 0.49 | 11-792 | 14 | 12-313 | 23.22 | 11350000 | 34.4 | 11114330 | 28.38 | | | | | | | | | | | | | | |
| por_milk_other_driedfruit | 2.02 | 0.67 | 0.73 | 0.40 | 11-792 | 11 | 14-087 | 13 | 12-313 | 20.52 | 11350000 | 30.4 | 11114330 | 25.08 | | | | | | | | | | | | |
| por_milk_powdered | 1.28 | 0.28 | 0.46 | 0.40 | 11-792 | 14 | 12-307 | 86 | | | | | | | | | | | | | | | | | | |
| por_milk_powdered_driedfruit | 1.08 | 0.23 | 0.36 | 0.32 | 11-792 | 11 | 14-087 | 13 | 12-307 | 76 | | | | | | | | | | | | | | | | |
| por_milk_riceoatveg | 2.16 | 0.27 | 0.82 | 0.95 | 11-792 | 14 | 12-524 | 43 | 11350000 | 43 | | | | | | | | | | | | | | | | |
| por_milk_riceoatveg_driedfruit | 1.86 | 0.22 | 0.68 | 0.81 | 11-792 | 11 | 14-087 | 13 | 12-524 | 38 | 11350000 | 38 | | | | | | | | | | | | | | |
| por_milk_semi | 2.48 | 1.09 | 0.75 | 0.43 | 11-792 | 14 | 12-313 | 86 | | | | | | | | | | | | | | | | | | |
| por_milk_semi_driedfruit | 2.15 | 0.95 | 0.62 | 0.34 | 11-792 | 11 | 14-087 | 13 | 12-313 | 76 | | | | | | | | | | | | | | | | |
| por_milk_skimmed | 1.28 | 0.28 | 0.46 | 0.40 | 11-792 | 14 | 12-307 | 86 | | | | | | | | | | | | | | | | | | |
| por_milk_skimmed_driedfruit | 1.08 | 0.23 | 0.36 | 0.32 | 11-792 | 11 | 14-087 | 13 | 12-307 | 76 | | | | | | | | | | | | | | | | |
| por_milk_soya_ca | 2.40 | 0.37 | 0.69 | 1.34 | 11-792 | 14 | 12-524 | 86 | | | | | | | | | | | | | | | | | | |
| por_milk_soya_ca_driedfruit | 2.07 | 0.31 | 0.57 | 1.14 | 11-792 | 11 | 14-087 | 13 | 12-524 | 76 | | | | | | | | | | | | | | | | |
| por_milk_soya_noca | 2.40 | 0.37 | 0.69 | 1.34 | 11-792 | 14 | 12-524 | 86 | | | | | | | | | | | | | | | | | | |
| por_milk_soya_noca_driedfruit | 2.07 | 0.31 | 0.57 | 1.14 | 11-792 | 11 | 14-087 | 13 | 12-524 | 76 | | | | | | | | | | | | | | | | |
| por_milk_whole | 4.12 | 2.14 | 1.23 | 0.47 | 11-792 | 14 | 12-596 | 86 | | | | | | | | | | | | | | | | | | |
| por_milk_whole_driedfruit | 3.59 | 1.87 | 1.05 | 0.38 | 11-792 | 11 | 14-087 | 13 | 12-596 | 76 | | | | | | | | | | | | | | | | |
| por_water | 1.02 | 0.17 | 0.41 | 0.39 | 11-792 | 14 | 17-377 | 86 | | | | | | | | | | | | | | | | | | |
| por_water_driedfruit | 0.86 | 0.13 | 0.32 | 0.31 | 11-792 | 11 | 14-087 | 13 | 17-377 | 76 | | | | | | | | | | | | | | | | |
| potato_boil | 0.13 | 0.05 | 0.02 | 0.02 | 13-495 | 50 | 13-490 | 25 | 13-491 | 25 | | | | | | | | | | | | | | | | |
| potato_boil_marg | 71.05 | 33.25 | 22.37 | 10.68 | 12-503 | 25 | 12-504 | 25 | 17-685 | 25 | 17-661 | 25 | | | | | | | | | | | | | | |
| potato_fried | 7.50 | 1.74 | 3.34 | 2.01 | 13-487 | 40 | 13-489 | 19 | 17-041 | 1 | 13-486 | 20 | 13-485 | 20 | | | | | | | | | | | | |
| poultry_friedbcrumb_noskin | 12.91 | 2.97 | 6.64 | 2.53 | 18-500 | 90 | 18-503 | 10 | | | | | | | | | | | | | | | | | | |
| poultry_friedbcrumb_withskin | 12.91 | 2.97 | 6.64 | 2.53 | 18-500 | 90 | 18-503 | 10 | | | | | | | | | | | | | | | | | | |
| poultry_noskin | 4.42 | 0.89 | 2.06 | 1.24 | 18-289 | 43.875 | 18-290 | 43.875 | 17-045 | 0.9375 | 17-038 | 0.9375 | 17-041 | 0.375 | 18-361 | 10 | | | | | | | | | | |
| poultry_withskin | 7.98 | 1.90 | 3.77 | 1.84 | 18-289 | 39.375 | 18-290 | 40.875 | 18-332 | 7.5 | 17-045 | 0.9375 | 17-038 | 0.9375 | 17-041 | 0.375 | 18-361 | 9 | 18-351 | 1 | | | | | | |
| rice_brown | 1.03 | 0.20 | 0.36 | 0.33 | 11-869 | 33.4 | 11-867 | 66.6 | | | | | | | | | | | | | | | | | | |
| rice_white | 0.50 | 0.11 | 0.12 | 0.17 | 11-862 | 33.4 | 11-858 | 33.3 | 11-864 | 33.3 | | | | | | | | | | | | | | | | |
| salad_dressing | 39.96 | 3.41 | 25.09 | 9.61 | 17-041 | 30 | 17-339 | 50 | 17-063 | 10 | 17-038 | 10 | | | | | | | | | | | | | | |
| sauce_brown | 0.10 | 0.00 | 0.00 | 0.05 | 17-706 | 50 | 17-705 | 50 | | | | | | | | | | | | | | | | | | |
| sauce_cheese_chol | 4.97 | 3.05 | 1.29 | 0.17 | 11-886 | 2.5 | 17-685 | 2.5 | 12-346 | 7.5 | 12-307 | 87.5 | | | | | | | | | | | | | | |
| sauce_cheese_dontknow | 6.28 | 3.92 | 1.62 | 0.20 | 11-886 | 2.5 | 17-685 | 2.5 | 12-346 | 7.5 | 12-313 | 52.5 | 12-307 | 17.5 | 12-596 | 17.5 | | | | | | | | | | |
| sauce_cheese_goatsheep | 8.86 | 5.54 | 2.29 | 0.38 | 11-886 | 2.5 | 17-685 | 2.5 | 12-346 | 7.5 | 12-328 | 43.75 | 12-329 | 43.75 | | | | | | | | | | | | |

**Table S1.** Individual FA and major nutrient calculation in the individual FA dataset (MCW + USDA). USDA food codes are highlighted in red.

| Food item | Grams per 100g of food item[1] | | | | Codes for foods from MCW and USDA and the fraction (%) assigned to each code[2] | | | | | | | | | | | | | | | | | | | | | |
|---|---|---|---|---|---|---|---|---|---|---|---|---|---|---|---|---|---|---|---|---|---|---|---|---|---|---|
| | Total fat | Total SFAs | Total MUFAs | Total PUFAs | Code 1 | Fraction 1 | Code 2 | Fraction 2 | Code 3 | Fraction 3 | Code 4 | Fraction 4 | Code 5 | Fraction 5 | Code 6 | Fraction 6 | Code 7 | Fraction 7 | Code 8 | Fraction 8 | Code 9 | Fraction 9 | Code 10 | Fraction 10 | Code 11 | Fraction 11 |
| sauce_cheese_other | 6.04 | 3.55 | 1.71 | 0.26 | 11-886 | 2.5 | 17-685 | 2.5 | 12-346 | 7.5 | 12-313 | 23.625 | 11350000 | 35 | 11114330 | 28.875 | | | | | | | | | | |
| sauce_cheese_powdered | 4.97 | 3.05 | 1.29 | 0.17 | 11-886 | 2.5 | 17-685 | 2.5 | 12-346 | 7.5 | 12-307 | 87.5 | | | | | | | | | | | | | | |
| sauce_cheese_riceoatveg | 5.29 | 2.07 | 1.57 | 1.29 | 11-886 | 2.5 | 12-503 | 2.5 | 12-346 | 7.5 | 12-524 | 43.75 | 11350000 | 43.75 | | | | | | | | | | | | |
| sauce_cheese_semi | 6.20 | 3.88 | 1.59 | 0.19 | 11-886 | 2.5 | 17-685 | 2.5 | 12-346 | 7.5 | 12-313 | 87.5 | | | | | | | | | | | | | | |
| sauce_cheese_skimmed | 4.97 | 3.05 | 1.29 | 0.17 | 11-886 | 2.5 | 17-685 | 2.5 | 12-346 | 7.5 | 12-307 | 87.5 | | | | | | | | | | | | | | |
| sauce_cheese_soya_ca | 5.53 | 2.18 | 1.44 | 1.68 | 11-886 | 2.5 | 12-503 | 2.5 | 12-346 | 7.5 | 12-524 | 87.5 | | | | | | | | | | | | | | |
| sauce_cheese_soya_noca | 5.53 | 2.18 | 1.44 | 1.68 | 11-886 | 2.5 | 12-503 | 2.5 | 12-346 | 7.5 | 12-524 | 87.5 | | | | | | | | | | | | | | |
| sauce_cheese_whole | 7.86 | 4.94 | 2.08 | 0.24 | 11-886 | 2.5 | 17-685 | 2.5 | 12-346 | 7.5 | 12-596 | 87.5 | | | | | | | | | | | | | | |
| sauce_gravy | 6.06 | 3.09 | 2.38 | 0.20 | 28501010 | 95 | 17-487 | 5 | | | | | | | | | | | | | | | | | | |
| sauce_ketchup | 0.10 | 0.00 | 0.00 | 0.00 | 17-709 | 100 | | | | | | | | | | | | | | | | | | | | |
| sauce_tomato | 3.38 | 0.48 | 1.64 | 1.13 | 17-619 | 75 | 17-621 | 25 | | | | | | | | | | | | | | | | | | |
| sauce_white_chol | 2.46 | 1.46 | 0.60 | 0.10 | 11-886 | 10 | 17-685 | 2.5 | 12-307 | 87.5 | | | | | | | | | | | | | | | | |
| sauce_white_dontknow | 3.77 | 2.33 | 0.93 | 0.13 | 11-886 | 10 | 17-685 | 2.5 | 12-313 | 52.5 | 12-307 | 17.5 | 12-596 | 17.5 | | | | | | | | | | | | |
| sauce_white_goatsheep | 6.35 | 3.95 | 1.60 | 0.31 | 11-886 | 10 | 17-685 | 2.5 | 12-328 | 43.75 | 12-329 | 43.75 | | | | | | | | | | | | | | |
| sauce_white_other | 3.53 | 1.96 | 1.02 | 0.20 | 11-886 | 10 | 17-685 | 2.5 | 12-313 | 23.625 | 11350000 | 35 | 11114330 | 28.875 | | | | | | | | | | | | |
| sauce_white_powdered | 2.46 | 1.46 | 0.60 | 0.10 | 11-886 | 10 | 17-685 | 2.5 | 12-307 | 87.5 | | | | | | | | | | | | | | | | |
| sauce_white_riceoatveg | 2.78 | 0.48 | 0.88 | 1.23 | 11-886 | 10 | 12-503 | 2.5 | 12-524 | 43.75 | 11350000 | 43.75 | | | | | | | | | | | | | | |
| sauce_white_semi | 3.68 | 2.28 | 0.90 | 0.13 | 11-886 | 10 | 17-685 | 2.5 | 12-313 | 87.5 | | | | | | | | | | | | | | | | |
| sauce_white_skimmed | 2.46 | 1.46 | 0.60 | 0.10 | 11-886 | 10 | 17-685 | 2.5 | 12-307 | 87.5 | | | | | | | | | | | | | | | | |
| sauce_white_soya_ca | 3.02 | 0.58 | 0.75 | 1.62 | 11-886 | 10 | 12-503 | 2.5 | 12-524 | 87.5 | | | | | | | | | | | | | | | | |
| sauce_white_soya_noca | 3.02 | 0.58 | 0.75 | 1.62 | 11-886 | 10 | 12-503 | 2.5 | 12-524 | 87.5 | | | | | | | | | | | | | | | | |
| sauce_white_whole | 5.35 | 3.35 | 1.39 | 0.17 | 11-886 | 10 | 17-685 | 2.5 | 12-596 | 87.5 | | | | | | | | | | | | | | | | |
| scone | 12.30 | 6.37 | 3.54 | 1.19 | 11-827 | 100 | | | | | | | | | | | | | | | | | | | | |
| scone_gf | 12.30 | 6.37 | 3.54 | 1.19 | 11-827 | 100 | | | | | | | | | | | | | | | | | | | | |
| single_crust | 36.02 | 14.83 | 14.40 | 4.94 | 11-854 | 40 | 11-853 | 60 | | | | | | | | | | | | | | | | | | |
| smoothie_dairy | 0.73 | 0.43 | 0.16 | 0.02 | 12-379 | 25 | 12-313 | 25 | 17-747 | 50 | | | | | | | | | | | | | | | | |
| smoothie_fruit | 0.10 | 0.00 | 0.00 | 0.00 | 17-747 | 100 | | | | | | | | | | | | | | | | | | | | |
| snack_cheesybis | 28.10 | 11.67 | 12.20 | 2.62 | 11-821 | 100 | | | | | | | | | | | | | | | | | | | | |
| snack_crisps | 30.65 | 3.21 | 17.30 | 9.33 | 71201015 | 50 | 17-643 | 25 | 17-644 | 25 | | | | | | | | | | | | | | | | |
| snack_olives | 12.07 | 1.60 | 8.92 | 1.03 | 75510000 | 100 | | | | | | | | | | | | | | | | | | | | |
| snack_saltednuts | 48.33 | 7.05 | 29.13 | 9.90 | 42104200 | 50 | 14-870 | 25 | 42114130 | 25 | | | | | | | | | | | | | | | | |
| snack_saltedpeanuts | 46.00 | 8.66 | 22.03 | 13.10 | 14-877 | 100 | | | | | | | | | | | | | | | | | | | | |
| snack_savourybis | 20.83 | 1.90 | 12.38 | 5.19 | 17-645 | 67 | 11-822 | 33 | | | | | | | | | | | | | | | | | | |
| snack_seeds | 49.55 | 7.72 | 13.63 | 25.75 | 14-844 | 25 | 14-845 | 25 | 13-849 | 25 | 14-842 | 25 | | | | | | | | | | | | | | |
| snack_svyother | 27.99 | 3.91 | 13.95 | 9.04 | 17-764 | 10 | 17-645 | 40 | 71201015 | 30 | 11-826 | 20 | | | | | | | | | | | | | | |
| snack_swtother | 26.26 | 12.79 | 9.59 | 2.49 | 11-819 | 10 | 11-810 | 30 | 11-815 | 30 | 17-648 | 20 | 17-645 | 10 | | | | | | | | | | | | |
| snack_unsaltednuts | 53.87 | 6.63 | 24.96 | 19.63 | 42104200 | 25 | 14-879 | 25 | 42114130 | 25 | 14-870 | 25 | | | | | | | | | | | | | | |
| snack_unsaltedpeanuts | 46.00 | 8.66 | 22.03 | 13.10 | 14-877 | 100 | | | | | | | | | | | | | | | | | | | | |
| snackpot | 2.87 | 1.60 | 0.80 | 0.29 | 11-734 | 25 | 17-377 | 73 | 17-685 | 2 | | | | | | | | | | | | | | | | |
| soup_canned_fish | 5.88 | 3.48 | 1.43 | 0.34 | 16-373 | 12.5 | 16-263 | 6.25 | 16-384 | 6.25 | 16-390 | 12.5 | 17-377 | 20 | 13-530 | 20 | 13-489 | 16 | 17-685 | 6.5 | | | | | | |
| soup_canned_meat | 2.50 | 0.43 | 1.27 | 0.54 | 17-697 | 66.7 | 18-027 | 1.665 | 17-377 | 19.98 | 13-530 | 6.66 | 13-448 | 3.663 | 17-063 | 1.1655 | 17-041 | 0.1665 | | | | | | | | |
| soup_canned_other | 1.95 | 0.62 | 0.79 | 0.38 | 17-377 | 29.9 | 13-530 | 12.5 | 13-448 | 5 | 13-489 | 8.5 | 13-527 | 17 | 17-063 | 1.75 | 17-041 | 0.75 | 19-496 | 8 | 17-685 | 0.6 | 13-499 | 6 | 17-635 | 10 |
| soup_canned_pasta | 1.22 | 0.35 | 0.50 | 0.13 | 17-377 | 17.5 | 13-530 | 12.5 | 11-720 | 5 | 13-448 | 4.5 | 13-489 | 4.5 | 41302120 | 5 | 17-038 | 0.5 | 17-685 | 0.5 | 17-714 | 50 | | | | |
| soup_canned_pulse | 1.17 | 0.52 | 0.31 | 0.13 | 17-698 | 50 | 19-496 | 10 | 13-527 | 15 | 17-377 | 9.25 | 17-685 | 0.75 | 13-499 | 7.5 | 13-489 | 7.5 | | | | | | | | |
| soup_canned_unanswered | 1.58 | 0.18 | 0.80 | 0.41 | 17-698 | 33.3 | 17-697 | 33.3 | 17-377 | 15.03 | 13-530 | 8.35 | 13-448 | 3.34 | 13-489 | 1.67 | 13-527 | 3.34 | 17-063 | 1.169 | 17-041 | 0.501 | | | | |
| soup_canned_veg | 2.06 | 0.67 | 0.94 | 0.34 | 17-635 | 25 | 17-652 | 25 | 17-636 | 25 | 17-377 | 11.25 | 13-530 | 6.25 | 13-448 | 2.5 | 13-489 | 1.25 | 13-527 | 2.5 | 17-063 | 0.875 | 17-041 | 0.375 | | |
| soup_homemade_fish | 5.88 | 3.48 | 1.43 | 0.34 | 16-373 | 12.5 | 16-263 | 6.25 | 16-384 | 6.25 | 16-390 | 12.5 | 17-377 | 20 | 13-530 | 20 | 13-489 | 16 | 17-685 | 6.5 | | | | | | |
| soup_homemade_meat | 3.96 | 1.07 | 1.83 | 0.76 | 18-081 | 11.5 | 19-496 | 10 | 13-527 | 15 | 17-377 | 16.75 | 17-685 | 0.75 | 13-499 | 15 | 13-489 | 17.5 | 13-530 | 11 | 17-041 | 1.75 | 11-886 | 0.75 | | |
| soup_homemade_other | 3.24 | 1.88 | 0.86 | 0.23 | 17-635 | 10 | 19-496 | 8 | 13-527 | 14.5 | 17-377 | 29.9 | 17-685 | 3.1 | 13-499 | 6 | 13-489 | 9.75 | 13-530 | 12.5 | 13-448 | 6.25 | | | | |
| soup_homemade_pasta | 2.14 | 0.70 | 0.99 | 0.25 | 17-377 | 35 | 13-530 | 25 | 11-720 | 10 | 13-448 | 9 | 13-489 | 9 | 41302120 | 10 | 17-038 | 1 | 17-685 | 1 | | | | | | |
| soup_homemade_pulse | 3.23 | 1.84 | 0.85 | 0.25 | 19-496 | 10 | 13-527 | 15 | 17-377 | 21.75 | 17-685 | 3.25 | 13-499 | 12.5 | 13-489 | 12.5 | 41305000 | 25 | | | | | | | | |
| soup_homemade_unanswered | 3.57 | 2.10 | 0.92 | 0.24 | 19-496 | 6.66 | 13-527 | 11.66 | 17-377 | 29.5155 | 17-685 | 3.8345 | 13-499 | 8.325 | 13-489 | 10.83 | 13-530 | 8.35 | 13-448 | 4.175 | 41305000 | 16.65 | | | | |
| soup_homemade_veg | 2.43 | 1.20 | 0.84 | 0.23 | 17-635 | 40 | 17-652 | 20 | 17-377 | 9 | 13-530 | 5 | 13-448 | 2.5 | 13-489 | 1.5 | 13-527 | 1 | 17-685 | 1 | 17-776 | 20 | | | | |
| soup_powder | 1.34 | 0.87 | 0.31 | 0.10 | 17-653 | 10 | 17-377 | 90 | | | | | | | | | | | | | | | | | | |
| spongepuds | 15.80 | 6.21 | 5.60 | 3.07 | 11-832 | 100 | | | | | | | | | | | | | | | | | | | | |
| spongepuds_gf | 15.80 | 6.21 | 5.60 | 3.07 | 11-832 | 100 | | | | | | | | | | | | | | | | | | | | |
| spreadsauce_other | 16.17 | 2.37 | 8.55 | 4.49 | 17-314 | 27.5 | 17-654 | 15 | 17-851 | 15 | 17-626 | 27.5 | 17-707 | 15 | | | | | | | | | | | | |
| sushi | 2.22 | 0.51 | 0.75 | 0.64 | 11-862 | 46.69 | 16-360 | 10.02 | 13-448 | 2.664 | 13-524 | 2.664 | 41420010 | 4.662 | 16-362 | 33.3 | | | | | | | | | | |
| sweets | 5.48 | 3.04 | 1.77 | 0.37 | 17-101 | 50 | 17-646 | 25 | 17-647 | 25 | | | | | | | | | | | | | | | | |
| sweets_diet | 0.00 | 0.00 | 0.00 | 0.00 | 17-101 | 67 | 17-107 | 33 | | | | | | | | | | | | | | | | | | |
| tea_black | 0.00 | 0.00 | 0.00 | 0.00 | 17-167 | 50 | 17-166 | 50 | | | | | | | | | | | | | | | | | | |
| tea_black_decaf | 0.00 | 0.00 | 0.00 | 0.00 | 17-167 | 50 | 17-166 | 50 | | | | | | | | | | | | | | | | | | |
| tea_green | 0.00 | 0.00 | 0.00 | 0.00 | 17-171 | 100 | | | | | | | | | | | | | | | | | | | | |
| tea_herbal | 0.00 | 0.00 | 0.00 | 0.00 | 17-172 | 100 | | | | | | | | | | | | | | | | | | | | |
| tea_other | 0.00 | 0.00 | 0.00 | 0.00 | 17-172 | 33 | 17-167 | 33 | 17-166 | 34 | | | | | | | | | | | | | | | | |
| tea_rooibos | 0.00 | 0.00 | 0.00 | 0.00 | 17-172 | 100 | | | | | | | | | | | | | | | | | | | | |
| tea_sugar | 0.00 | 0.00 | 0.00 | 0.00 | 17-063 | 100 | | | | | | | | | | | | | | | | | | | | |
| veg_avocado | 14.66 | 2.13 | 9.80 | 1.82 | 63105010 | 100 | | | | | | | | | | | | | | | | | | | | |
| veg_bakedbeans | 0.50 | 0.09 | 0.06 | 0.27 | 13-532 | 75 | 13-535 | 25 | | | | | | | | | | | | | | | | | | |
| veg_beetroot | 0.25 | 0.03 | 0.03 | 0.11 | 13-166 | 50 | 13-633 | 50 | | | | | | | | | | | | | | | | | | |
| veg_broadbeans | 0.40 | 0.07 | 0.08 | 0.16 | 41102220 | 100 | | | | | | | | | | | | | | | | | | | | |
| veg_broccoli | 0.50 | 0.12 | 0.05 | 0.20 | 13-503 | 100 | | | | | | | | | | | | | | | | | | | | |
| veg_butternut | 0.20 | 0.00 | 0.02 | 0.10 | 13-644 | 100 | | | | | | | | | | | | | | | | | | | | |
| veg_cabbagekale | 0.37 | 0.03 | 0.02 | 0.19 | 72119210 | 66.7 | 13-540 | 33.3 | | | | | | | | | | | | | | | | | | |
| veg_carrots | 0.47 | 0.13 | 0.10 | 0.16 | 13-497 | 60 | 13-449 | 30 | 13-448 | 10 | 13-497 | | | | | | | | | | | | | | | |

**Table S1.** Individual FA and major nutrient calculation in the individual FA dataset (MCW + USDA). USDA food codes are highlighted in red.

| Food item | Grams per 100g of food item[1] | | | | Codes for foods from MCW and USDA and the fraction (%) assigned to each code[2] | | | | | | | | | | | | | | | | | | | | | |
|---|---|---|---|---|---|---|---|---|---|---|---|---|---|---|---|---|---|---|---|---|---|---|---|---|---|---|
| | Total fat | Total SFAs | Total MUFAs | Total PUFAs | Code 1 | Fraction 1 | Code 2 | Fraction 2 | Code 3 | Fraction 3 | Code 4 | Fraction 4 | Code 5 | Fraction 5 | Code 6 | Fraction 6 | Code 7 | Fraction 7 | Code 8 | Fraction 8 | Code 9 | Fraction 9 | Code 10 | Fraction 10 | Code 11 | Fraction 11 |
| veg_cauli | 0.37 | 0.06 | 0.03 | 0.18 | 75214010 | 100 | | | | | | | | | | | | | | | | | | | | |
| veg_celery | 0.10 | 0.00 | 0.03 | 0.07 | 13-636 | 100 | | | | | | | | | | | | | | | | | | | | |
| veg_cour_butter_dunno | 2.31 | 1.16 | 0.63 | 0.31 | 13-628 | 97.5 | 17-655 | 1.25 | 17-685 | 0.625 | 17-661 | 0.625 | | | | | | | | | | | | | | |
| veg_cour_butter_fat | 2.35 | 1.38 | 0.52 | 0.22 | 13-628 | 97.5 | 17-685 | 1.25 | 17-661 | 1.25 | | | | | | | | | | | | | | | | |
| veg_cour_butter_lowfat | 1.80 | 0.72 | 0.57 | 0.34 | 13-628 | 97.5 | 17-656 | 2.5 | | | | | | | | | | | | | | | | | | |
| veg_cour_butter_spread_fat | 2.27 | 0.93 | 0.74 | 0.40 | 13-628 | 97.5 | 17-655 | 2.5 | | | | | | | | | | | | | | | | | | |
| veg_cour_butter_spread_lowfat | 1.80 | 0.72 | 0.57 | 0.34 | 13-628 | 97.5 | 17-656 | 2.5 | | | | | | | | | | | | | | | | | | |
| veg_cour_fat_dunno | 2.79 | 0.24 | 1.48 | 0.88 | 13-628 | 97.5 | 17-686 | 2.5 | | | | | | | | | | | | | | | | | | |
| veg_cour_fat_none | 0.30 | 0.08 | 0.00 | 0.15 | 13-628 | 100 | | | | | | | | | | | | | | | | | | | | |
| veg_cour_fat_other | 2.77 | 1.48 | 0.83 | 0.22 | 13-628 | 97.5 | 81204000 | 1.25 | 17-487 | 1.25 | | | | | | | | | | | | | | | | |
| veg_cour_lard | 2.77 | 1.09 | 1.09 | 0.40 | 13-628 | 97.5 | 17-010 | 2.5 | | | | | | | | | | | | | | | | | | |
| veg_cour_marg_hard | 2.20 | 0.74 | 0.85 | 0.45 | 13-628 | 97.5 | 12-500 | 2.5 | | | | | | | | | | | | | | | | | | |
| veg_cour_marg_poly_chol | 1.22 | 0.29 | 0.29 | 0.52 | 13-628 | 97.5 | 12-499 | 2.5 | | | | | | | | | | | | | | | | | | |
| veg_cour_marg_poly_dunno | 1.49 | 0.35 | 0.37 | 0.65 | 13-628 | 97.5 | 12-499 | 1.25 | 12-503 | 1.25 | | | | | | | | | | | | | | | | |
| veg_cour_marg_poly_fat | 1.77 | 0.41 | 0.44 | 0.78 | 13-628 | 97.5 | 12-503 | 2.5 | | | | | | | | | | | | | | | | | | |
| veg_cour_marg_poly_lowfat | 1.22 | 0.29 | 0.29 | 0.52 | 13-628 | 97.5 | 12-499 | 2.5 | | | | | | | | | | | | | | | | | | |
| veg_cour_marg_poly_vlowfat | 1.22 | 0.29 | 0.29 | 0.52 | 13-628 | 97.5 | 12-499 | 2.5 | | | | | | | | | | | | | | | | | | |
| veg_cour_marg_soya_chol | 1.22 | 0.29 | 0.29 | 0.52 | 13-628 | 97.5 | 12-499 | 2.5 | | | | | | | | | | | | | | | | | | |
| veg_cour_marg_soya_dunno | 1.49 | 0.35 | 0.37 | 0.65 | 13-628 | 97.5 | 12-499 | 1.25 | 12-503 | 1.25 | | | | | | | | | | | | | | | | |
| veg_cour_marg_soya_fat | 1.77 | 0.41 | 0.44 | 0.78 | 13-628 | 97.5 | 12-503 | 2.5 | | | | | | | | | | | | | | | | | | |
| veg_cour_marg_soya_lowfat | 1.22 | 0.29 | 0.29 | 0.52 | 13-628 | 97.5 | 12-499 | 2.5 | | | | | | | | | | | | | | | | | | |
| veg_cour_marg_soya_vlowfat | 1.22 | 0.29 | 0.29 | 0.52 | 13-628 | 97.5 | 12-499 | 2.5 | | | | | | | | | | | | | | | | | | |
| veg_cour_oil_olive | 2.79 | 0.44 | 1.83 | 0.35 | 13-628 | 97.5 | 17-038 | 2.5 | | | | | | | | | | | | | | | | | | |
| veg_cour_oil_other | 2.48 | 0.35 | 1.00 | 0.96 | 13-628 | 97.5 | 17-033 | 0.75 | 17-040 | 0.5 | 17-686 | 0.938 | 17-377 | 0.312 | | | | | | | | | | | | |
| veg_cour_oil_rapeseed | 2.79 | 0.24 | 1.48 | 0.88 | 13-628 | 97.5 | 17-041 | 2.5 | | | | | | | | | | | | | | | | | | |
| veg_cour_oil_sunflower | 2.79 | 0.38 | 0.51 | 1.73 | 13-628 | 97.5 | 17-045 | 2.5 | | | | | | | | | | | | | | | | | | |
| veg_cour_oil_veg | 2.79 | 0.24 | 1.48 | 0.88 | 13-628 | 97.5 | 17-686 | 2.5 | | | | | | | | | | | | | | | | | | |
| veg_cour_s_dairy_chol | 1.27 | 0.32 | 0.43 | 0.39 | 13-628 | 97.5 | 12-497 | 2.5 | | | | | | | | | | | | | | | | | | |
| veg_cour_s_dairy_dunno | 1.54 | 0.40 | 0.59 | 0.42 | 13-628 | 97.5 | 12-497 | 1.25 | 12-504 | 1.25 | | | | | | | | | | | | | | | | |
| veg_cour_s_dairy_fat | 1.81 | 0.47 | 0.75 | 0.44 | 13-628 | 97.5 | 12-504 | 2.5 | | | | | | | | | | | | | | | | | | |
| veg_cour_s_dairy_lowfat | 1.27 | 0.32 | 0.43 | 0.39 | 13-628 | 97.5 | 12-497 | 2.5 | | | | | | | | | | | | | | | | | | |
| veg_cour_s_dairy_vlowfat | 1.27 | 0.32 | 0.43 | 0.39 | 13-628 | 97.5 | 12-497 | 2.5 | | | | | | | | | | | | | | | | | | |
| veg_cour_s_dunno_chol | 1.24 | 0.31 | 0.36 | 0.46 | 13-628 | 97.5 | 12-499 | 1.25 | 12-497 | 1.25 | | | | | | | | | | | | | | | | |
| veg_cour_s_dunno_dunno | 1.52 | 0.37 | 0.48 | 0.53 | 13-628 | 97.5 | 12-499 | 0.625 | 12-503 | 0.625 | 12-497 | 0.625 | 12-504 | 0.625 | | | | | | | | | | | | |
| veg_cour_s_dunno_fat | 1.79 | 0.44 | 0.60 | 0.61 | 13-628 | 97.5 | 12-503 | 1.25 | 12-504 | 1.25 | | | | | | | | | | | | | | | | |
| veg_cour_s_dunno_lowfat | 1.24 | 0.31 | 0.36 | 0.46 | 13-628 | 97.5 | 12-499 | 1.25 | 12-497 | 1.25 | | | | | | | | | | | | | | | | |
| veg_cour_s_dunno_vlowfat | 1.24 | 0.31 | 0.36 | 0.46 | 13-628 | 97.5 | 12-499 | 1.25 | 12-497 | 1.25 | | | | | | | | | | | | | | | | |
| veg_cour_s_olive_chol | 1.27 | 0.30 | 0.54 | 0.31 | 13-628 | 97.5 | 12-498 | 2.5 | | | | | | | | | | | | | | | | | | |
| veg_cour_s_olive_dunno | 1.52 | 0.35 | 0.66 | 0.37 | 13-628 | 97.5 | 12-498 | 1.25 | 12-505 | 1.25 | | | | | | | | | | | | | | | | |
| veg_cour_s_olive_fat | 1.77 | 0.41 | 0.78 | 0.43 | 13-628 | 97.5 | 12-505 | 2.5 | | | | | | | | | | | | | | | | | | |
| veg_cour_s_olive_lowfat | 1.27 | 0.30 | 0.54 | 0.31 | 13-628 | 97.5 | 12-498 | 2.5 | | | | | | | | | | | | | | | | | | |
| veg_cour_s_olive_vlowfat | 1.27 | 0.30 | 0.54 | 0.31 | 13-628 | 97.5 | 12-498 | 2.5 | | | | | | | | | | | | | | | | | | |
| veg_cucumber | 0.10 | 0.00 | 0.00 | 0.00 | 13-245 | 100 | | | | | | | | | | | | | | | | | | | | |
| veg_garlic | 0.50 | 0.09 | 0.01 | 0.25 | 75111500 | 100 | | | | | | | | | | | | | | | | | | | | |
| veg_greenbeans | 0.35 | 0.07 | 0.00 | 0.21 | 13-515 | 75 | 13-654 | 25 | | | | | | | | | | | | | | | | | | |
| veg_leek_butter_dunno | 1.21 | 0.57 | 0.32 | 0.19 | 13-625 | 98.75 | 17-655 | 0.625 | 17-685 | 0.3125 | 17-661 | 0.3125 | | | | | | | | | | | | | | |
| veg_leek_butter_fat | 1.23 | 0.68 | 0.26 | 0.14 | 13-625 | 98.75 | 17-685 | 0.625 | 17-661 | 0.625 | | | | | | | | | | | | | | | | |
| veg_leek_butter_lowfat | 0.95 | 0.35 | 0.28 | 0.21 | 13-625 | 98.75 | 17-656 | 1.25 | | | | | | | | | | | | | | | | | | |
| veg_leek_butter_spread_fat | 1.19 | 0.46 | 0.37 | 0.23 | 13-625 | 98.75 | 17-655 | 1.25 | | | | | | | | | | | | | | | | | | |
| veg_leek_butter_spread_lowfat | 0.95 | 0.35 | 0.28 | 0.21 | 13-625 | 98.75 | 17-656 | 1.25 | | | | | | | | | | | | | | | | | | |
| veg_leek_fat_dunno | 1.45 | 0.11 | 0.74 | 0.47 | 13-625 | 98.75 | 17-686 | 1.25 | | | | | | | | | | | | | | | | | | |
| veg_leek_fat_none | 0.20 | 0.03 | 0.00 | 0.11 | 13-625 | 100 | | | | | | | | | | | | | | | | | | | | |
| veg_leek_fat_other | 1.44 | 0.73 | 0.42 | 0.15 | 13-625 | 98.75 | 81204000 | 0.625 | 17-487 | 0.625 | | | | | | | | | | | | | | | | |
| veg_leek_lard | 1.44 | 0.53 | 0.54 | 0.23 | 13-625 | 98.75 | 17-010 | 1.25 | | | | | | | | | | | | | | | | | | |
| veg_leek_marg_hard | 1.15 | 0.36 | 0.43 | 0.26 | 13-625 | 98.75 | 12-500 | 1.25 | | | | | | | | | | | | | | | | | | |
| veg_leek_marg_poly_chol | 0.66 | 0.14 | 0.14 | 0.30 | 13-625 | 98.75 | 12-499 | 1.25 | | | | | | | | | | | | | | | | | | |
| veg_leek_marg_poly_dunno | 0.80 | 0.17 | 0.18 | 0.36 | 13-625 | 98.75 | 12-499 | 0.625 | 12-503 | 0.625 | | | | | | | | | | | | | | | | |
| veg_leek_marg_poly_fat | 0.94 | 0.19 | 0.22 | 0.42 | 13-625 | 98.75 | 12-503 | 1.25 | | | | | | | | | | | | | | | | | | |
| veg_leek_marg_poly_lowfat | 0.66 | 0.14 | 0.14 | 0.30 | 13-625 | 98.75 | 12-499 | 1.25 | | | | | | | | | | | | | | | | | | |
| veg_leek_marg_poly_vlowfat | 0.66 | 0.14 | 0.14 | 0.30 | 13-625 | 98.75 | 12-499 | 1.25 | | | | | | | | | | | | | | | | | | |
| veg_leek_marg_soya_chol | 0.66 | 0.14 | 0.14 | 0.30 | 13-625 | 98.75 | 12-499 | 1.25 | | | | | | | | | | | | | | | | | | |
| veg_leek_marg_soya_dunno | 0.80 | 0.17 | 0.18 | 0.36 | 13-625 | 98.75 | 12-499 | 0.625 | 12-503 | 0.625 | | | | | | | | | | | | | | | | |
| veg_leek_marg_soya_fat | 0.94 | 0.19 | 0.22 | 0.42 | 13-625 | 98.75 | 12-503 | 1.25 | | | | | | | | | | | | | | | | | | |
| veg_leek_marg_soya_lowfat | 0.66 | 0.14 | 0.14 | 0.30 | 13-625 | 98.75 | 12-499 | 1.25 | | | | | | | | | | | | | | | | | | |
| veg_leek_marg_soya_vlowfat | 0.66 | 0.14 | 0.14 | 0.30 | 13-625 | 98.75 | 12-499 | 1.25 | | | | | | | | | | | | | | | | | | |
| veg_leek_oil_olive | 1.45 | 0.21 | 0.91 | 0.21 | 13-625 | 98.75 | 17-038 | 1.25 | | | | | | | | | | | | | | | | | | |
| veg_leek_oil_other | 1.30 | 0.17 | 0.50 | 0.52 | 13-625 | 98.75 | 17-033 | 0.38 | 17-040 | 0.25 | 17-686 | 0.47 | 17-377 | 0.15 | | | | | | | | | | | | |
| veg_leek_oil_rapeseed | 1.45 | 0.11 | 0.74 | 0.47 | 13-625 | 98.75 | 17-041 | 1.25 | | | | | | | | | | | | | | | | | | |
| veg_leek_oil_sunflower | 1.45 | 0.18 | 0.26 | 0.90 | 13-625 | 98.75 | 17-045 | 1.25 | | | | | | | | | | | | | | | | | | |
| veg_leek_oil_veg | 1.45 | 0.11 | 0.74 | 0.47 | 13-625 | 98.75 | 17-686 | 1.25 | | | | | | | | | | | | | | | | | | |
| veg_leek_spread_dairy_chol | 0.69 | 0.15 | 0.22 | 0.23 | 13-625 | 98.75 | 12-497 | 1.25 | | | | | | | | | | | | | | | | | | |
| veg_leek_spread_dairy_dunno | 0.82 | 0.19 | 0.30 | 0.24 | 13-625 | 98.75 | 12-497 | 0.625 | 12-504 | 0.625 | | | | | | | | | | | | | | | | |
| veg_leek_spread_dairy_fat | 0.96 | 0.22 | 0.37 | 0.26 | 13-625 | 98.75 | 12-504 | 1.25 | | | | | | | | | | | | | | | | | | |
| veg_leek_spread_dairy_lowfat | 0.69 | 0.15 | 0.22 | 0.23 | 13-625 | 98.75 | 12-497 | 1.25 | | | | | | | | | | | | | | | | | | |
| veg_leek_spread_dairy_vlowfat | 0.69 | 0.15 | 0.22 | 0.23 | 13-625 | 98.75 | 12-497 | 1.25 | | | | | | | | | | | | | | | | | | |

**Table S1.** Individual FA and major nutrient calculation in the individual FA dataset (MCW + USDA). USDA food codes are highlighted in red.

| Food item | Grams per 100g of food item[1] | | | | Codes for foods from MCW and USDA and the fraction (%) assigned to each code[2] | | | | | | | | | | | | | | | | | | | | | |
|---|---|---|---|---|---|---|---|---|---|---|---|---|---|---|---|---|---|---|---|---|---|---|---|---|---|---|
| | Total fat | Total SFAs | Total MUFAs | Total PUFAs | Code 1 | Fraction 1 | Code 2 | Fraction 2 | Code 3 | Fraction 3 | Code 4 | Fraction 4 | Code 5 | Fraction 5 | Code 6 | Fraction 6 | Code 7 | Fraction 7 | Code 8 | Fraction 8 | Code 9 | Fraction 9 | Code 10 | Fraction 10 | Code 11 | Fraction 11 |
| veg_leek_spread_dunno_chol | 0.67 | 0.14 | 0.18 | 0.26 | 13-625 | 98.75 | 12-499 | 0.625 | 12-497 | 0.625 | | | | | | | | | | | | | | | | |
| veg_leek_spread_dunno_dunno | 0.81 | 0.18 | 0.24 | 0.30 | 13-625 | 98.75 | 12-499 | 0.3125 | 12-503 | 0.3125 | 12-497 | 0.3125 | 12-504 | 0.3125 | | | | | | | | | | | | |
| veg_leek_spread_dunno_fat | 0.95 | 0.21 | 0.30 | 0.34 | 13-625 | 98.75 | 12-503 | 0.625 | 12-504 | 0.625 | | | | | | | | | | | | | | | | |
| veg_leek_spread_dunno_lowfat | 0.67 | 0.14 | 0.18 | 0.26 | 13-625 | 98.75 | 12-497 | 0.625 | 12-499 | 0.625 | | | | | | | | | | | | | | | | |
| veg_leek_spread_dunno_vlowfat | 0.67 | 0.14 | 0.18 | 0.26 | 13-625 | 98.75 | 12-497 | 0.625 | 12-499 | 0.625 | | | | | | | | | | | | | | | | |
| veg_leek_spread_olive_chol | 0.68 | 0.14 | 0.27 | 0.19 | 13-625 | 98.75 | 12-498 | 1.25 | | | | | | | | | | | | | | | | | | |
| veg_leek_spread_olive_dunno | 0.81 | 0.17 | 0.33 | 0.22 | 13-625 | 98.75 | 12-498 | 0.625 | 12-505 | 0.625 | | | | | | | | | | | | | | | | |
| veg_leek_spread_olive_fat | 0.94 | 0.19 | 0.39 | 0.25 | 13-625 | 98.75 | 12-505 | 1.25 | | | | | | | | | | | | | | | | | | |
| veg_leek_spread_olive_lowfat | 0.68 | 0.14 | 0.27 | 0.19 | 13-625 | 98.75 | 12-498 | 1.25 | | | | | | | | | | | | | | | | | | |
| veg_leek_spread_olive_vlowfat | 0.68 | 0.14 | 0.27 | 0.19 | 13-625 | 98.75 | 12-498 | 1.25 | | | | | | | | | | | | | | | | | | |
| veg_lettuce | 0.10 | 0.03 | 0.00 | 0.08 | 13-520 | 100 | | | | | | | | | | | | | | | | | | | | |
| veg_mixed | 0.50 | 0.00 | 0.00 | 0.00 | 13-543 | 100 | | | | | | | | | | | | | | | | | | | | |
| veg_mush_butter_dunno | 2.31 | 1.16 | 0.63 | 0.33 | 13-506 | 97.5 | 17-655 | 1.25 | 17-685 | 0.625 | 17-661 | 0.625 | | | | | | | | | | | | | | |
| veg_mush_butter_fat | 2.35 | 1.38 | 0.52 | 0.24 | 13-506 | 97.5 | 17-685 | 1.25 | 17-661 | 1.25 | | | | | | | | | | | | | | | | |
| veg_mush_butter_lowfat | 1.80 | 0.72 | 0.57 | 0.36 | 13-506 | 97.5 | 17-656 | 2.5 | | | | | | | | | | | | | | | | | | |
| veg_mush_butter_spread_fat | 2.27 | 0.93 | 0.74 | 0.42 | 13-506 | 97.5 | 17-655 | 2.5 | | | | | | | | | | | | | | | | | | |
| veg_mush_butter_spread_lowfat | 1.80 | 0.72 | 0.57 | 0.36 | 13-506 | 97.5 | 17-656 | 2.5 | | | | | | | | | | | | | | | | | | |
| veg_mush_fat_dunno | 2.79 | 0.24 | 1.48 | 0.90 | 13-506 | 97.5 | 17-686 | 2.5 | | | | | | | | | | | | | | | | | | |
| veg_mush_fat_none | 0.30 | 0.08 | 0.00 | 0.17 | 13-506 | 100 | | | | | | | | | | | | | | | | | | | | |
| veg_mush_fat_other | 2.77 | 1.48 | 0.83 | 0.24 | 13-506 | 97.5 | 81204000 | 1.25 | 17-487 | 1.25 | | | | | | | | | | | | | | | | |
| veg_mush_lard | 2.77 | 1.09 | 1.09 | 0.42 | 13-506 | 97.5 | 17-010 | 2.5 | | | | | | | | | | | | | | | | | | |
| veg_mush_marg_hard | 2.20 | 0.74 | 0.85 | 0.47 | 13-506 | 97.5 | 12-500 | 2.5 | | | | | | | | | | | | | | | | | | |
| veg_mush_marg_poly_chol | 1.22 | 0.29 | 0.29 | 0.54 | 13-506 | 97.5 | 12-499 | 2.5 | | | | | | | | | | | | | | | | | | |
| veg_mush_marg_poly_dunno | 1.49 | 0.35 | 0.37 | 0.67 | 13-506 | 97.5 | 12-499 | 1.25 | 12-503 | 1.25 | | | | | | | | | | | | | | | | |
| veg_mush_marg_poly_fat | 1.77 | 0.41 | 0.44 | 0.80 | 13-506 | 97.5 | 12-503 | 2.5 | | | | | | | | | | | | | | | | | | |
| veg_mush_marg_poly_lowfat | 1.22 | 0.29 | 0.29 | 0.54 | 13-506 | 97.5 | 12-499 | 2.5 | | | | | | | | | | | | | | | | | | |
| veg_mush_marg_poly_vlowfat | 1.22 | 0.29 | 0.29 | 0.54 | 13-506 | 97.5 | 12-499 | 2.5 | | | | | | | | | | | | | | | | | | |
| veg_mush_marg_soya_chol | 1.22 | 0.29 | 0.29 | 0.54 | 13-506 | 97.5 | 12-499 | 2.5 | | | | | | | | | | | | | | | | | | |
| veg_mush_marg_soya_dunno | 1.49 | 0.35 | 0.37 | 0.67 | 13-506 | 97.5 | 12-499 | 1.25 | 12-503 | 1.25 | | | | | | | | | | | | | | | | |
| veg_mush_marg_soya_fat | 1.77 | 0.41 | 0.44 | 0.80 | 13-506 | 97.5 | 12-503 | 2.5 | | | | | | | | | | | | | | | | | | |
| veg_mush_marg_soya_lowfat | 1.22 | 0.29 | 0.29 | 0.54 | 13-506 | 97.5 | 12-499 | 2.5 | | | | | | | | | | | | | | | | | | |
| veg_mush_marg_soya_vlowfat | 1.22 | 0.29 | 0.29 | 0.54 | 13-506 | 97.5 | 12-499 | 2.5 | | | | | | | | | | | | | | | | | | |
| veg_mush_oil_olive | 2.79 | 0.44 | 1.83 | 0.37 | 13-506 | 97.5 | 17-038 | 2.5 | | | | | | | | | | | | | | | | | | |
| veg_mush_oil_other | 2.48 | 0.35 | 1.00 | 0.98 | 13-506 | 97.5 | 17-033 | 0.75 | 17-040 | 0.5 | 17-686 | 0.938 | 17-377 | 0.312 | | | | | | | | | | | | |
| veg_mush_oil_rapeseed | 2.79 | 0.24 | 1.48 | 0.90 | 13-506 | 97.5 | 17-041 | 2.5 | | | | | | | | | | | | | | | | | | |
| veg_mush_oil_sunflower | 2.79 | 0.38 | 0.51 | 1.75 | 13-506 | 97.5 | 17-045 | 2.5 | | | | | | | | | | | | | | | | | | |
| veg_mush_oil_veg | 2.79 | 0.24 | 1.48 | 0.90 | 13-506 | 97.5 | 17-686 | 2.5 | | | | | | | | | | | | | | | | | | |
| veg_mush_s_dairy_chol | 1.27 | 0.32 | 0.43 | 0.41 | 13-506 | 97.5 | 12-497 | 2.5 | | | | | | | | | | | | | | | | | | |
| veg_mush_s_dairy_dunno | 1.54 | 0.40 | 0.59 | 0.44 | 13-506 | 97.5 | 12-497 | 1.25 | 12-504 | 1.25 | | | | | | | | | | | | | | | | |
| veg_mush_s_dairy_fat | 1.81 | 0.47 | 0.75 | 0.46 | 13-506 | 97.5 | 12-504 | 2.5 | | | | | | | | | | | | | | | | | | |
| veg_mush_s_dairy_lowfat | 1.27 | 0.32 | 0.43 | 0.41 | 13-506 | 97.5 | 12-497 | 2.5 | | | | | | | | | | | | | | | | | | |
| veg_mush_s_dairy_vlowfat | 1.27 | 0.32 | 0.43 | 0.41 | 13-506 | 97.5 | 12-497 | 2.5 | | | | | | | | | | | | | | | | | | |
| veg_mush_s_dunno_chol | 1.24 | 0.31 | 0.36 | 0.48 | 13-506 | 97.5 | 12-499 | 1.25 | 12-497 | 1.25 | | | | | | | | | | | | | | | | |
| veg_mush_s_dunno_dunno | 1.52 | 0.37 | 0.48 | 0.55 | 13-506 | 97.5 | 12-499 | 0.625 | 12-503 | 0.625 | 12-497 | 0.625 | 12-504 | 0.625 | | | | | | | | | | | | |
| veg_mush_s_dunno_fat | 1.79 | 0.44 | 0.60 | 0.63 | 13-506 | 97.5 | 12-503 | 1.25 | 12-504 | 1.25 | | | | | | | | | | | | | | | | |
| veg_mush_s_dunno_lowfat | 1.24 | 0.31 | 0.36 | 0.48 | 13-506 | 97.5 | 12-499 | 1.25 | 12-497 | 1.25 | | | | | | | | | | | | | | | | |
| veg_mush_s_dunno_vlowfat | 1.24 | 0.31 | 0.36 | 0.48 | 13-506 | 97.5 | 12-499 | 1.25 | 12-497 | 1.25 | | | | | | | | | | | | | | | | |
| veg_mush_s_olive_chol | 1.27 | 0.30 | 0.54 | 0.32 | 13-506 | 97.5 | 12-498 | 2.5 | | | | | | | | | | | | | | | | | | |
| veg_mush_s_olive_dunno | 1.52 | 0.35 | 0.66 | 0.39 | 13-506 | 97.5 | 12-498 | 1.25 | 12-505 | 1.25 | | | | | | | | | | | | | | | | |
| veg_mush_s_olive_fat | 1.77 | 0.41 | 0.78 | 0.45 | 13-506 | 97.5 | 12-505 | 2.5 | | | | | | | | | | | | | | | | | | |
| veg_mush_s_olive_lowfat | 1.27 | 0.30 | 0.54 | 0.32 | 13-506 | 97.5 | 12-498 | 2.5 | | | | | | | | | | | | | | | | | | |
| veg_mush_s_olive_vlowfat | 1.27 | 0.30 | 0.54 | 0.32 | 13-506 | 97.5 | 12-498 | 2.5 | | | | | | | | | | | | | | | | | | |
| veg_mxtr_butter_dunno | 4.51 | 2.16 | 1.26 | 0.32 | 13-543 | 95 | 17-655 | 2.5 | 17-685 | 1.25 | 17-661 | 1.25 | | | | | | | | | | | | | | |
| veg_mxtr_butter_fat | 4.59 | 2.60 | 1.05 | 0.14 | 13-543 | 95 | 17-685 | 2.5 | 17-661 | 2.5 | | | | | | | | | | | | | | | | |
| veg_mxtr_butter_lowfat | 3.49 | 1.29 | 1.13 | 0.39 | 13-543 | 95 | 17-656 | 5 | | | | | | | | | | | | | | | | | | |
| veg_mxtr_butter_spread_fat | 4.43 | 1.71 | 1.48 | 0.50 | 13-543 | 95 | 17-655 | 5 | | | | | | | | | | | | | | | | | | |
| veg_mxtr_butter_spread_lowfat | 3.49 | 1.29 | 1.13 | 0.39 | 13-543 | 95 | 17-656 | 5 | | | | | | | | | | | | | | | | | | |
| veg_mxtr_fat_dunno | 5.47 | 0.33 | 2.97 | 1.47 | 13-543 | 95 | 17-686 | 5 | | | | | | | | | | | | | | | | | | |
| veg_mxtr_fat_none | 0.50 | 0.00 | 0.00 | 0.00 | 13-543 | 100 | | | | | | | | | | | | | | | | | | | | |
| veg_mxtr_fat_other | 5.44 | 2.81 | 1.67 | 0.15 | 13-543 | 95 | 81204000 | 2.5 | 17-487 | 2.5 | | | | | | | | | | | | | | | | |
| veg_mxtr_lard | 5.43 | 2.02 | 2.17 | 0.50 | 13-543 | 95 | 17-010 | 5 | | | | | | | | | | | | | | | | | | |
| veg_mxtr_marg_hard | 4.30 | 1.32 | 1.70 | 0.61 | 13-543 | 95 | 12-500 | 5 | | | | | | | | | | | | | | | | | | |
| veg_mxtr_marg_poly_chol | 2.32 | 0.43 | 0.58 | 0.75 | 13-543 | 95 | 12-499 | 5 | | | | | | | | | | | | | | | | | | |
| veg_mxtr_marg_poly_dunno | 2.88 | 0.54 | 0.73 | 1.00 | 13-543 | 95 | 12-499 | 2.5 | 12-503 | 2.5 | | | | | | | | | | | | | | | | |
| veg_mxtr_marg_poly_fat | 3.44 | 0.66 | 0.89 | 1.26 | 13-543 | 95 | 12-503 | 5 | | | | | | | | | | | | | | | | | | |
| veg_mxtr_marg_poly_lowfat | 2.32 | 0.43 | 0.58 | 0.75 | 13-543 | 95 | 12-499 | 5 | | | | | | | | | | | | | | | | | | |
| veg_mxtr_marg_poly_vlowfat | 2.32 | 0.43 | 0.58 | 0.75 | 13-543 | 95 | 12-499 | 5 | | | | | | | | | | | | | | | | | | |
| veg_mxtr_marg_soya_chol | 2.32 | 0.43 | 0.58 | 0.75 | 13-543 | 95 | 12-499 | 5 | | | | | | | | | | | | | | | | | | |
| veg_mxtr_marg_soya_dunno | 2.88 | 0.54 | 0.73 | 1.00 | 13-543 | 95 | 12-499 | 2.5 | 12-503 | 2.5 | | | | | | | | | | | | | | | | |
| veg_mxtr_marg_soya_fat | 3.44 | 0.66 | 0.89 | 1.26 | 13-543 | 95 | 12-503 | 5 | | | | | | | | | | | | | | | | | | |
| veg_mxtr_marg_soya_lowfat | 2.32 | 0.43 | 0.58 | 0.75 | 13-543 | 95 | 12-499 | 5 | | | | | | | | | | | | | | | | | | |
| veg_mxtr_marg_soya_vlowfat | 2.32 | 0.43 | 0.58 | 0.75 | 13-543 | 95 | 12-499 | 5 | | | | | | | | | | | | | | | | | | |
| veg_mxtr_oil_olive | 5.47 | 0.72 | 3.65 | 0.41 | 13-543 | 95 | 17-038 | 5 | | | | | | | | | | | | | | | | | | |
| veg_mxtr_oil_other | 4.85 | 0.54 | 2.00 | 1.63 | 13-543 | 95 | 17-033 | 1.5 | 17-040 | 1 | 17-686 | 1.875 | 17-377 | 0.625 | | | | | | | | | | | | |
| veg_mxtr_oil_rapeseed | 5.47 | 0.33 | 2.97 | 1.47 | 13-543 | 95 | 17-041 | 5 | | | | | | | | | | | | | | | | | | |

**Table S1.** Individual FA and major nutrient calculation in the individual FA dataset (MCW + USDA). USDA food codes are highlighted in red.

| Food item | Grams per 100g of food item[1] | | | | Codes for foods from MCW and USDA and the fraction (%) assigned to each code[2] | | | | | | | | | | | | | | | | | | | | | |
|---|---|---|---|---|---|---|---|---|---|---|---|---|---|---|---|---|---|---|---|---|---|---|---|---|---|---|
| | Total fat | Total SFAs | Total MUFAs | Total PUFAs | Code 1 | Fraction 1 | Code 2 | Fraction 2 | Code 3 | Fraction 3 | Code 4 | Fraction 4 | Code 5 | Fraction 5 | Code 6 | Fraction 6 | Code 7 | Fraction 7 | Code 8 | Fraction 8 | Code 9 | Fraction 9 | Code 10 | Fraction 10 | Code 11 | Fraction 11 |
| veg_mxtr_oil_sunflower | 5.47 | 0.60 | 1.03 | 3.17 | 13-543 | 95 | 17-045 | 5 | | | | | | | | | | | | | | | | | | |
| veg_mxtr_oil_veg | 5.47 | 0.33 | 2.97 | 1.47 | 13-543 | 95 | 17-686 | 5 | | | | | | | | | | | | | | | | | | |
| veg_mxtr_s_dairy_chol | 2.43 | 0.49 | 0.87 | 0.49 | 13-543 | 95 | 12-497 | 5 | | | | | | | | | | | | | | | | | | |
| veg_mxtr_s_dairy_dunno | 2.97 | 0.63 | 1.18 | 0.54 | 13-543 | 95 | 12-497 | 2.5 | 12-504 | 2.5 | | | | | | | | | | | | | | | | |
| veg_mxtr_s_dairy_fat | 3.51 | 0.78 | 1.50 | 0.60 | 13-543 | 95 | 12-504 | 5 | | | | | | | | | | | | | | | | | | |
| veg_mxtr_s_dairy_lowfat | 2.43 | 0.49 | 0.87 | 0.49 | 13-543 | 95 | 12-497 | 5 | | | | | | | | | | | | | | | | | | |
| veg_mxtr_s_dairy_vlowfat | 2.43 | 0.49 | 0.87 | 0.49 | 13-543 | 95 | 12-497 | 5 | | | | | | | | | | | | | | | | | | |
| veg_mxtr_s_dunno_chol | 2.37 | 0.46 | 0.72 | 0.62 | 13-543 | 95 | 12-499 | 2.5 | 12-497 | 2.5 | | | | | | | | | | | | | | | | |
| veg_mxtr_s_dunno_dunno | 2.92 | 0.59 | 0.96 | 0.77 | 13-543 | 95 | 12-499 | 1.25 | 12-503 | 1.25 | 12-497 | 1.25 | 12-504 | 1.25 | | | | | | | | | | | | |
| veg_mxtr_s_dunno_fat | 3.47 | 0.72 | 1.19 | 0.93 | 13-543 | 95 | 12-503 | 2.5 | 12-504 | 2.5 | | | | | | | | | | | | | | | | |
| veg_mxtr_s_dunno_lowfat | 2.37 | 0.46 | 0.72 | 0.62 | 13-543 | 95 | 12-497 | 2.5 | 12-499 | 2.5 | | | | | | | | | | | | | | | | |
| veg_mxtr_s_dunno_vlowfat | 2.37 | 0.46 | 0.72 | 0.62 | 13-543 | 95 | 12-497 | 2.5 | 12-499 | 2.5 | | | | | | | | | | | | | | | | |
| veg_mxtr_s_olive_chol | 2.42 | 0.45 | 1.08 | 0.32 | 13-543 | 95 | 12-498 | 5 | | | | | | | | | | | | | | | | | | |
| veg_mxtr_s_olive_dunno | 2.93 | 0.55 | 1.33 | 0.45 | 13-543 | 95 | 12-498 | 2.5 | 12-505 | 2.5 | | | | | | | | | | | | | | | | |
| veg_mxtr_s_olive_fat | 3.43 | 0.66 | 1.57 | 0.58 | 13-543 | 95 | 12-505 | 5 | | | | | | | | | | | | | | | | | | |
| veg_mxtr_s_olive_lowfat | 2.42 | 0.45 | 1.08 | 0.32 | 13-543 | 95 | 12-498 | 5 | | | | | | | | | | | | | | | | | | |
| veg_mxtr_s_olive_vlowfat | 2.42 | 0.45 | 1.08 | 0.32 | 13-543 | 95 | 12-498 | 5 | | | | | | | | | | | | | | | | | | |
| veg_onion_butter_dunno | 2.19 | 1.08 | 0.63 | 0.24 | 13-499 | 20 | 13-589 | 77.5 | 17-655 | 1.25 | 17-685 | 0.625 | 17-661 | 0.625 | | | | | | | | | | | | |
| veg_onion_butter_fat | 2.23 | 1.30 | 0.52 | 0.15 | 13-499 | 20 | 13-589 | 77.5 | 17-685 | 1.25 | 17-661 | 1.25 | | | | | | | | | | | | | | |
| veg_onion_butter_lowfat | 1.68 | 0.64 | 0.57 | 0.28 | 13-499 | 20 | 13-589 | 77.5 | 17-656 | 2.5 | | | | | | | | | | | | | | | | |
| veg_onion_butter_spread_fat | 2.15 | 0.85 | 0.74 | 0.33 | 13-499 | 20 | 13-589 | 77.5 | 17-655 | 2.5 | | | | | | | | | | | | | | | | |
| veg_onion_butter_spread_lowfat | 1.68 | 0.64 | 0.57 | 0.28 | 13-499 | 20 | 13-589 | 77.5 | 17-656 | 2.5 | | | | | | | | | | | | | | | | |
| veg_onion_fat_dunno | 2.67 | 0.17 | 1.48 | 0.82 | 13-499 | 20 | 13-589 | 77.5 | 17-686 | 2.5 | | | | | | | | | | | | | | | | |
| veg_onion_fat_none | 0.18 | 0.00 | 0.00 | 0.09 | 13-499 | 20 | 13-589 | 80 | | | | | | | | | | | | | | | | | | |
| veg_onion_fat_other | 2.66 | 1.41 | 0.83 | 0.16 | 13-499 | 20 | 13-589 | 77.5 | 81204000 | 1.25 | 17-487 | 1.25 | | | | | | | | | | | | | | |
| veg_onion_lard | 2.65 | 1.01 | 1.09 | 0.33 | 13-499 | 20 | 13-589 | 77.5 | 17-010 | 2.5 | | | | | | | | | | | | | | | | |
| veg_onion_marg_hard | 2.09 | 0.66 | 0.85 | 0.39 | 13-499 | 20 | 13-589 | 77.5 | 12-500 | 2.5 | | | | | | | | | | | | | | | | |
| veg_onion_marg_poly_chol | 1.10 | 0.21 | 0.29 | 0.46 | 13-499 | 20 | 13-589 | 77.5 | 12-499 | 2.5 | | | | | | | | | | | | | | | | |
| veg_onion_marg_poly_dunno | 1.38 | 0.27 | 0.37 | 0.59 | 13-499 | 20 | 13-589 | 77.5 | 12-499 | 1.25 | 12-503 | 1.25 | | | | | | | | | | | | | | |
| veg_onion_marg_poly_fat | 1.66 | 0.33 | 0.44 | 0.71 | 13-499 | 20 | 13-589 | 77.5 | 12-503 | 2.5 | | | | | | | | | | | | | | | | |
| veg_onion_marg_poly_lowfat | 1.10 | 0.21 | 0.29 | 0.46 | 13-499 | 20 | 13-589 | 77.5 | 12-499 | 2.5 | | | | | | | | | | | | | | | | |
| veg_onion_marg_poly_vlowfat | 1.10 | 0.21 | 0.29 | 0.46 | 13-499 | 20 | 13-589 | 77.5 | 12-499 | 2.5 | | | | | | | | | | | | | | | | |
| veg_onion_marg_soya_chol | 1.10 | 0.21 | 0.29 | 0.46 | 13-499 | 20 | 13-589 | 77.5 | 12-499 | 2.5 | | | | | | | | | | | | | | | | |
| veg_onion_marg_soya_dunno | 1.38 | 0.27 | 0.37 | 0.59 | 13-499 | 20 | 13-589 | 77.5 | 12-499 | 1.25 | 12-503 | 1.25 | | | | | | | | | | | | | | |
| veg_onion_marg_soya_fat | 1.66 | 0.33 | 0.44 | 0.71 | 13-499 | 20 | 13-589 | 77.5 | 12-503 | 2.5 | | | | | | | | | | | | | | | | |
| veg_onion_marg_soya_lowfat | 1.10 | 0.21 | 0.29 | 0.46 | 13-499 | 20 | 13-589 | 77.5 | 12-499 | 2.5 | | | | | | | | | | | | | | | | |
| veg_onion_marg_soya_vlowfat | 1.10 | 0.21 | 0.29 | 0.46 | 13-499 | 20 | 13-589 | 77.5 | 12-499 | 2.5 | | | | | | | | | | | | | | | | |
| veg_onion_oil_olive | 2.67 | 0.36 | 1.83 | 0.29 | 13-499 | 20 | 13-589 | 77.5 | 17-038 | 2.5 | | | | | | | | | | | | | | | | |
| veg_onion_oil_other | 2.36 | 0.27 | 1.00 | 0.90 | 13-499 | 20 | 13-589 | 77.5 | 17-033 | 0.75 | 17-040 | 0.5 | 17-686 | 0.938 | 17-377 | 0.312 | | | | | | | | | | |
| veg_onion_oil_rapeseed | 2.67 | 0.17 | 1.48 | 0.82 | 13-499 | 20 | 13-589 | 77.5 | 17-041 | 2.5 | | | | | | | | | | | | | | | | |
| veg_onion_oil_sunflower | 2.67 | 0.30 | 0.51 | 1.67 | 13-499 | 20 | 13-589 | 77.5 | 17-045 | 2.5 | | | | | | | | | | | | | | | | |
| veg_onion_oil_veg | 2.67 | 0.17 | 1.48 | 0.82 | 13-499 | 20 | 13-589 | 77.5 | 17-686 | 2.5 | | | | | | | | | | | | | | | | |
| veg_onion_spread_dairy_chol | 1.15 | 0.24 | 0.43 | 0.33 | 13-499 | 20 | 13-589 | 77.5 | 12-497 | 2.5 | | | | | | | | | | | | | | | | |
| veg_onion_spread_dairy_dunno | 1.42 | 0.32 | 0.59 | 0.36 | 13-499 | 20 | 13-589 | 77.5 | 12-497 | 1.25 | 12-504 | 1.25 | | | | | | | | | | | | | | |
| veg_onion_spread_dairy_fat | 1.69 | 0.39 | 0.75 | 0.38 | 13-499 | 20 | 13-589 | 77.5 | 12-504 | 2.5 | | | | | | | | | | | | | | | | |
| veg_onion_spread_dairy_lowfat | 1.15 | 0.24 | 0.43 | 0.33 | 13-499 | 20 | 13-589 | 77.5 | 12-497 | 2.5 | | | | | | | | | | | | | | | | |
| veg_onion_spread_dairy_vlowfat | 1.15 | 0.24 | 0.43 | 0.33 | 13-499 | 20 | 13-589 | 77.5 | 12-497 | 2.5 | | | | | | | | | | | | | | | | |
| veg_onion_spread_dunno_chol | 1.12 | 0.23 | 0.36 | 0.39 | 13-499 | 20 | 13-589 | 77.5 | 12-499 | 1.25 | 12-497 | 1.25 | | | | | | | | | | | | | | |
| veg_onion_spread_dunno_dunno | 1.40 | 0.29 | 0.48 | 0.47 | 13-499 | 20 | 13-589 | 77.5 | 12-499 | 0.625 | 12-503 | 0.625 | 12-497 | 0.625 | 12-504 | 0.625 | | | | | | | | | | |
| veg_onion_spread_dunno_fat | 1.67 | 0.36 | 0.60 | 0.55 | 13-499 | 20 | 13-589 | 77.5 | 12-503 | 1.25 | 12-504 | 1.25 | | | | | | | | | | | | | | |
| veg_onion_spread_dunno_lowfat | 1.12 | 0.23 | 0.36 | 0.39 | 13-499 | 20 | 13-589 | 77.5 | 12-499 | 1.25 | 12-497 | 1.25 | | | | | | | | | | | | | | |
| veg_onion_spread_dunno_vlowfat | 1.12 | 0.23 | 0.36 | 0.39 | 13-499 | 20 | 13-589 | 77.5 | 12-499 | 1.25 | 12-497 | 1.25 | | | | | | | | | | | | | | |
| veg_onion_spread_olive_chol | 1.15 | 0.22 | 0.54 | 0.24 | 13-499 | 20 | 13-589 | 77.5 | 12-498 | 2.5 | | | | | | | | | | | | | | | | |
| veg_onion_spread_olive_dunno | 1.40 | 0.28 | 0.66 | 0.31 | 13-499 | 20 | 13-589 | 77.5 | 12-498 | 1.25 | 12-505 | 1.25 | | | | | | | | | | | | | | |
| veg_onion_spread_olive_fat | 1.65 | 0.33 | 0.78 | 0.37 | 13-499 | 20 | 13-589 | 77.5 | 12-505 | 2.5 | | | | | | | | | | | | | | | | |
| veg_onion_spread_olive_lowfat | 1.15 | 0.22 | 0.54 | 0.24 | 13-499 | 20 | 13-589 | 77.5 | 12-498 | 2.5 | | | | | | | | | | | | | | | | |
| veg_onion_spread_olive_vlowfat | 1.15 | 0.22 | 0.54 | 0.24 | 13-499 | 20 | 13-589 | 77.5 | 12-498 | 2.5 | | | | | | | | | | | | | | | | |
| veg_other_butter_dunno | 2.80 | 1.36 | 0.80 | 0.34 | 13-542 | 5 | 13-638 | 42.5 | 13-549 | 5 | 13-585 | 5 | 13-161 | 34.5 | 13-656 | 5 | 17-655 | 1.5 | 17-685 | 0.75 | 17-661 | 0.75 | | | | |
| veg_other_butter_fat | 2.84 | 1.63 | 0.67 | 0.23 | 13-542 | 5 | 13-638 | 42.5 | 13-549 | 5 | 13-585 | 5 | 13-161 | 34.5 | 13-656 | 5 | 17-685 | 1.5 | 17-661 | 1.5 | | | | | | |
| veg_other_butter_lowfat | 2.18 | 0.84 | 0.72 | 0.38 | 13-542 | 5 | 13-638 | 42.5 | 13-549 | 5 | 13-585 | 5 | 13-161 | 34.5 | 13-656 | 5 | 17-656 | 3 | | | | | | | | |
| veg_other_butter_spread_fat | 2.18 | 0.84 | 0.72 | 0.38 | 13-542 | 5 | 13-638 | 42.5 | 13-549 | 5 | 13-585 | 5 | 13-161 | 34.5 | 13-656 | 5 | 17-656 | 3 | | | | | | | | |
| veg_other_butter_spread_lowfat | 2.75 | 1.10 | 0.93 | 0.44 | 13-542 | 5 | 13-638 | 42.5 | 13-549 | 5 | 13-585 | 5 | 13-161 | 34.5 | 13-656 | 5 | 17-655 | 3 | | | | | | | | |
| veg_other_fat_dunno | 3.38 | 0.27 | 1.82 | 1.02 | 13-542 | 5 | 13-638 | 42.5 | 13-549 | 5 | 13-585 | 5 | 13-161 | 34.5 | 13-656 | 5 | 17-686 | 3 | | | | | | | | |
| veg_other_fat_none | 0.39 | 0.07 | 0.04 | 0.15 | 13-542 | 5 | 13-638 | 44 | 13-549 | 5 | 13-585 | 5 | 13-161 | 36 | 13-656 | 5 | | | | | | | | | | |
| veg_other_fat_other | 3.36 | 1.76 | 1.04 | 0.23 | 13-542 | 5 | 13-638 | 42.5 | 13-549 | 5 | 13-585 | 5 | 13-161 | 34.5 | 13-656 | 5 | 81204000 | 1.5 | 17-487 | 1.5 | | | | | | |
| veg_other_lard | 3.35 | 1.28 | 1.34 | 0.44 | 13-542 | 5 | 13-638 | 42.5 | 13-549 | 5 | 13-585 | 5 | 13-161 | 34.5 | 13-656 | 5 | 17-010 | 3 | | | | | | | | |
| veg_other_marg_hard | 2.67 | 0.86 | 1.06 | 0.51 | 13-542 | 5 | 13-638 | 42.5 | 13-549 | 5 | 13-585 | 5 | 13-161 | 34.5 | 13-656 | 5 | 12-500 | 3 | | | | | | | | |
| veg_other_marg_poly_chol | 1.49 | 0.33 | 0.39 | 0.59 | 13-542 | 5 | 13-638 | 42.5 | 13-549 | 5 | 13-585 | 5 | 13-161 | 34.5 | 13-656 | 5 | 12-499 | 3 | | | | | | | | |
| veg_other_marg_poly_dunno | 1.82 | 0.40 | 0.48 | 0.74 | 13-542 | 5 | 13-638 | 42.5 | 13-549 | 5 | 13-585 | 5 | 13-161 | 34.5 | 13-656 | 5 | 12-499 | 1.5 | 12-503 | 1.5 | | | | | | |
| veg_other_marg_poly_fat | 2.15 | 0.47 | 0.57 | 0.90 | 13-542 | 5 | 13-638 | 42.5 | 13-549 | 5 | 13-585 | 5 | 13-161 | 34.5 | 13-656 | 5 | 12-503 | 3 | | | | | | | | |
| veg_other_marg_poly_lowfat | 1.49 | 0.33 | 0.39 | 0.59 | 13-542 | 5 | 13-638 | 42.5 | 13-549 | 5 | 13-585 | 5 | 13-161 | 34.5 | 13-656 | 5 | 12-499 | 3 | | | | | | | | |
| veg_other_marg_poly_vlowfat | 1.49 | 0.33 | 0.39 | 0.59 | 13-542 | 5 | 13-638 | 42.5 | 13-549 | 5 | 13-585 | 5 | 13-161 | 34.5 | 13-656 | 5 | 12-499 | 3 | | | | | | | | |
| veg_other_marg_soya_chol | 1.49 | 0.33 | 0.39 | 0.59 | 13-542 | 5 | 13-638 | 42.5 | 13-549 | 5 | 13-585 | 5 | 13-161 | 34.5 | 13-656 | 5 | 12-499 | 3 | | | | | | | | |
| veg_other_marg_soya_dunno | 1.82 | 0.40 | 0.48 | 0.74 | 13-542 | 5 | 13-638 | 42.5 | 13-549 | 5 | 13-585 | 5 | 13-161 | 34.5 | 13-656 | 5 | 12-499 | 1.5 | 12-503 | 1.5 | | | | | | |
| veg_other_marg_soya_fat | 2.15 | 0.47 | 0.57 | 0.90 | 13-542 | 5 | 13-638 | 42.5 | 13-549 | 5 | 13-585 | 5 | 13-161 | 34.5 | 13-656 | 5 | 12-503 | 3 | | | | | | | | |

**Table S1.** Individual FA and major nutrient calculation in the individual FA dataset (MCW + USDA). USDA food codes are highlighted in red.

| Food item | Grams per 100g of food item[1] | | | | Codes for foods from MCW and USDA and the fraction (%) assigned to each code[2] | | | | | | | | | | | | | | | | | | | | | |
|---|---|---|---|---|---|---|---|---|---|---|---|---|---|---|---|---|---|---|---|---|---|---|---|---|---|---|
| | Total fat | Total SFAs | Total MUFAs | Total PUFAs | Code 1 | Fraction 1 | Code 2 | Fraction 2 | Code 3 | Fraction 3 | Code 4 | Fraction 4 | Code 5 | Fraction 5 | Code 6 | Fraction 6 | Code 7 | Fraction 7 | Code 8 | Fraction 8 | Code 9 | Fraction 9 | Code 10 | Fraction 10 | Code 11 | Fraction 11 |
| veg_other_marg_soya_lowfat | 1.49 | 0.33 | 0.39 | 0.59 | 13-542 | 5 | 13-638 | 42.5 | 13-549 | 5 | 13-585 | 5 | 13-161 | 34.5 | 13-656 | 5 | 12-499 | 3 | | | | | | | | |
| veg_other_marg_soya_vlowfat | 1.49 | 0.33 | 0.39 | 0.59 | 13-542 | 5 | 13-638 | 42.5 | 13-549 | 5 | 13-585 | 5 | 13-161 | 34.5 | 13-656 | 5 | 12-499 | 3 | | | | | | | | |
| veg_other_oil_olive | 3.38 | 0.50 | 2.23 | 0.39 | 13-542 | 5 | 13-638 | 42.5 | 13-549 | 5 | 13-585 | 5 | 13-161 | 34.5 | 13-656 | 5 | 17-038 | 3 | | | | | | | | |
| veg_other_oil_other | 3.00 | 0.39 | 1.25 | 1.12 | 13-542 | 5 | 13-638 | 42.5 | 13-549 | 5 | 13-585 | 5 | 13-161 | 34.5 | 13-656 | 5 | 17-033 | 0.9 | 17-040 | 0.6 | 17-686 | 0.75 | 17-686 | 0.375 | 17-377 | 0.375 |
| veg_other_oil_rapeseed | 3.38 | 0.27 | 1.82 | 1.02 | 13-542 | 5 | 13-638 | 42.5 | 13-549 | 5 | 13-585 | 5 | 13-161 | 34.5 | 13-656 | 5 | 17-041 | 3 | | | | | | | | |
| veg_other_oil_sunflower | 3.38 | 0.43 | 0.66 | 2.04 | 13-542 | 5 | 13-638 | 42.5 | 13-549 | 5 | 13-585 | 5 | 13-161 | 34.5 | 13-656 | 5 | 17-045 | 3 | | | | | | | | |
| veg_other_oil_veg | 3.38 | 0.27 | 1.82 | 1.02 | 13-542 | 5 | 13-638 | 42.5 | 13-549 | 5 | 13-585 | 5 | 13-161 | 34.5 | 13-656 | 5 | 17-686 | 3 | | | | | | | | |
| veg_other_spread_dairy_chol | 1.55 | 0.36 | 0.56 | 0.44 | 13-542 | 5 | 13-638 | 42.5 | 13-549 | 5 | 13-585 | 5 | 13-161 | 34.5 | 13-656 | 5 | 12-497 | 3 | | | | | | | | |
| veg_other_spread_dairy_dunno | 1.87 | 0.45 | 0.75 | 0.47 | 13-542 | 5 | 13-638 | 42.5 | 13-549 | 5 | 13-585 | 5 | 13-161 | 34.5 | 13-656 | 5 | 12-497 | 1.5 | 12-504 | 1.5 | | | | | | |
| veg_other_spread_dairy_fat | 2.20 | 0.54 | 0.94 | 0.50 | 13-542 | 5 | 13-638 | 42.5 | 13-549 | 5 | 13-585 | 5 | 13-161 | 34.5 | 13-656 | 5 | 12-504 | 3 | | | | | | | | |
| veg_other_spread_dairy_lowfat | 1.55 | 0.36 | 0.56 | 0.44 | 13-542 | 5 | 13-638 | 42.5 | 13-549 | 5 | 13-585 | 5 | 13-161 | 34.5 | 13-656 | 5 | 12-497 | 3 | | | | | | | | |
| veg_other_spread_dairy_vlowfat | 1.55 | 0.36 | 0.56 | 0.44 | 13-542 | 5 | 13-638 | 42.5 | 13-549 | 5 | 13-585 | 5 | 13-161 | 34.5 | 13-656 | 5 | 12-497 | 3 | | | | | | | | |
| veg_other_spread_dunno_chol | 1.52 | 0.35 | 0.48 | 0.51 | 13-542 | 5 | 13-638 | 42.5 | 13-549 | 5 | 13-585 | 5 | 13-161 | 34.5 | 13-656 | 5 | 12-499 | 1.5 | 12-497 | 1.5 | | | | | | |
| veg_other_spread_dunno_dunno | 1.85 | 0.42 | 0.62 | 0.61 | 13-542 | 5 | 13-638 | 42.5 | 13-549 | 5 | 13-585 | 5 | 13-161 | 34.5 | 13-656 | 5 | 12-499 | 0.75 | 12-503 | 0.75 | 12-497 | 0.75 | 12-504 | 0.75 | | |
| veg_other_spread_dunno_fat | 2.18 | 0.50 | 0.76 | 0.70 | 13-542 | 5 | 13-638 | 42.5 | 13-549 | 5 | 13-585 | 5 | 13-161 | 34.5 | 13-656 | 5 | 12-503 | 1.5 | 12-504 | 1.5 | | | | | | |
| veg_other_spread_dunno_lowfat | 1.52 | 0.35 | 0.48 | 0.51 | 13-542 | 5 | 13-638 | 42.5 | 13-549 | 5 | 13-585 | 5 | 13-161 | 34.5 | 13-656 | 5 | 12-497 | 1.5 | 12-499 | 1.5 | | | | | | |
| veg_other_spread_dunno_vlowfat | 1.52 | 0.35 | 0.48 | 0.51 | 13-542 | 5 | 13-638 | 42.5 | 13-549 | 5 | 13-585 | 5 | 13-161 | 34.5 | 13-656 | 5 | 12-497 | 1.5 | 12-499 | 1.5 | | | | | | |
| veg_other_spread_olive_chol | 1.55 | 0.34 | 0.69 | 0.33 | 13-542 | 5 | 13-638 | 42.5 | 13-549 | 5 | 13-585 | 5 | 13-161 | 34.5 | 13-656 | 5 | 12-498 | 3 | | | | | | | | |
| veg_other_spread_olive_dunno | 1.85 | 0.40 | 0.84 | 0.41 | 13-542 | 5 | 13-638 | 42.5 | 13-549 | 5 | 13-585 | 5 | 13-161 | 34.5 | 13-656 | 5 | 12-498 | 1.5 | 12-505 | 1.5 | | | | | | |
| veg_other_spread_olive_fat | 2.15 | 0.47 | 0.98 | 0.49 | 13-542 | 5 | 13-638 | 42.5 | 13-549 | 5 | 13-585 | 5 | 13-161 | 34.5 | 13-656 | 5 | 12-505 | 3 | | | | | | | | |
| veg_other_spread_olive_lowfat | 1.55 | 0.34 | 0.69 | 0.33 | 13-542 | 5 | 13-638 | 42.5 | 13-549 | 5 | 13-585 | 5 | 13-161 | 34.5 | 13-656 | 5 | 12-498 | 3 | | | | | | | | |
| veg_other_spread_olive_vlowfat | 1.55 | 0.34 | 0.69 | 0.33 | 13-542 | 5 | 13-638 | 42.5 | 13-549 | 5 | 13-585 | 5 | 13-161 | 34.5 | 13-656 | 5 | 12-498 | 3 | | | | | | | | |
| veg_par_butter_dunno | 2.31 | 1.13 | 0.74 | 0.21 | 75222010 | 97.5 | 17-655 | 1.25 | 17-685 | 0.625 | 17-661 | 0.625 | | | | | | | | | | | | | | |
| veg_par_butter_fat | 2.35 | 1.35 | 0.63 | 0.12 | 75222010 | 97.5 | 17-685 | 1.25 | 17-661 | 1.25 | | | | | | | | | | | | | | | | |
| veg_par_butter_lowfat | 1.80 | 0.69 | 0.67 | 0.24 | 75222010 | 97.5 | 17-656 | 2.5 | | | | | | | | | | | | | | | | | | |
| veg_par_butter_spread_fat | 2.27 | 0.90 | 0.85 | 0.30 | 75222010 | 97.5 | 17-655 | 2.5 | | | | | | | | | | | | | | | | | | |
| veg_par_butter_spread_lowfat | 1.80 | 0.69 | 0.67 | 0.24 | 75222010 | 97.5 | 17-656 | 2.5 | | | | | | | | | | | | | | | | | | |
| veg_par_fat_dunno | 2.79 | 0.21 | 1.59 | 0.78 | 75222010 | 97.5 | 17-686 | 2.5 | | | | | | | | | | | | | | | | | | |
| veg_par_fat_none | 0.30 | 0.05 | 0.11 | 0.05 | 75222010 | 100 | | | | | | | | | | | | | | | | | | | | |
| veg_par_fat_other | 2.77 | 1.46 | 0.94 | 0.12 | 75222010 | 97.5 | 81204000 | 1.25 | 17-487 | 1.25 | | | | | | | | | | | | | | | | |
| veg_par_lard | 2.77 | 1.06 | 1.19 | 0.30 | 75222010 | 97.5 | 17-010 | 2.5 | | | | | | | | | | | | | | | | | | |
| veg_par_marg_hard | 2.20 | 0.71 | 0.96 | 0.35 | 75222010 | 97.5 | 12-500 | 2.5 | | | | | | | | | | | | | | | | | | |
| veg_par_marg_poly_chol | 1.22 | 0.26 | 0.40 | 0.42 | 75222010 | 97.5 | 12-499 | 2.5 | | | | | | | | | | | | | | | | | | |
| veg_par_marg_poly_dunno | 1.49 | 0.32 | 0.47 | 0.55 | 75222010 | 97.5 | 12-499 | 1.25 | 12-503 | 1.25 | | | | | | | | | | | | | | | | |
| veg_par_marg_poly_fat | 1.77 | 0.38 | 0.55 | 0.68 | 75222010 | 97.5 | 12-503 | 2.5 | | | | | | | | | | | | | | | | | | |
| veg_par_marg_poly_lowfat | 1.22 | 0.26 | 0.40 | 0.42 | 75222010 | 97.5 | 12-499 | 2.5 | | | | | | | | | | | | | | | | | | |
| veg_par_marg_poly_vlowfat | 1.22 | 0.26 | 0.40 | 0.42 | 75222010 | 97.5 | 12-499 | 2.5 | | | | | | | | | | | | | | | | | | |
| veg_par_marg_soya_chol | 1.22 | 0.26 | 0.40 | 0.42 | 75222010 | 97.5 | 12-499 | 2.5 | | | | | | | | | | | | | | | | | | |
| veg_par_marg_soya_dunno | 1.49 | 0.32 | 0.47 | 0.55 | 75222010 | 97.5 | 12-499 | 1.25 | 12-503 | 1.25 | | | | | | | | | | | | | | | | |
| veg_par_marg_soya_fat | 1.77 | 0.38 | 0.55 | 0.68 | 75222010 | 97.5 | 12-503 | 2.5 | | | | | | | | | | | | | | | | | | |
| veg_par_marg_soya_lowfat | 1.22 | 0.26 | 0.40 | 0.42 | 75222010 | 97.5 | 12-499 | 2.5 | | | | | | | | | | | | | | | | | | |
| veg_par_marg_soya_vlowfat | 1.22 | 0.26 | 0.40 | 0.42 | 75222010 | 97.5 | 12-499 | 2.5 | | | | | | | | | | | | | | | | | | |
| veg_par_oil_olive | 2.79 | 0.41 | 1.93 | 0.25 | 75222010 | 97.5 | 17-038 | 2.5 | | | | | | | | | | | | | | | | | | |
| veg_par_oil_other | 2.48 | 0.32 | 1.11 | 0.86 | 75222010 | 97.5 | 17-033 | 0.75 | 17-040 | 0.5 | 17-686 | 0.938 | 17-377 | 0.312 | | | | | | | | | | | | |
| veg_par_oil_rapeseed | 2.79 | 0.21 | 1.59 | 0.78 | 75222010 | 97.5 | 17-041 | 2.5 | | | | | | | | | | | | | | | | | | |
| veg_par_oil_sunflower | 2.79 | 0.35 | 0.62 | 1.63 | 75222010 | 97.5 | 17-045 | 2.5 | | | | | | | | | | | | | | | | | | |
| veg_par_oil_veg | 2.79 | 0.21 | 1.59 | 0.78 | 75222010 | 97.5 | 17-686 | 2.5 | | | | | | | | | | | | | | | | | | |
| veg_par_s_dairy_chol | 1.27 | 0.29 | 0.54 | 0.29 | 75222010 | 97.5 | 12-497 | 2.5 | | | | | | | | | | | | | | | | | | |
| veg_par_s_dairy_dunno | 1.54 | 0.37 | 0.70 | 0.32 | 75222010 | 97.5 | 12-497 | 1.25 | 12-504 | 1.25 | | | | | | | | | | | | | | | | |
| veg_par_s_dairy_fat | 1.81 | 0.44 | 0.86 | 0.34 | 75222010 | 97.5 | 12-504 | 2.5 | | | | | | | | | | | | | | | | | | |
| veg_par_s_dairy_lowfat | 1.27 | 0.29 | 0.54 | 0.29 | 75222010 | 97.5 | 12-497 | 2.5 | | | | | | | | | | | | | | | | | | |
| veg_par_s_dairy_vlowfat | 1.27 | 0.29 | 0.54 | 0.29 | 75222010 | 97.5 | 12-497 | 2.5 | | | | | | | | | | | | | | | | | | |
| veg_par_s_dunno_chol | 1.24 | 0.28 | 0.47 | 0.36 | 75222010 | 97.5 | 12-499 | 1.25 | 12-497 | 1.25 | | | | | | | | | | | | | | | | |
| veg_par_s_dunno_dunno | 1.52 | 0.34 | 0.59 | 0.43 | 75222010 | 97.5 | 12-499 | 0.625 | 12-503 | 0.625 | 12-497 | 0.625 | 12-504 | 0.625 | | | | | | | | | | | | |
| veg_par_s_dunno_fat | 1.79 | 0.41 | 0.70 | 0.51 | 75222010 | 97.5 | 12-503 | 1.25 | 12-504 | 1.25 | | | | | | | | | | | | | | | | |
| veg_par_s_dunno_lowfat | 1.24 | 0.28 | 0.47 | 0.36 | 75222010 | 97.5 | 12-499 | 1.25 | 12-497 | 1.25 | | | | | | | | | | | | | | | | |
| veg_par_s_dunno_vlowfat | 1.24 | 0.28 | 0.47 | 0.36 | 75222010 | 97.5 | 12-499 | 1.25 | 12-497 | 1.25 | | | | | | | | | | | | | | | | |
| veg_par_s_olive_chol | 1.27 | 0.27 | 0.65 | 0.20 | 75222010 | 97.5 | 12-498 | 2.5 | | | | | | | | | | | | | | | | | | |
| veg_par_s_olive_dunno | 1.52 | 0.33 | 0.77 | 0.27 | 75222010 | 97.5 | 12-498 | 1.25 | 12-505 | 1.25 | | | | | | | | | | | | | | | | |
| veg_par_s_olive_fat | 1.77 | 0.38 | 0.89 | 0.33 | 75222010 | 97.5 | 12-505 | 2.5 | | | | | | | | | | | | | | | | | | |
| veg_par_s_olive_lowfat | 1.27 | 0.27 | 0.65 | 0.20 | 75222010 | 97.5 | 12-498 | 2.5 | | | | | | | | | | | | | | | | | | |
| veg_par_s_olive_vlowfat | 1.27 | 0.27 | 0.65 | 0.20 | 75222010 | 97.5 | 12-498 | 2.5 | | | | | | | | | | | | | | | | | | |
| veg_peas | 0.70 | 0.13 | 0.07 | 0.36 | 13-527 | 100 | | | | | | | | | | | | | | | | | | | | |
| veg_pepp_butter_dunno | 2.27 | 1.11 | 0.63 | 0.28 | 13-548 | 19.5 | 13-524 | 19.5 | 13-526 | 19.5 | 13-525 | 19.5 | 13-537 | 19.5 | 17-655 | 1.25 | 17-685 | 0.625 | 17-661 | 0.625 | | | | | | |
| veg_pepp_butter_fat | 2.31 | 1.34 | 0.52 | 0.19 | 13-548 | 19.5 | 13-524 | 19.5 | 13-526 | 19.5 | 13-525 | 19.5 | 13-537 | 19.5 | 17-685 | 1.25 | 17-661 | 1.25 | | | | | | | | |
| veg_pepp_butter_lowfat | 1.76 | 0.68 | 0.57 | 0.31 | 13-548 | 19.5 | 13-524 | 19.5 | 13-526 | 19.5 | 13-525 | 19.5 | 13-537 | 19.5 | 17-656 | 2.5 | | | | | | | | | | |
| veg_pepp_butter_spread_fat | 2.23 | 0.89 | 0.74 | 0.37 | 13-548 | 19.5 | 13-524 | 19.5 | 13-526 | 19.5 | 13-525 | 19.5 | 13-537 | 19.5 | 17-655 | 2.5 | | | | | | | | | | |
| veg_pepp_butter_spread_lowfat | 1.76 | 0.68 | 0.57 | 0.31 | 13-548 | 19.5 | 13-524 | 19.5 | 13-526 | 19.5 | 13-525 | 19.5 | 13-537 | 19.5 | 17-656 | 2.5 | | | | | | | | | | |
| veg_pepp_fat_dunno | 2.75 | 0.20 | 1.48 | 0.85 | 13-548 | 19.5 | 13-524 | 19.5 | 13-526 | 19.5 | 13-525 | 19.5 | 13-537 | 19.5 | 17-686 | 2.5 | | | | | | | | | | |
| veg_pepp_fat_none | 0.26 | 0.04 | 0.00 | 0.12 | 13-548 | 20 | 13-524 | 20 | 13-526 | 20 | 13-525 | 20 | 13-537 | 20 | | | | | | | | | | | | |
| veg_pepp_fat_other | 2.73 | 1.44 | 0.83 | 0.19 | 13-548 | 19.5 | 13-524 | 19.5 | 13-526 | 19.5 | 13-525 | 19.5 | 13-537 | 19.5 | 81204000 | 1.25 | 17-487 | 1.25 | | | | | | | | |
| veg_pepp_lard | 2.73 | 1.04 | 1.09 | 0.37 | 13-548 | 19.5 | 13-524 | 19.5 | 13-526 | 19.5 | 13-525 | 19.5 | 13-537 | 19.5 | 17-010 | 2.5 | | | | | | | | | | |
| veg_pepp_marg_hard | 2.16 | 0.70 | 0.85 | 0.42 | 13-548 | 19.5 | 13-524 | 19.5 | 13-526 | 19.5 | 13-525 | 19.5 | 13-537 | 19.5 | 12-500 | 2.5 | | | | | | | | | | |
| veg_pepp_marg_poly_chol | 1.18 | 0.25 | 0.29 | 0.49 | 13-548 | 19.5 | 13-524 | 19.5 | 13-526 | 19.5 | 13-525 | 19.5 | 13-537 | 19.5 | 12-499 | 2.5 | | | | | | | | | | |
| veg_pepp_marg_poly_dunno | 1.45 | 0.31 | 0.37 | 0.62 | 13-548 | 19.5 | 13-524 | 19.5 | 13-526 | 19.5 | 13-525 | 19.5 | 13-537 | 19.5 | 12-499 | 1.25 | 12-503 | 1.25 | | | | | | | | |

**Table S1.** Individual FA and major nutrient calculation in the individual FA dataset (MCW + USDA). USDA food codes are highlighted in red.

| Food item | Grams per 100g of food item[1] | | | | Codes for foods from MCW and USDA and the fraction (%) assigned to each code[2] | | | | | | | | | | | | | | | | | | | | | |
|---|---|---|---|---|---|---|---|---|---|---|---|---|---|---|---|---|---|---|---|---|---|---|---|---|---|---|
| | Total fat | Total SFAs | Total MUFAs | Total PUFAs | Code 1 | Fraction 1 | Code 2 | Fraction 2 | Code 3 | Fraction 3 | Code 4 | Fraction 4 | Code 5 | Fraction 5 | Code 6 | Fraction 6 | Code 7 | Fraction 7 | Code 8 | Fraction 8 | Code 9 | Fraction 9 | Code 10 | Fraction 10 | Code 11 | Fraction 11 |
| veg_pepp_marg_poly_fat | 1.73 | 0.37 | 0.44 | 0.75 | 13-548 | 19.5 | 13-524 | 19.5 | 13-526 | 19.5 | 13-525 | 19.5 | 13-537 | 19.5 | 12-503 | 2.5 | | | | | | | | | | |
| veg_pepp_marg_poly_lowfat | 1.18 | 0.25 | 0.29 | 0.49 | 13-548 | 19.5 | 13-524 | 19.5 | 13-526 | 19.5 | 13-525 | 19.5 | 13-537 | 19.5 | 12-499 | 2.5 | | | | | | | | | | |
| veg_pepp_marg_poly_vlowfat | 1.18 | 0.25 | 0.29 | 0.49 | 13-548 | 19.5 | 13-524 | 19.5 | 13-526 | 19.5 | 13-525 | 19.5 | 13-537 | 19.5 | 12-499 | 2.5 | | | | | | | | | | |
| veg_pepp_marg_soya_chol | 1.18 | 0.25 | 0.29 | 0.49 | 13-548 | 19.5 | 13-524 | 19.5 | 13-526 | 19.5 | 13-525 | 19.5 | 13-537 | 19.5 | 12-499 | 2.5 | | | | | | | | | | |
| veg_pepp_marg_soya_dunno | 1.45 | 0.31 | 0.37 | 0.62 | 13-548 | 19.5 | 13-524 | 19.5 | 13-526 | 19.5 | 13-525 | 19.5 | 13-537 | 19.5 | 12-499 | 1.25 | 12-503 | 1.25 | | | | | | | | |
| veg_pepp_marg_soya_fat | 1.73 | 0.37 | 0.44 | 0.75 | 13-548 | 19.5 | 13-524 | 19.5 | 13-526 | 19.5 | 13-525 | 19.5 | 13-537 | 19.5 | 12-503 | 2.5 | | | | | | | | | | |
| veg_pepp_marg_soya_lowfat | 1.18 | 0.25 | 0.29 | 0.49 | 13-548 | 19.5 | 13-524 | 19.5 | 13-526 | 19.5 | 13-525 | 19.5 | 13-537 | 19.5 | 12-499 | 2.5 | | | | | | | | | | |
| veg_pepp_marg_soya_vlowfat | 1.18 | 0.25 | 0.29 | 0.49 | 13-548 | 19.5 | 13-524 | 19.5 | 13-526 | 19.5 | 13-525 | 19.5 | 13-537 | 19.5 | 12-499 | 2.5 | | | | | | | | | | |
| veg_pepp_oil_olive | 2.75 | 0.39 | 1.83 | 0.32 | 13-548 | 19.5 | 13-524 | 19.5 | 13-526 | 19.5 | 13-525 | 19.5 | 13-537 | 19.5 | 17-038 | 2.5 | | | | | | | | | | |
| veg_pepp_oil_other | 2.44 | 0.31 | 1.00 | 0.93 | 13-548 | 19.5 | 13-524 | 19.5 | 13-526 | 19.5 | 13-525 | 19.5 | 13-537 | 19.5 | 17-033 | 0.75 | 17-040 | 0.5 | 17-686 | 0.938 | 17-377 | 0.312 | | | | |
| veg_pepp_oil_rapeseed | 2.75 | 0.20 | 1.48 | 0.85 | 13-548 | 19.5 | 13-524 | 19.5 | 13-526 | 19.5 | 13-525 | 19.5 | 13-537 | 19.5 | 17-041 | 2.5 | | | | | | | | | | |
| veg_pepp_oil_sunflower | 2.75 | 0.34 | 0.51 | 1.70 | 13-548 | 19.5 | 13-524 | 19.5 | 13-526 | 19.5 | 13-525 | 19.5 | 13-537 | 19.5 | 17-045 | 2.5 | | | | | | | | | | |
| veg_pepp_oil_veg | 2.75 | 0.20 | 1.48 | 0.85 | 13-548 | 19.5 | 13-524 | 19.5 | 13-526 | 19.5 | 13-525 | 19.5 | 13-537 | 19.5 | 17-686 | 2.5 | | | | | | | | | | |
| veg_pepp_s_dairy_chol | 1.23 | 0.28 | 0.43 | 0.36 | 13-548 | 19.5 | 13-524 | 19.5 | 13-526 | 19.5 | 13-525 | 19.5 | 13-537 | 19.5 | 12-497 | 2.5 | | | | | | | | | | |
| veg_pepp_s_dairy_dunno | 1.50 | 0.35 | 0.59 | 0.39 | 13-548 | 19.5 | 13-524 | 19.5 | 13-526 | 19.5 | 13-525 | 19.5 | 13-537 | 19.5 | 12-497 | 1.25 | 12-504 | 1.25 | | | | | | | | |
| veg_pepp_s_dairy_fat | 1.77 | 0.43 | 0.75 | 0.41 | 13-548 | 19.5 | 13-524 | 19.5 | 13-526 | 19.5 | 13-525 | 19.5 | 13-537 | 19.5 | 12-504 | 2.5 | | | | | | | | | | |
| veg_pepp_s_dairy_lowfat | 1.23 | 0.28 | 0.43 | 0.36 | 13-548 | 19.5 | 13-524 | 19.5 | 13-526 | 19.5 | 13-525 | 19.5 | 13-537 | 19.5 | 12-497 | 2.5 | | | | | | | | | | |
| veg_pepp_s_dairy_vlowfat | 1.23 | 0.28 | 0.43 | 0.36 | 13-548 | 19.5 | 13-524 | 19.5 | 13-526 | 19.5 | 13-525 | 19.5 | 13-537 | 19.5 | 12-497 | 2.5 | | | | | | | | | | |
| veg_pepp_s_dunno_chol | 1.20 | 0.26 | 0.36 | 0.43 | 13-548 | 19.5 | 13-524 | 19.5 | 13-526 | 19.5 | 13-525 | 19.5 | 13-537 | 19.5 | 12-499 | 1.25 | 12-497 | 1.25 | | | | | | | | |
| veg_pepp_s_dunno_dunno | 1.48 | 0.33 | 0.48 | 0.50 | 13-548 | 19.5 | 13-524 | 19.5 | 13-526 | 19.5 | 13-525 | 19.5 | 13-537 | 19.5 | 12-499 | 0.625 | 12-503 | 0.625 | 12-497 | 0.625 | 12-504 | 0.625 | | | | |
| veg_pepp_s_dunno_fat | 1.75 | 0.40 | 0.60 | 0.58 | 13-548 | 19.5 | 13-524 | 19.5 | 13-526 | 19.5 | 13-525 | 19.5 | 13-537 | 19.5 | 12-503 | 1.25 | 12-504 | 1.25 | | | | | | | | |
| veg_pepp_s_dunno_lowfat | 1.20 | 0.26 | 0.36 | 0.43 | 13-548 | 19.5 | 13-524 | 19.5 | 13-526 | 19.5 | 13-525 | 19.5 | 13-537 | 19.5 | 12-499 | 1.25 | 12-497 | 1.25 | | | | | | | | |
| veg_pepp_s_dunno_vlowfat | 1.20 | 0.26 | 0.36 | 0.43 | 13-548 | 19.5 | 13-524 | 19.5 | 13-526 | 19.5 | 13-525 | 19.5 | 13-537 | 19.5 | 12-499 | 1.25 | 12-497 | 1.25 | | | | | | | | |
| veg_pepp_s_olive_chol | 1.23 | 0.26 | 0.54 | 0.28 | 13-548 | 19.5 | 13-524 | 19.5 | 13-526 | 19.5 | 13-525 | 19.5 | 13-537 | 19.5 | 12-498 | 2.5 | | | | | | | | | | |
| veg_pepp_s_olive_dunno | 1.48 | 0.31 | 0.66 | 0.34 | 13-548 | 19.5 | 13-524 | 19.5 | 13-526 | 19.5 | 13-525 | 19.5 | 13-537 | 19.5 | 12-498 | 1.25 | 12-505 | 1.25 | | | | | | | | |
| veg_pepp_s_olive_fat | 1.73 | 0.37 | 0.78 | 0.41 | 13-548 | 19.5 | 13-524 | 19.5 | 13-526 | 19.5 | 13-525 | 19.5 | 13-537 | 19.5 | 12-505 | 2.5 | | | | | | | | | | |
| veg_pepp_s_olive_lowfat | 1.23 | 0.26 | 0.54 | 0.28 | 13-548 | 19.5 | 13-524 | 19.5 | 13-526 | 19.5 | 13-525 | 19.5 | 13-537 | 19.5 | 12-498 | 2.5 | | | | | | | | | | |
| veg_pepp_s_olive_vlowfat | 1.23 | 0.26 | 0.54 | 0.28 | 13-548 | 19.5 | 13-524 | 19.5 | 13-526 | 19.5 | 13-525 | 19.5 | 13-537 | 19.5 | 12-498 | 2.5 | | | | | | | | | | |
| veg_pulses | 1.20 | 0.14 | 0.24 | 0.53 | 41302120 | 50 | 41305000 | 25 | 13-559 | 25 | | | | | | | | | | | | | | | | |
| veg_saladmayo | 14.55 | 1.34 | 7.84 | 4.60 | 15-636 | 33.4 | 15-635 | 33.3 | 13-636 | 9.99 | 14-346 | 14.985 | 14-879 | 3.33 | 17-654 | 4.995 | | | | | | | | | | |
| veg_sidesalad | 0.10 | 0.02 | 0.02 | 0.05 | 13-520 | 25 | 13-517 | 50 | 13-245 | 25 | | | | | | | | | | | | | | | | |
| veg_spinach | 0.87 | 0.16 | 0.00 | 0.37 | 72125212 | 100 | | | | | | | | | | | | | | | | | | | | |
| veg_sprouts | 0.40 | 0.09 | 0.03 | 0.22 | 13-630 | 100 | | | | | | | | | | | | | | | | | | | | |
| veg_sweetcorn | 0.60 | 0.13 | 0.07 | 0.27 | 13-634 | 100 | | | | | | | | | | | | | | | | | | | | |
| veg_sweetpot | 0.30 | 0.14 | 0.00 | 0.09 | 13-672 | 50 | 13-646 | 50 | | | | | | | | | | | | | | | | | | |
| veg_tomato_fresh | 0.30 | 0.07 | 0.07 | 0.13 | 13-517 | 50 | 13-519 | 50 | | | | | | | | | | | | | | | | | | |
| veg_tomato_tinned | 1.65 | 0.22 | 0.73 | 0.60 | 13-530 | 50 | 13-518 | 48.5 | 17-045 | 0.75 | 17-038 | 0.75 | | | | | | | | | | | | | | |
| veg_turnip | 0.15 | 0.00 | 0.00 | 0.10 | 13-391 | 50 | 13-650 | 50 | | | | | | | | | | | | | | | | | | |
| veg_watercress | 0.39 | 0.06 | 0.01 | 0.17 | 72125100 | 100 | | | | | | | | | | | | | | | | | | | | |
| vegalt_burger | 7.75 | 1.39 | 3.64 | 1.91 | 41305000 | 23.75 | 41302120 | 23.75 | 17-041 | 2.5 | 15-658 | 50 | | | | | | | | | | | | | | |
| vegalt_other | 19.25 | 2.82 | 7.40 | 7.99 | 13-505 | 11.25 | 14-879 | 11.25 | 42104200 | 11.25 | 41305000 | 22.5 | 17-038 | 3 | 11-792 | 4.5 | 12-940 | 4.5 | 17-377 | 6.75 | 41302120 | 22.5 | 14-844 | 1.125 | 17-045 | 1.375 |
| vegalt_quorn | 7.71 | 0.96 | 1.55 | 4.83 | 13-505 | 55 | 17-377 | 20 | 12-960 | 10 | 17-045 | 7.5 | 11-886 | 7.5 | | | | | | | | | | | | |
| vegalt_tofu | 6.10 | 0.82 | 1.31 | 3.61 | 41420010 | 97.5 | 17-045 | 2.5 | | | | | | | | | | | | | | | | | | |
| yeast_extract | 0.40 | 0.00 | 0.00 | 0.00 | 17-517 | 100 | | | | | | | | | | | | | | | | | | | | |
| yogurt_fullfat | 4.44 | 2.92 | 1.11 | 0.13 | 12-184 | 40 | 12-375 | 40 | 12-555 | 20 | | | | | | | | | | | | | | | | |
| yogurt_lowfat | 0.80 | 0.52 | 0.21 | 0.02 | 12-379 | 75 | 12-532 | 25 | | | | | | | | | | | | | | | | | | |
| yorkshirepud | 8.22 | 2.02 | 2.02 | 3.49 | 11-886 | 25 | 12-940 | 15 | 12-596 | 38.5 | 12-307 | 16.5 | 17-045 | 5 | | | | | | | | | | | | |

FA, fatty acid; MCW, McCance and Widdowson; MUFA, monounsaturated fatty acid; PUFA, polyunsaturated fatty acid; SFA, saturated fatty acid; USDA, US Department of Agriculture. [1] Calculated as the sum of total fat, total SFAs, total MUFAs, or total PUFAs for each of the foods assigned to the food item (fraction used multiplied by grams of total fat, total SFAs, total MUFAs, or total PUFAs per 100g of food). [2] Fractions in the individual FA dataset were based on fractions from the main dataset for the Oxford WebQ [17], which were determined by taking into account how each question was asked and the most likely food choics in the UK population. Fractions assigned to codes 1 to 11
